# Engineering a Model Cell for Rational Tuning of GPCR Signaling

**DOI:** 10.1016/j.cell.2019.02.023

**Published:** 2019-04-18

**Authors:** William M. Shaw, Hitoshi Yamauchi, Jack Mead, Glen-Oliver F. Gowers, David J. Bell, David Öling, Niklas Larsson, Mark Wigglesworth, Graham Ladds, Tom Ellis

**Affiliations:** 1 Department of Bioengineering, Imperial College London, London SW7 2AZ, UK; 2 Centre for Synthetic Biology, Imperial College London, London SW7 2AZ, UK; 3 Department of Pharmacology, University of Cambridge, Cambridge CB2 1PD, UK; 4 SynbiCITE Innovation and Knowledge Centre, Imperial College London, London SW7 2AZ, UK; 5 Discovery Biology, Discovery Sciences, Innovative Medicines and Early Development Biotech Unit, AstraZeneca R&D, 431 50 Mölndal, Sweden; 6 Hit Discovery, Discovery Sciences, IMED Biotech Unit, AstraZeneca, Macclesfield SK10 4TG, UK

**Keywords:** synthetic biology, genome engineering, *Saccharomyces cerevisiae*, cell-to-cell communication, G protein-coupled receptor, cell signaling, biosensor

## Abstract

G protein-coupled receptor (GPCR) signaling is the primary method eukaryotes use to respond to specific cues in their environment. However, the relationship between stimulus and response for each GPCR is difficult to predict due to diversity in natural signal transduction architecture and expression. Using genome engineering in yeast, we constructed an insulated, modular GPCR signal transduction system to study how the response to stimuli can be predictably tuned using synthetic tools. We delineated the contributions of a minimal set of key components via computational and experimental refactoring, identifying simple design principles for rationally tuning the dose response. Using five different GPCRs, we demonstrate how this enables cells and consortia to be engineered to respond to desired concentrations of peptides, metabolites, and hormones relevant to human health. This work enables rational tuning of cell sensing while providing a framework to guide reprogramming of GPCR-based signaling in other systems.

## Highlights

• Genomic refactoring of a minimized GPCR signaling pathway in *S. cerevisiae*
• Altering expression levels of key signaling components provides pathway tunability
• Engineered microbial consortia provide desired dose-response characteristics
• Development of fine-tuned sensors for detecting molecules relevant to human health

## Graphical Abstract

**Figure:**
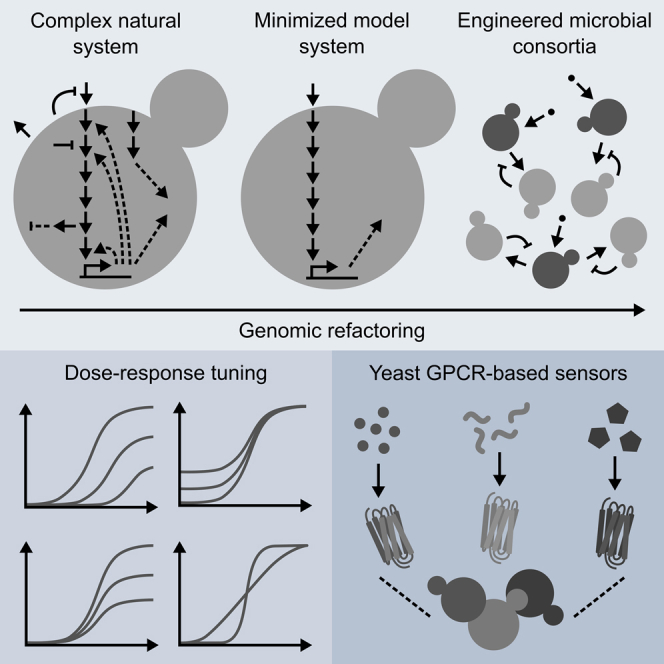

## Teaser

Engineering GPCR-based signaling in yeast allows exploration of signaling properties and creation of synthetic responses to a range of ligands.

## Introduction

G protein-coupled receptors (GPCRs) are widely represented in most lifeforms and comprise the largest family of signaling proteins in humans, with over 800 members detecting structurally diverse agonists (Fredriksson et al., 2003, Pierce et al., 2002). Their abundance and ubiquity to all cell types makes them one of the most important signaling pathway classes in healthcare but also one of the most complex (Marinissen and Gutkind, 2001, Santos et al., 2017). Multiple types of G protein-based signaling are seen and the downstream signal transduction to activate gene expression is typically complex and intertwined with other pathways (Kenakin, 2013, Neves, 2002). The nature of signal transduction through the pathway also depends on many different factors, including the stoichiometry of the signaling proteins, the presence of inherent feedback mechanisms, and even cellular history (Selbie and Hill, 1998). Altogether, this makes it difficult to delineate receptor and signaling properties simply from measuring the activation of downstream targets (Prezeau et al., 2010). It also makes it a major challenge to predict how changes in the levels of pathway components, for example due to different environments or mutations, can affect the performance of a given signaling pathway.

One of the most studied examples of eukaryotic GPCR signaling is the pheromone response pathway of *Saccharomyces cerevisiae* (Bardwell, 2004), having been the focus of significant efforts from systems biology to model its actions via quantification of its behavior (Yu et al., 2008). To understand this pathway, researchers have parsed the contributions of numerous studies that have perturbed the dose-response and dynamics of the native system by changing growth conditions, by protein mutagenesis, via traditional gene overexpression and knockout methods, and more recently using optogenetics (Alvaro and Thorner, 2016, Atay and Skotheim, 2017, Harrigan et al., 2018). While these efforts have helped to build our best picture of the events required for the transduction of signal from agonist to gene activation, inability to control the whole pathway in these experiments has meant that a complete system for exploring the dose-response relationship has not yet been achieved (Atay and Skotheim, 2017).

*In silico* approaches typically model a system by concentrating only on the key components and varying important parameters of these such as their expression levels, while removing other non-key interactions from consideration (Aldridge et al., 2006, Kholodenko, 2006). With advanced genome engineering and synthetic biology tools available, it now becomes possible to take an equivalent modeling approach *in vivo*, removing any non-essential interactions via gene knockout and finely tuning the expression of the key components using promoter libraries (Chan et al., 2005, Temme et al., 2012). This engineering approach—known as refactoring—makes a system easier to study by removing all non-essential natural regulation and feedback, thus enabling the system to be more efficiently tuned and directly measured. Effectively this generates cells streamlined for improved understanding of pathways and systems, while also making these cells more straightforward to utilize in downstream applications.

Here, we used genome engineering to construct a heavily modified yeast suitable as an *in vivo* model for tuning GPCR signaling. By removing non-essential components, native transcriptional feedback regulation, and all connections to the mating response, we built a model strain retaining only the core signaling elements. In conjunction with a mathematical model, we used promoter libraries to vary the key components in this simplified, refactored pathway and uncovered principles for tuning the sensitivity, basal activity, and signal amplitude of the dose-response curve via expression level. This new knowledge provides us with a rational approach for tuning signaling characteristics and, as we demonstrate, enables us to quickly reprogram yeast to sense and measure a variety of different inputs, either in single-cell systems or community-based systems.

## Results

### A Highly Engineered Model Strain for Probing the Signaling Pathway Response

Glucose sensing and the pheromone response pathway are the two native GPCR signaling pathways in *S. cerevisiae* (Versele et al., 2001), and the latter has long been the go-to choice for coupling heterologous GPCRs to yeast gene expression or for building systems for evolving GPCRs to desired targets (Dong et al., 2010, Ladds et al., 2005a). Core to this pathway is an extensively studied mitogen-activated protein kinase (MAPK) signaling cascade that functions with its own intrinsic feedback to maintain a robust input-output relationship in varying conditions (Chen and Thorner, 2007). Because this MAPK cascade displays a graded, linear response with respect to dose and can be considered as a black-box processing unit in transduction through the pathway (Bashor et al., 2008, Kofahl and Klipp, 2004, Poritz et al., 2001), we chose to make this natural system the core from which we build and tune GPCR signaling pathways.

**Figure 1:**
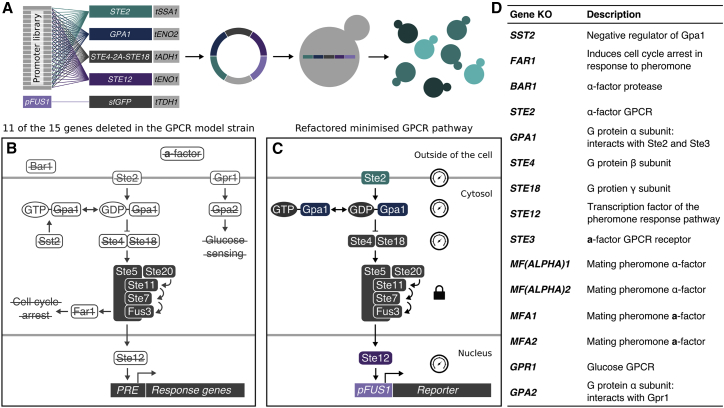
A Model GPCR Strain for Probing Pathway Performance (A) Pathway variants are generated by assembling the key signaling components into a single multigene cassette, using a library of well-characterized promoters to vary the expression, and then chromosomally integrating into the model strain, yWS1922, to reconstitute a minimized GPCR signaling pathway. (B) 11 of the 15 genes deleted from the yeast mating and glucose-sensing pathways in the model strain, leaving only the core signaling elements of the MAPK cascade intact. (C) A refactored signaling pathway, consisting of a minimized set of signaling components for transmitting a unidirectional signal from the cell surface to the nucleus. Gauges and padlocks represent components we have chosen to vary or keep fixed, respectively. (D) The 15 gene deletions in the model strain, serving six key purposes: (1) to remove negative feedback within the signaling pathway (*SST2*), (2) to prevent unwanted cell-cycle arrest (*FAR1*), (3) to prevent ɑ-factor signal degradation (*BAR1*), (4) to be refactored with synthetic tools (*STE2*, *GPA1*, *STE4*, *STE18*, and *STE12*), (5) to remove mechanisms for pheromone-based communication (*MF*(*ALPHA*)*1*+*2*, *MFA1*+*2*, and *STE3*), and (6) to remove all other instances of GPCR/G-protein signaling (*GPR1* and *GPA2*). See also Figure S1.

**Figure S1:**
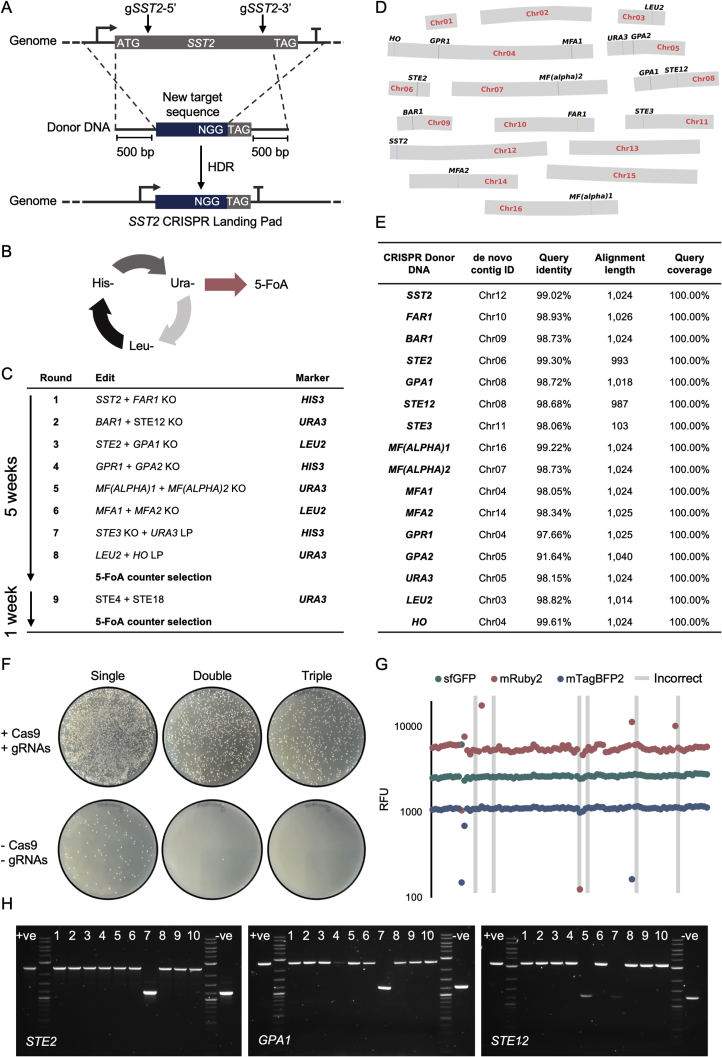
Cell Line Development, Related to Figure 1 (A-C) Strategy for engineering the GPCR model strains. (A) To generate the 15 gene KOs in Figure 1D, double strand breaks (DSBs) were generated between the start and stop codon of the ORF of each gene using CRISPR/Cas9. Donor DNA was provided as a template for homology-directed repair (HDR), comprising 500 bp homology arms flanking a unique 20 bp Cas9 targeting sequence followed by a CGG protospacer adjacent motif (PAM) sequence. The resulting KOs represent a precise substitution of the open reading frame with a unique landing pad (LP) to enable future editing at each locus. A further 3 edits were performed at the *URA3*, *LEU2*, and *HO* loci to prepare the cells for efficient multiplexed integration of YTK plasmids by installing a landing pad between the two arms of homology. (B) To rapidly iterate through the 18 genomic edits a marker cycling strategy was employed. Once an edit, facilitated by CRISPR/Cas9 carried on a 2μ plasmid containing one of the three auxotrophic markers, *URA3*, *LEU2* and *HIS3*, had been confirmed by colony PCR, the next set of edits would immediately be performed by transforming the donor DNA and CRISPR/Cas9 machinery supplied on a plasmid using the next marker in the cycle, removing the need to cure cells of the previous marker. A 3-marker cycle was sufficient to remove all trace of a marker before it was reused in the cycle. (C) Edits were implemented in a pairwise manner, totaling 8 rounds of editing to generate the yWS677 strain and an additional round to generate the yWS1922 strain. 12 colonies were screened for each pairwise edit, yielding at least one correct colony at each round. Editing of yWS677 and yWS1922 was followed by counterselection of the *URA3* CRISPR apparatus by 5-FoA to generate clean strains. The final strain was validated for loss of all CRISPR plasmids via replica plating on Ura-, Leu- and His- media and colony PCR. (D+E) Nanopore sequencing of the yWS677 stain (D) *De novo* contigs assembled using Smartdenovo from reads corrected by Canu, representing the full set of 16 chromosomes from *S. cerevisiae*, confirmed by exact alignment to S288C reference genome using a minimum alignment length of 100 bp. All discrepancies with the reference genome are highlighted and correspond to the 16 edits described in E. No other discrepancies were detected, suggesting precise CRISPR/Cas9 editing during the 8 rounds. (E) A list of the expected changes and confirmation of their correct positioning within the yWS677 genome. Note all alignments are approximately 1000 bp in length, as this was the size of the donor DNA transformed, except for *STE3*. Due to cloning issues with the *STE3* KO donor DNA, a smaller fragment generated from oligonucleotides was used instead. (F-H) Efficient (Re)integration of DNA at the genomic CRISPR landing pads in yWS677. (F) Single, double, and triple integration of *URA3*, *LEU2*, and *HIS3* maker cassettes at the *URA3*, *LEU2*, and *HO* loci, encoding sfGFP, mRuby2, and mTagBFP2, with and without Cas9 and the sgRNAs required to generate DSBs at their respective LP. (G) Green, red, and blue fluorescence of 96 random colonies from the CRIPSR-aided triple integration. 90/96 colonies correct for triple integration. The remaining 6 colonies contained a mixture of multiple integrations, or missing fluorescence proteins. Experimental measurements are sfGFP, mRuby2, and mTagBFP2 levels per cell determined by flow cytometry. (H) Colony PCR verification of the multiplexed re-integration of *STE2*, *GPA1*, and *STE12* into yWS677 using markerless CRISPR/Cas9 editing to create the Quasi-WT strain. 8/10 random transformants were correct for the triple gene re-integration. 5-FoA counter selection was used to cure a confirmed strain of the CRISPR plasmid, and direct Sanger sequencing of the 3 genes was performed to confirm their identity to validate the Quasi-WT strain.

Keeping the five genes of the MAPK cascade fixed, we set out to generate a model strain for our work by first removing all other GPCR pathway-related genes from *S. cerevisiae* (Figure 1). This required making precise changes at 18 genomic loci in BY4741 yeast, generating our model strain, yWS1922, via nine rounds of CRISPR/Cas9-mediated editing (Figures S1A–S1C). Genomic changes were validated at each round by PCR and locus sequencing followed by long-read nanopore sequencing of the final strain (Figures S1D and S1E).

During strain construction, we added addressable 24 bp targets in place of the open reading frames (ORFs) of all deleted genes. These allow for rapid and markerless (re)insertion of native or heterologous ORFs into these locations at high efficiency by CRISPR-aided multiplex integration (Figures S1F–S1H). For stable single-copy addition of further genes, three highly characterized landing pads were also introduced that interface with the MoClo Yeast Toolkit (YTK) modular cloning system, which enables rapid multigene construction from high-characterized parts (Lee et al., 2015). These changes were designed to facilitate rapid exploration of the effects of altering individual components of the pheromone response pathway.

**Figure S2:**
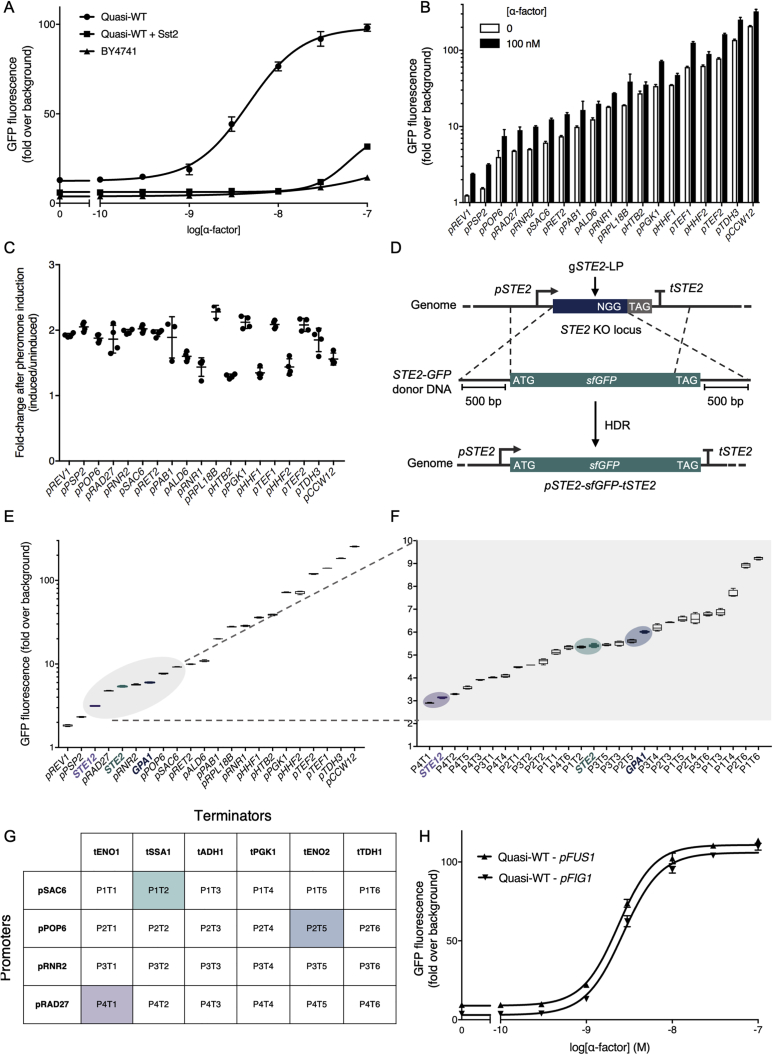
Initial Pathway Refactoring, Related to Figure 2 (A) ɑ-factor dose-response curve of the pheromone response pathway in BY4741 (WT yeast), Quasi-WT, and Quasi-WT overexpressing Sst2, using the *FUS1* promoter driving the expression of sfGFP. The poor response of BY4741 compared to Quasi-WT is due to the role of Sst2. (B-G) Here, we determine the promoter combinations for refactoring Ste2, Gpa1, and Ste12 in yWS677 (Ste4 and Ste18 were refactored in yWS1922 after establishing the expression of Ste2, Gpa1, and Ste12 in yWS677). To do this, we substituted the native *STE2*, *GPA1*, and *STE12* ORFs in yWS677 for the sfGFP ORF to serve as a proxy for native expression, enabling us to select promoter/terminator combinations from the YTK system for refactoring the minimized pheromone response pathway. (B) Before using YTK constitutive promoters, their performance under pheromone induction was measured to ensure it had no effect. 19 constitutive promoters from the YTK system were measured driving expression of sfGFP, with and without induction using 100 nM ɑ-factor peptide, in the Quasi-WT strain, after the standard assay time of 4 hours. (C) Relative fluorescence change (induced/uninduced) due to pheromone induction calculated from (B). A similar response was observed for all promoters, suggesting a common trait responsible for the increase in sfGFP fluorescence, likely caused by morphological changes as part of the pheromone response, leading to system wide increase in total protein. (D) Using LPs previously introduced at the gene KO sites, the sfGFP ORF was introduced between the native regulatory elements of *STE2*, *GPA1*, and *STE12* to serve as a proxy for relative gene expression. (E) The GFP fluorescence levels of the *STE2*, *GPA1*, and *STE12* ORF-GFP substitution strains were compared to the 19 constitutive promoters of the YTK system also driving the expression of sfGFP, integrated at the *URA3* locus of yWS677. The gray ellipse highlights 4 constitutive promoters with similar expression levels to the 3 genes; *pSAC6*, *pPOP6*, *pRNR2*, and *pRAD27*. (F) An expanded promoter/terminator library including all 6 terminators within the YTK toolkit in combination with the *pSAC6*, *pPOP6*, *pRNR2*, and *pRAD27* promoters to provide a larger profile of sfGFP expression levels to match with the native expression of *STE2*, *GPA1*, and *STE12*. (G) Selection of promoter/terminator combinations both unique to each other and similar to native expression of *STE2* (green), *GPA1* (blue), and *STE12* (purple) chosen for the initial refactoring of the minimized pheromone response pathway (Figure 3, Design 1). (H) Characterizing two of the most used pheromone responsive promoters. ɑ-factor dose-response curve of the pheromone response pathway Quasi-WT background using the *FUS1* and *FIG1* promoters. As *pFUS1* demonstrated more intrinsic leak this promoter was used to explore points of tuning in the pheromone response pathway, as discreet changes would be more measurable. Experimental measurements are sfGFP levels per cell determined by flow cytometry and shown as the mean ± standard deviation from triplicate isolates. Curves were fitted using GraphPad Prism variable slope (four parameter) nonlinear regression fit.

To determine how the model strain performed with all non-essential components removed, the native GPCR (*STE2*), Gα (*GPA1*), Gβ (*STE4*), Gγ (*STE18*), and pheromone responsive transcription factor (*STE12*) genes were restored at their natural loci to generate the “Quasi-WT” strain (Figure S1H). The α-factor dose-response of this strain was then compared to BY4741 yeast using the pheromone response *FUS1* promoter driving sfGFP expression (Hagen et al., 1991, Minic et al., 2005). As expected, a substantial increase in sensitivity and signal output was observed upon minimizing the signaling pathway, largely due to the removal of the native regulator of G protein signaling, Sst2 (Figure S2A).

### Tuning GPCR and G Protein Levels Alters Response Sensitivity and Basal Activity

**Figure 2:**
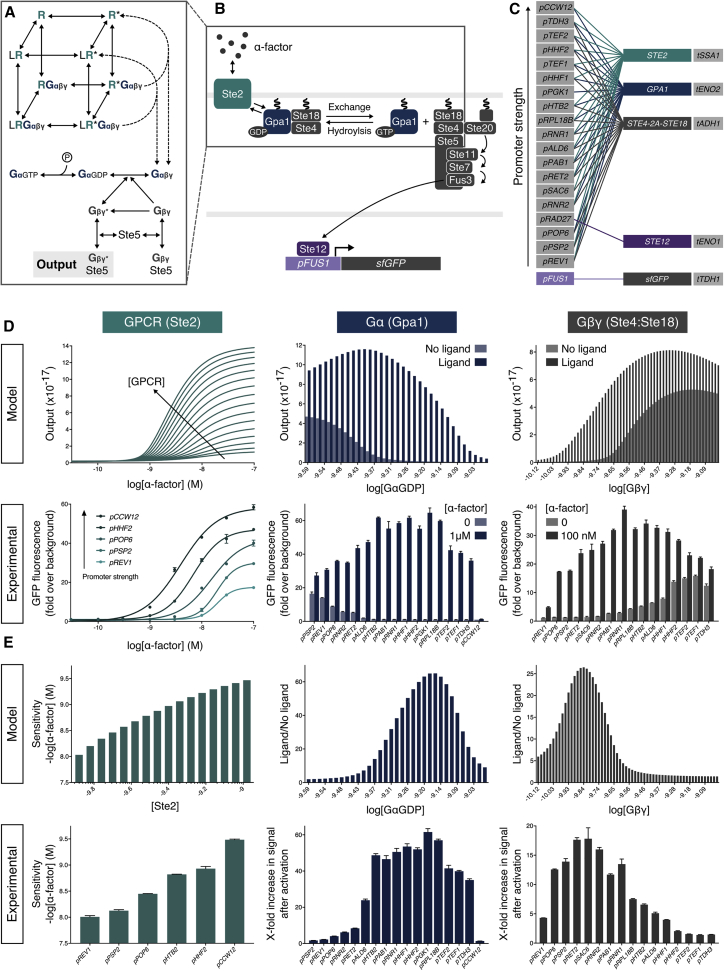
Model-Guided Tuning for Optimal G Protein Signaling (A) Cubic ternary complex model of G protein signaling in the minimized pheromone response pathway. (B) The minimized α-factor signaling pathway. Binding of the ligand (α-factor) to its specific GPCR (Ste2) on the cell surface leads to GDP-GTP exchange on the Gɑ subunit (Gpa1) and the release of the Gβγ dimer (Ste4 and Ste18), recruiting the MAP-kinase cascade to the membrane and facilitating the induction of the pathway via Ste20, ultimately resulting in the phosphorylation of the Ste12 transcription factor to induce gene expression via the pheromone responsive *FUS1* promoter. (C) Promoter combinations used for refactoring Ste2, Gpa1, and Ste4-2A-Ste18. (D) α-Factor dose-response characteristics from individually varying the GPCR (green), Gα (blue), and Gβγ (gray) concentrations computationally and expression levels experimentally. (E) Analysis of dose-response characteristics, demonstrating trends in the expression profiles of the refactored signaling components. Sensitivity is defined as the lowest concentration to produce a 2-fold change in GFP expression over background. See Figure S3 for a quantitative plot of Ste2, Gpa1, and Ste4-2A-Ste18 expression versus pathway output. Experimental measurements are sfGFP levels per cell determined by flow cytometry and shown as the mean ± SD from triplicate isolates. Curves were fitted using GraphPad Prism variable slope (four parameter) nonlinear regression fit. See also Figure S2.

Previous work has shown that the sensitivity of the yeast pheromone response pathway can be changed by altering the receptor number in the absence of Sst2 (Bush et al., 2016). Basal activity from constitutive receptor activity can also be reduced by overexpressing Gα (Bakker et al., 2001, Burstein et al., 1997), as this acts as a negative regulator of signaling since Gβγ propagates the response to the MAPK cascade (Bardwell, 2004). Therefore, to explore how sensitivity and basal activity could be varied by altering expression of the GPCR, Gα, and Gβγ, we built a mathematical model derived from a previously described cubic ternary complex model (Bridge et al., 2018), designing this to capture G protein signaling in our minimized pheromone response pathway (Figure 2A). We then systematically probed the response of the pathway in this model by individually altering the initial GPCR, Gα, and Gβγ concentrations, while keeping all other components fixed (Figures 2D and 2E, model).

While this model demonstrated a clear monotonic relationship between receptor number, sensitivity, and maximum signal, as previously shown by Bush et al. (2016), the relationship between Gα and Gβγ levels and pathway response was non-monotonic. At lower concentrations of Gα, constitutive activation of the pathway was observed due to increased free Gβγ. This, in combination with the incorrect trafficking and instability of Gβγ, due to low levels of Gα, leads to a lower maximum-fold change in activation (Hirschman et al., 1997, Song et al., 1996). At higher Gα concentrations, free Gβγ is rapidly sequestered, also leading to a decrease in the maximum fold change by acting as a “sponge” to signaling. A reverse behavior was seen when varying the initial concentration of Gβγ, with low concentrations decreasing maximum signaling and high concentrations leading to increased basal activity, albeit with a lower maximum signal, due to incorrect trafficking of free Gβγ, and therefore the Ste5 scaffold (Pryciak and Huntress, 1998). Taken together, the model predicts an optimal level of Gα and Gβγ expression where all three members of the heterotrimeric G protein are required to be in balance to give a high-fold change in signal upon activation.

**Figure S3:**
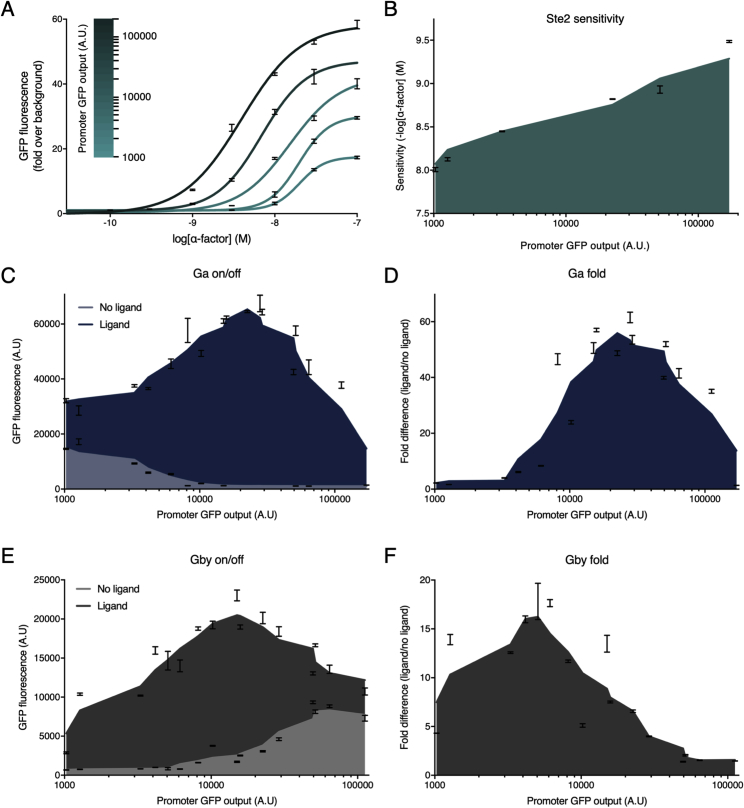
Quantitative Plots of Ste2, Gpa1, and Ste4-2A-Ste18 Expression versus Pathway Output, Related to Figure 2 (A) ɑ-factor dose-response of the minimized pathway designs where the intracellular levels of GPCR are varied using a promoter library driving the expression of Ste2. Promoter identity is plotted as heatmap of GFP fluorescence from data in Figure S2B. Experimental measurements are sfGFP levels per cell determined by flow cytometry and shown as the mean ± standard deviation from triplicate isolates. Curves were fitted using GraphPad Prism variable slope (four parameter) nonlinear regression fit. (B) Sensitivity of the Ste2 pathway variants to ɑ-factor. Sensitivity was determined from the fitted curve, defining sensitivity as the lowest concentration for which a > 2-fold change in GFP expression is seen. (C) Experimental ON/OFF response of the minimized pathway designs where the intracellular levels of Gɑ are varied using a promoter library driving the expression of Gpa1. (D) Experimental maximum x-fold change in signal for Gpa1 promoter library. (E) Experimental ON/OFF response of the minimized pathway designs where the intracellular levels of Gβγ are varied using a promoter library driving the expression of Ste4-2A-Ste18. (F) Experimental maximum x-fold change in signal for Ste4-2A-Ste18 promoter library. Promoter identity is plotted as x-value of GFP fluorescence from data in Figure S2B. Curves fitting using a 6^th^ order smoothing polynomial with 3 neighbors on each side. Experimental measurements are sfGFP levels per cell determined by flow cytometry and shown as the mean ± standard deviation from triplicate isolates.

Next, using the model strain and modular cloning system, we experimentally validated the findings of the mathematical model using a minimized pheromone response pathway. This was constructed by refactoring the GPCR, Gα, Gβγ, and transcription factor so that these are expressed from constitutive promoters that we determined to give approximately the same expression of the native promoters in their uninduced state (Figures S2B–S2G). For the Gβγ component, we expressed both subunits together as a bicistronic protein (Ste4-2A-Ste18) where the self-cleaving 2A peptide releases equimolar amounts of the two proteins (Liu et al., 2017). The *FUS1* promoter was used to express sfGFP as the response reporter (Figure S2H), enabling pathway activation to be measured by flow cytometry. With this strain, we then individually varied the expression of the GPCR, Gα, and Gβγ by changing their promoter strengths while keeping all other components fixed. This gave *in vivo* results that qualitatively matched the model, demonstrating tunability of sensitivity by changing GPCR expression and revealing the predicted optimal levels of Gα and Gβγ that give the peak response (Figures 2D and 2E, experimental; Figure S3). As basal activity could be reduced by altering levels of either Gα or Gβγ, we decided to concentrate on Gα tuning, using a strain with fixed expression of Gβγ (yWS677) for all further experiments.

### Modulating Signal Output by Refactoring the Ste12 Transcription Factor

**Figure S4:**
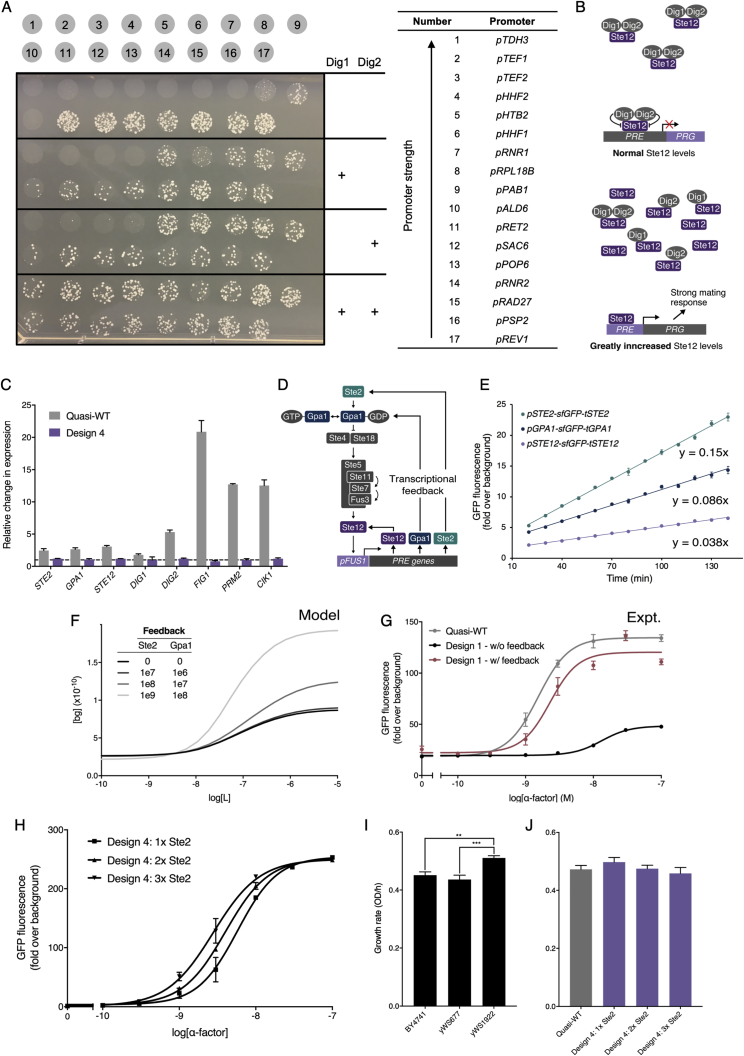
Redirecting the Pheromone Response to Synthetic Promoters, Related to Figure 3 (A+B) Experimental changes to the expression of Ste12. (A) A library of 17 constitutive promoters, from the YTK system, driving the expression of Ste12 with and without the overexpression of Dig1 and Dig2 in all combinations. The spotted yeast are direct transformants of two plasmids; the first containing the refactored pathway, with Ste12 under varying strengths of promoter, and the second containing either a blank spacer sequence, Dig1, Dig2, or Dig1 and Dig2 under the control of strong promoters. In the absence of Dig1 or Dig2 overexpression, expressing Ste12 with anything greater than a low-medium strength promoter was toxic to the cells. (B) It has previously been reported that Dig1 and Dig2 sit in a fine balance with Ste12, and the presence of these two negative regulators positively affect transcription by stabilizing inactive Ste12 (Houser et al., 2012). These data support these findings and suggests that increasing the concentration of Ste12 in the cells may lead to a large pool of unregulated transcription factor that may be constitutively activating the 100+ genes usually upregulated in the pheromone response, leading to cellular burden and toxicity (Dolan and Fields, 1990). As there is a fine balance between Ste12 and the two negative regulators, tuning the maximum signal output via the expression of Ste12 would require parallel tuning of both Dig1 and Dig2. Due to the combinatorial complexity of this problem we chose alternative approaches for modulating the signal amplitude and kept the expression Ste12 and synthetic transcription factors fixed at low levels using the *RAD27* promoter. (C) Decoupling the pheromone response pathway from PRE-containing genes using the LexA-PRD transcription factor targeting a synthetic promoter (*LexO(6X)-pLEU2m*). Fold-change in transcription of the refactored components, *STE2*, *GPA1* and *STE2/sTF*, the negative regulators of Ste12, *DIG1* and *DIG2*, and several of the most highly-upregulated genes in the pheromone response, FIG1, *PRM2*, *CIK1* (Su et al., 2010), in the Quasi-WT and Design 4 strains, with and without pheromone induction, as determined by RT-qPCR. The dotted line represents no fold-change. (D-G) Sensitivity of the Quasi-WT response is largely due to Ste12-mediated transcriptional feedback of Ste2, Gpa1, and Ste12. (D) Native transcriptional feedback of the Ste2, Gpa1, and Ste12 in the pheromone response pathway after stimulation. (E) Linear range within a time-course experiment measuring sfGFP fluorescence from *STE2*, *GPA1*, and *STE12* regulatory elements after stimulation with 100 nM ɑ-factor, fitted with a linear curve. The third minimized pathway (Figure 4C, Design 3) was used to upregulate native gene expression in response to ɑ-factor. (F) Model of Ste2 and Gpa1 feedback using different strengths of positive feedback on the expression of Ste2 and Gpa1, using values for Ste2 feedback 1 order of magnitude greater than Gpa1 as determined by the time-course experiments. (G) Reconstructing the Quasi-WT response in the first minimized pathway design by introducing transcriptional feedback of Ste2, Gpa1, and Ste12, controlled by the pheromone inducible *FUS1* promoter. The differences between Design 1 and Quasi-WT seems to be largely due to the positive feedback of native Ste12-activated promoters for Ste2, Gpa1, and Ste12. (H) Overexpression of Ste2 in the Design 4 strain with one or two additional integration vectors expressing Ste2 from a strong promoter (*pCCW12*). Although 2x and 3x Ste2 should be present in the system, the activation at 1e-10 M ɑ-factor remains unchanged. This also seems to be the limit for the Quasi-WT response, suggesting the receptor is at the physical limit of ɑ-factor detection and further Ste2 in the system will not be able to detect lower than this value. (I+J) Growth rates of the wild-type, model, and refactored strains. (I) Growth rates of the base strains, demonstrating no significant difference between BY4741 parental strain and the yWS677 strain. However, a significant increase in growth was seen in the yWS1922 strain compared to both BY4741 and yWS677. (J) Growth rates of the Quasi-WT and Design 4 Ste2 overexpression strains, demonstrating no significant difference between the four strains. Experimental measurements are sfGFP levels per cell determined by flow cytometry and shown as the mean ± standard deviation from triplicate isolates. Unless indicated, curves were fitted using GraphPad Prism variable slope (four parameter) nonlinear regression fit. GraphPad Prism one-way analysis of variance (ANOVA) used to determine statistical significance between growth rates (^∗^p < 0.05, ^∗∗^p < 0.005, ^∗∗∗^p < 0.0005).

Next, we sought to modulate maximum pathway output by varying the expression of the pheromone-responsive transcription factor, Ste12, as levels of transcription factor in a system often dictate gene expression strength (Brewster et al., 2014). However, increasing the expression of Ste12 led to poor cell growth, presumably by triggering high basal activity of mating response genes (Dolan and Fields, 1990) (Figures S4A and S4B). We therefore needed a new means to tune pathway-mediated expression without changing Ste12 concentrations.

**Figure 3:**
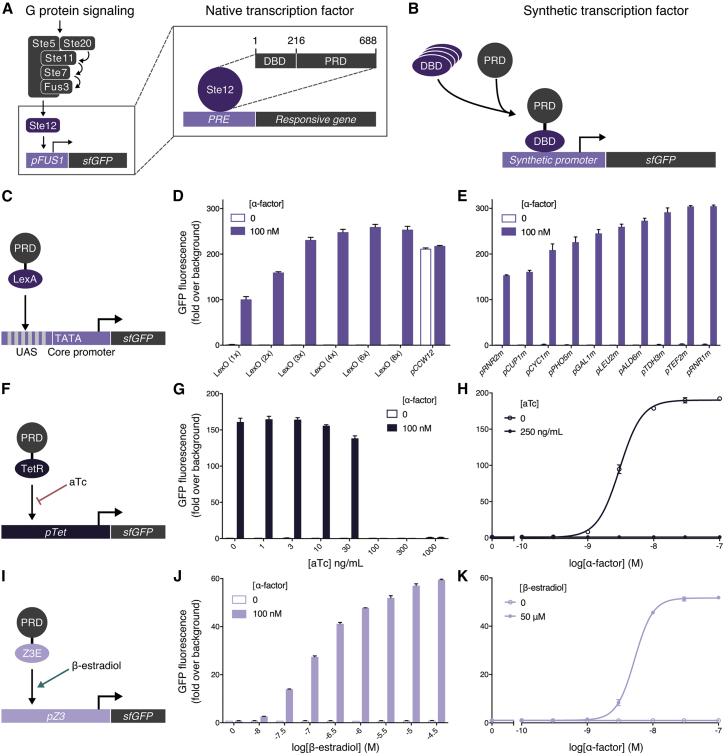
Modulating the Maximum Pathway Output Using Synthetic Transcription Factors (A) The native pheromone-responsive transcription factor, Ste12, composed of a DNA binding domain (DBD; 1-215) and pheromone-responsive domain (PRD; 216-688), targets a mating response gene via the pheromone-response element (PRE). (B) sTFs are created from fusion of orthogonal DBDs and the Ste12 PRD that can then be targeted to synthetic promoters. (C) Fusion of the full-length bacterial LexA repressor with the Ste12 PRD controls the expression of a modular promoter with an interchangeable UAS and core promoter region, upstream of sfGFP. (D and E) Maximum α-factor-activated pathway expression mediated by the LexA-PRD sTF driving the expression from synthetic promoter variants with UAS (D) and core promoter (E) modules modified, respectively. (F) Fusion of the TetR bacterial repressor with the Ste12 PRD targets a synthetic promoter with 7 repeats of the TetO binding site and the minimal *LEU2* promoter, driving aTc-repressible expression of sfGFP. (G) Inducing maximum α-factor-induced expression of the TetR-PRD-mediated signaling pathway over a range of aTc concentrations. (H) α-Factor dose-response curve of the TetR-PRD-mediated pathway with and without aTc. (I) A fusion of the PRD to the Z3E transcription factor (itself a fusion of Zif268 DBD and the human estrogen receptor ligand binding domain) targets the pZ3 promoter (a modified GAL1 promoter with six Zif268 binding sites) (McIsaac et al., 2014) driving β-estradiol-conditional expression of sfGFP. (J) Inducing maximum α-factor-induced expression of the Z3E-PRD-mediated signaling pathway over a range of β-estradiol concentrations. (K) α-Factor dose-response curve of the Z3E-PRD-mediated pathway with and without β-estradiol. Experimental measurements are sfGFP levels per cell determined by flow cytometry and shown as the mean ± SD from triplicate isolates. Curves were fitted using GraphPad Prism variable slope (four parameter) nonlinear regression fit. See also Figure S4.

By fusing the pheromone-responsive domain of Ste12 (PRD; 216-688) to the full-length LexA bacterial repressor protein (Mukherjee et al., 2015, Pi et al., 1997), we generated a synthetic transcription factor (sTF; LexA-PRD) able to target the pathway output to a library of modular synthetic promoters containing LexA operator sequences (LexO) (Figure 3C). We could then vary the recruitment of the LexA-PRD transcription factor by altering the number of operator sequences in the promoter upstream activating sequence (UAS), enabling us to modulate the maximum output of the response over a 3-fold range without compromising the tightness of the OFF state (Figure 3D). Changing the identity of the core region of the promoter to alter the transcription initiation rate offers a further approach to tuning the output (Figure 3E). Externally tuning the activation of the synthetic promoters with chemical inducers was also made possible by fusing the PRD to ligand-inducible DNA binding domains (DBDs) (McIsaac et al., 2014, Urlinger et al., 2000) (Figures 3F–3K). Finally, we validated that the pheromone response pathway and the downstream mating response were now decoupled by this design, by demonstrating that native Ste12-regulated genes were no longer transcriptionally activated by pathway activity (Figure S4C).

### Refactoring Enables Rational Optimization of Pheromone Sensing

**Figure 4:**
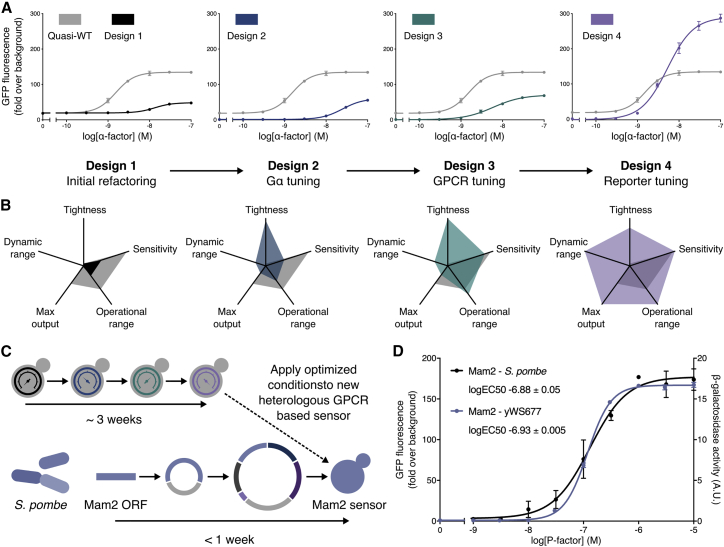
Tuning the Minimized Response Pathway through Iterative Refactoring (A) α-Factor dose-response curves for the 4 sequential minimized pathway designs compared to the Quasi-WT response. (B) Dose-response characteristics for the 4 minimized pathway designs compared to Quasi-WT. Tightness is defined as the reciprocal of basal activity and the dynamic range is defined as (maximum output/basal activity). Sensitivity and operational range were determined from the fitted curve, defining sensitivity as the lowest concentration for which a >2-fold change in GFP expression is seen, and operational range as the concentration span between the sensitivity and the lowest concentration that gives a GFP expression within 2-fold of the maximum. All values were then normalized to the minimum measurable value and the maximum calculated value in the dataset. (C) Domesticating the *S. pombe* Mam2 receptor in yWS677. The conditions identified during the 3-week optimization of the α-factor response with Ste2 receptor were directly applied to the design of the Mam2 sensor strain, enabling construction in less than a week. (D) P-factor dose-response curves of the Mam2 sensor (light blue) compared to the wild-type Mam2 response in its native *S. pombe* background (black) using previously obtained data from Croft et al. (2013). Slight differences in curve shape are likely due to differences in assay length and choice of reporter. Experimental measurements are sfGFP levels per cell determined by flow cytometry and shown as the mean ± SD from triplicate isolates. *S. pombe* Mam2 dose-response taken from Croft et al. (2013) and represents P-factor-dependent transcription of β-galactosidase using the sxa2 promoter, taking measurements 16 h after stimulation. Curves were fitted using GraphPad Prism variable slope (four parameter) nonlinear regression fit. See also Figure S5.

Together, our mathematical and *in vivo* models reveal that although GPCR dose-response is the output of a complex system, variability can be achieved by altering the promoter identify for just three components (receptor, Gα, and reporter), offering a simple approach to rationally tune the sensitivity, basal activity, and signal output of a GPCR signaling pathway. To demonstrate this in practice, we next set out to optimize the α-factor response of our minimized response pathway through iterative refactoring of these components. Our starting strain, with constitutive expression of the three components set at native levels (Figures 3B and 4A, design 1), performed poorly compared to the Quasi-WT strain, which we hypothesized was due to removal of regulated expression of pathway components. Indeed, Ste2, Gpa1, and Ste12 all have Ste12-activated promoters and so benefit from positive feedback in their native setting (Paliwal et al., 2007). Guided by our model, we were able to restore performance by engineered reintroduction of Ste12-mediated feedback (Figures S4D–S4G), however, we did not move forward with this design because it would couple sensitivity to pathway output.

Instead, to improve on the performance of the design 1 strain, we increased Gpa1 levels using the *PGK1* promoter to reduce the basal activity of the response to effectively zero (Figures 3B and 4A, design 2). The dose-response sensitivity could then also be boosted by increasing Ste2 expression via the strongest promoter available (*pCCW12*). This version (Figures 4A and 4B, design 3) now approached the sensitivity of the Quasi-WT strain for the α-factor inducer and the physical limits of the receptor (Figure S4H). Finally, the output strength of the response could be maximized by linking the pathway to activate the best performing synthetic promoter, *LexO*(*6x*)-*pLEU2m*, via the LexA-PRD sTF (Figures 4A and 4B, design 4). The resulting pathway was highly optimized in comparison to the Quasi-WT strain with improved operational range, tightness, dynamic range, and maximum output. Notably, this is achieved without the need for feedback regulation of signaling components and also offers a pathway decoupled from the >100 genes usually upregulated in the mating response (Roberts et al., 2000). Furthermore, the engineering we had performed to the design 4 strain had come with no penalty to the growth rate when compared to wild-type or Quasi-WT yeast (Figures S4I and S4J).

Understanding how the pathway dose-response can be shifted in this manner advances our basic knowledge of how component level changes effect signal transduction. Alongside this, it also offers direct applications for synthetic biology, where reprogramming cells to receive specific signals and respond in a desired manner is a core goal (Brophy and Voigt, 2014). Indeed, GPCRs represent the ideal sensory module for eukaryotic synthetic biology because they are responsive to a plethora of ligands and stimuli, often operate with high specificity (Heng et al., 2014), and naturally have modularity written into their signaling architecture (Andrianantoandro et al., 2006, Katritch et al., 2012).

**Figure S5:**
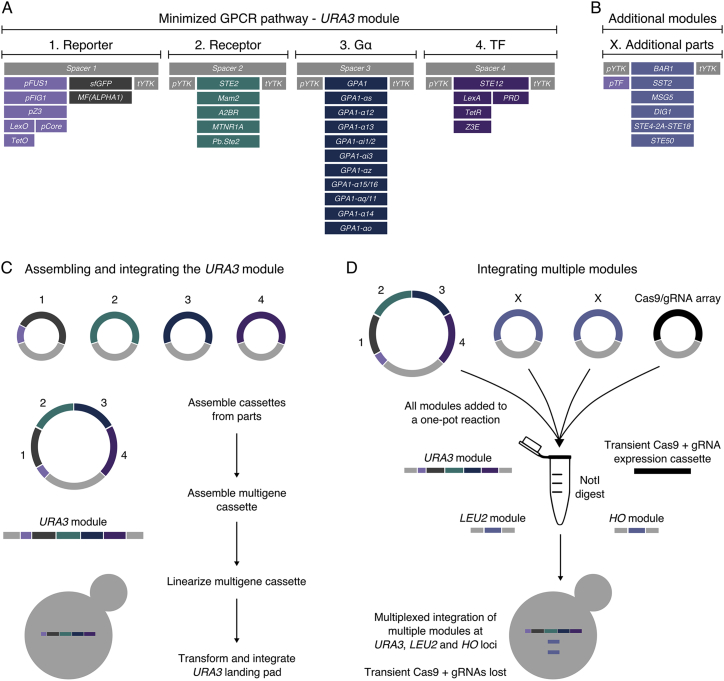
Final GPCR-Based Sensor Toolkit Format, Parts List, and Workflow, Related to Figure 4 Here, a module refers to either a cassette or multigene cassette that integrates into the yeast genome at one of the 3 sites provided in the YTK system starter set (*URA3*, *LEU2* or *HO* loci). All parts and cloning steps conform to the YTK MoClo standard (Lee et al., 2015). (A) A list of parts and formatting of the multigene cassette used for generating the minimized GPCR pathway (*URA3* module) which integrates at the *URA3* loci, indicating the instances where promoters (*pYTK*) and terminators (*tYTK*) from the YTK system are used. Spacer sequences are provided to exclude components in the instances where they are not required. Alternatively, components can be transferred to the additional *LEU2* or *HO* modules for combinatorial pathway refactoring, to reduce cloning requirements. (B) Additional parts for use with the *LEU2* and *HO* modules, for integrating at *LEU2* and *HO* loci, respectively. (C) Assembling and integrating the *URA3* module for generating a minimized GPCR sensor in the yWS677 model strain, following the YTK hierarchical assembly strategy (see Lee et al., 2015 for more details). (D) Multiple modules can be integrated simultaneously with the aid of CRISPR/Cas9-mediated DSB at the *URA3*, *LEU2* and *HO* landing pads. Once co-transformed with the other modules, the transient expression of Cas9 and appropriate gRNAs (no yeast marker or replicon) significantly increases the efficiency of double and triple plasmid integrations to practical levels (See Figures S1F and S1G).

With this in mind, we set out to establish our model strain as a host cell for rationally engineering yeast as sensors that detect diverse inputs via heterologous GPCRs (see Figure S5 for a description of the final toolkit). As an initial demonstration we took the Mam2 receptor from *Schizosaccharomyces pombe*, which detects a 23-amino acid peptide pheromone called P-factor (Ladds et al., 2005b). Using the optimized tuning levels determined for design 4, we generated a P-factor-sensing strain in less than a week. We then compared the response of this sensor strain to the response observed in its native context, as reported by Croft et al. (2013) (Figure 4D). The Mam2 sensor strain behaved almost exactly as in *S. pombe*, achieving an identical potency (EC_50_) to P-factor. Furthermore, the Mam2 sensor strain displayed no detectable basal activity and exhibited a 180-fold change in signal after activation, suggesting the optimization we had performed would also be suitable for other GPCRs.

### Engineered Consortia for Extending and Narrowing Operational Range

Efforts to create sensor strains over the last two decades have coupled heterologous GPCRs to the yeast pheromone response pathway with varying success. Now with the approach described here, the sensitivity, basal activity, and response output of cells sensing via heterologous GPCRs can be rationally tuned. However, one further important characteristic of a sensor—its operational range (the Hill slope of its dose-response curve)—is more difficult to adjust, as it is determined largely by the ligand-binding properties of the receptor. Some receptors will confer a narrow range switch-like behavior, only requiring a small increase in signal to trigger maximum output (i.e., a digital response), whereas others will give a wide operational range where there is a proportional relationship between signal and output (i.e., a linear response) (Dueber et al., 2007). For sensor applications, a linear response is typically required, whereas the digital response is more desirable for point-of-care and gene circuit applications.

**Figure S6:**
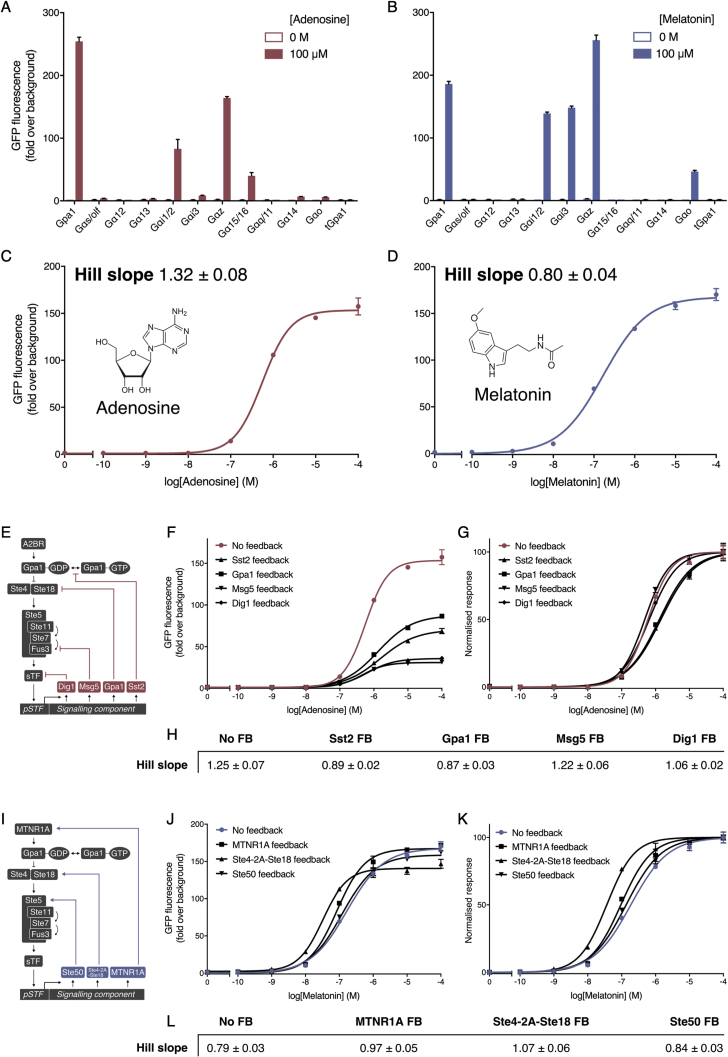
Linearizing and Digitizing the A2BR and MTNR1A Sensors Using Intracellular Feedback Loops, Related to Figure 5 (A-D) Screening the A2BR and MTNR1A receptors against a chimeric Gpa1-Gɑ library to identify optimal coupling to the pheromone response pathway. Sensors were created using the optimized conditions identified for Design 4, taking less than a week to create. (A) Coupling of the A2BR receptor to the Gpa1-Gɑ library in the presence and absence of saturating concentrations of adenosine (100 μM). (B) Coupling of the MTNR1A receptor to the chimeric Gpa1-Gɑ library in the presence and absence of saturating concentrations of melatonin (100 μM). As the wild-type Gpa1 coupled well to both A2BR and MTNR1A all future experiments were performed using this G protein. (C) Adenosine dose-response curve of the A2BR sensor strain, demonstrating a comparatively high Hill slope. (D) Melatonin dose-response curve of the MTNR1A sensor strain, demonstrating a comparatively low Hill slope. (E-L) Overlaying synthetic feedback (FB) loops onto the minimized sensing pathway to achieve linearization and digitization of A2BR and MTNR1A, respectively. (E) Negative feedback loops using the expression of Sst2, Gpa1, Msg5, and Dig1 as an output of pathway activation (Bardwell, 2004, Bashor et al., 2008, Galloway et al., 2013). (F+G) Feedback of negative regulators of the pheromone response pathway to linearize the dose-response of the A2BR receptor. (H) Hill slope values from the normalized curves of the 4 negative feedback conditions and no feedback control. Although feedback of the negative regulators had a significant impact on the response, when the output of each response was normalized, the effect on the Hill slope was minimal. (I) Positive feedback loops using the expression of Ste50, Ste4-2A-Ste18, and MTNR1A receptor as an output of pathway activation (Bardwell, 2004, Bashor et al., 2008, Galloway et al., 2013). Although Ste11 has been demonstrated as a viable candidate for positive feedback, it was omitted from the list as it was also shown to cause a large fitness defect when used in this manner (Ingolia and Murray, 2007). (J+K) Feedback of positive regulators of the pheromone response pathway to digitize the dose-response of the MTNR1A receptor. (L) Hill slope values from the normalized curved of the three positive feedback conditions and no feedback control. Feedback of these signaling components had a very small effect on the response of the system. Experimental measurements are sfGFP levels per cell determined by flow cytometry and shown as the mean ± standard deviation from triplicate isolates. Curves were fitted using GraphPad Prism variable slope (four parameter) nonlinear regression fit.

Given these considerations, we next set-out to solve how the operational range can also be tuned using an engineering approach, so that the Hill slope can be reduced to expand an operational range, or the Hill slope can be increased to narrow an operational range. For this we first built two new sensor strains, both sensing medically relevant metabolites by having human GPCRs coupled to our refactored yeast pathway. The chosen receptors were the adenosine-responsive A2BR receptor, previously shown to give a digital-like response in yeast (Beukers et al., 2004), and the melatonin-responsive MTNR1A receptor, previously shown to give a linear-like response in yeast (Kokkola et al., 1998) (Figures S6A–S6D).

While previous efforts have tuned the yeast pheromone response Hill slope by overlaying synthetic feedback loops into the MAPK cascade (Bashor et al., 2008, Galloway et al., 2013, Ingolia and Murray, 2007), feedback loops on the components tuned in our approach proved ineffective in changing the dose-response curves (Figures S6E–S6L), likely because our orthogonal sTFs do not have the autoregulatory feedback that Ste12 has via its native promoter (Paliwal et al., 2007). Without this avenue, we instead choose a different tactic, tuning the Hill slope by creating engineered communities of cells that change the average response at the population level.

**Figure 5:**
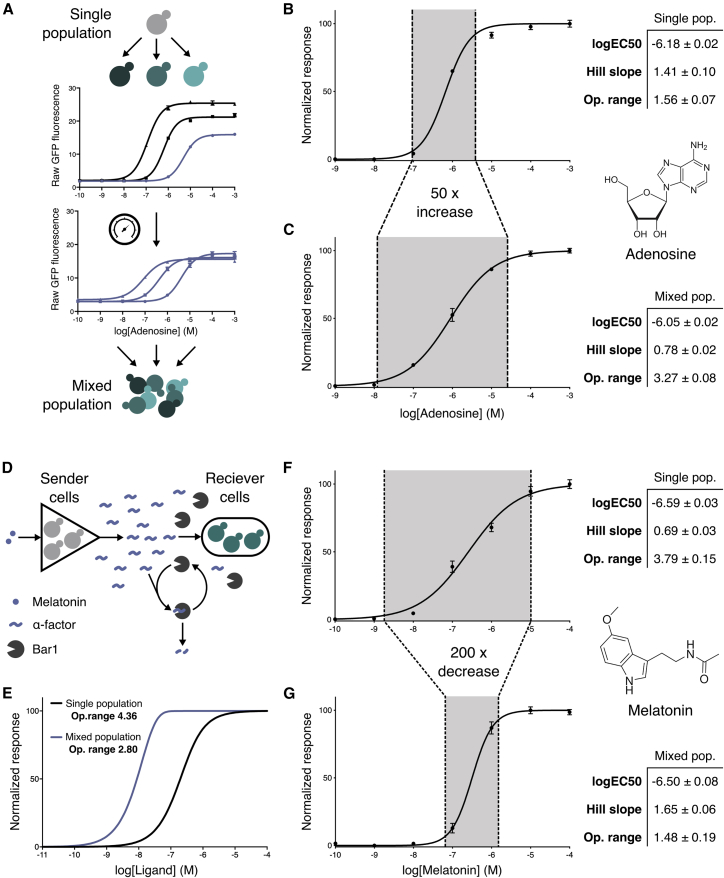
Engineered Consortia for Tuning the Operational Range of Heterologous GPCR Sensors (A) Engineered cells combined to produce a system with an extended operational range. First, a range of cells are produced with different sensitivities to a ligand by expressing the GPCR at different levels. Second, the ligand responses are tuned to produce equivalent maximum outputs. Third, the cells are combined in equal parts to create a mixed population of cells whose average expression has an extended operational range. (B) The dose-response of the human A2BR receptor to adenosine in a single yeast strain, operational over 1.6 orders of magnitude. (C) The extended dose-response of a consortia of three engineered strains, operational over 3.3 orders of magnitude. (D) A mixed population of yeast strains engineered as amplifier and reporters is designed to create a digital response from an otherwise linear sensor. In response to ligand, amplifier cells release α-factor that is detected by reporter cells constitutively secreting the α-factor degrading protease, Bar1. The presence of Bar1 degrades low levels of α-factor preventing reporter strain activation until levels of α-factor are high enough to saturate the capacity of Bar1-mediated degradation. (E) Computational model of the amplifier-reporter system response to increasing ligand (L) with Bar1-mediated threshold response included. (F) The broad dose-response of the human MTNR1A receptor to melatonin, operational over 3.8 orders of magnitude. (G) Digitized melatonin sensing with the two-strain system, operational over 1.5 orders of magnitude. Operational range is defined as the concentration span between 5% and 95% of the activated response. Experimental measurements are sfGFP levels determined by a plate reader and shown as the mean ± SD from triplicate isolates. Curves were fitted using GraphPad Prism variable slope (four parameter) nonlinear regression fit. See also Figures S6 and S7.

**Figure S7:**
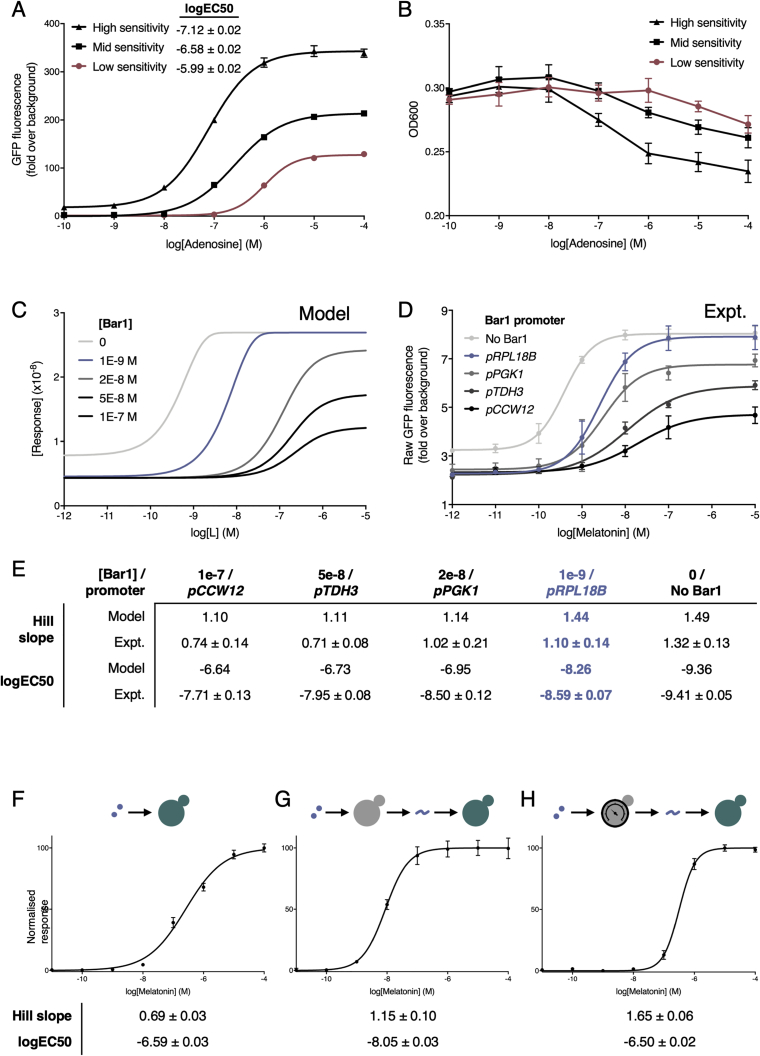
Linearizing and Digitizing the A2BR and MTNR1A Response, Respectively, Related to Figure 5 (A+B) Unnormalized sfGFP fluorescence is used to account for differences in growth rate between strains. (A) Adenosine dose-response of several adenosine sensors expressing A2BR using the weak *RPL18B* (low sensitivity), medium *HHF2* (mid sensitivity), and strong *CCW12* (high sensitivity) promoters. Experimental measurements are sfGFP levels per cell determined by flow cytometry and shown as the mean ± standard deviation from triplicate isolates. (B) Adenosine dose-dependent OD measurements of the three different sensitivity A2BR sensor strains after the standard 6 h assay time. To account for any differences in the growth rate between the different strains when activated or inactive, all future sfGFP measurements for experiments using microbial consortia were taken as the unnormalized fluorescence of the population using a plate reader. All strains set up at the starting OD of 0.175 at time 0 h and measurements were taken at 6 h. In this way, sfGFP fluorescence represents both growth rate and sfGFP production rate. (C-E) Tuning the expression of Bar1 in the two-cell amplifier-reporter system. (C) Varying the concentration of Bar1 in the two-cell amplifier-reporter model. (D) Experimentally varying the expression of Bar1 in the two-cell amplifier-reporter system using a select promoter library. (E) Hill slope values from computationally and experimentally varying the levels of Bar1 in the amplifier-reporter system. (F-H) Digitizing and fine-tuning the MTNR1A sensor response. (F) Melatonin dose-response of the MTNR1A sensor strain in a monogenic population of cells. (G) Digitization of the MTNR1A response via ɑ-factor mediated cell-cell communication. In response to melatonin, the first cell produces large quantities of ɑ-factor peptide that is then detected by the second cell, which responds by producing sfGFP. (H) Fine tuning the MTRN1A digital response by reducing the receptor expression in the first cell, so that the logEC_50_ matches the response of the single cell system. By lowering the expression of the MTNR1A receptor in the first cell using the *ALD6* promoter, we were able to shift the potency (logEC_50_) of the melatonin dose-response right by 1.5 orders of magnitude, to match the potency of the first, single cell system, while maintaining a high Hill slope. Data normalized to the minimum and maximum values within each dataset. Unnormalized, raw fluorescence readings were taken using a plate reader to account for growth during the 6h assay. Results are means ± standard deviation from triplicate isolates. Experimental measurements are sfGFP levels determined by a plate reader and shown as the mean ± standard deviation from triplicate isolates. Curves were fitted using GraphPad Prism variable slope (four parameter) nonlinear regression fit.

First, to create a population that linearizes the steep response curve of our adenosine-sensing cells, we took inspiration from a strategy employed by previous artificial sensor systems, where receptors with different sensitivities are combined and their average response determines the output (Vallée-Bélisle et al., 2012). We used rational tuning of GPCR levels to create two new strains with increased and decreased sensitivity to adenosine, and then tuned the output promoters so that their maximum outputs match (Figure 5A), using total fluorescence of each culture to measure GFP output (Figures S7A and S7B). We then co-cultured the three-sensing strains in a 1:1:1 ratio to create a consortium whose average response integrates the signal from all cells to give an extended operational range. This almost halved the Hill slope of the response while maintaining a similar potency, yielding an operational range 50-fold greater than the initial response (Figures 5B and 5C).

Narrowing the operational range of the melatonin-responsive MTNR1A sensor strains required more complex engineering as the Hill slope of a response can only be increased via mechanisms such as cooperativity (Dueber et al., 2007), sequestration (Buchler and Louis, 2008), or positive feedback (Ferrell, 2002). As before, we utilized a community-based approach, but here using cell-to-cell communication to enable feedback at the population level (Groß et al., 2011, Urrios et al., 2016). A two-cell system was designed where the first cell acts as an amplifier, sensing via MTNR1A and responding by secreting α-factor from the reintroduced gene. The second cell senses α-factor and responds with reporter gene (sfGFP) expression, and also secretes constitutive levels of the α-factor degrading protease, Bar1, to create a threshold for activation (Figures 5D and 5E).

Computational modeling revealed that fine-tuning Bar1 levels was important for a generating a steep Hill slope while maintaining tightness and high dynamic range, albeit with some loss of sensitivity (Figure S7C). To determine the optimal expression of Bar1, we tested a range of promoters and measured the dose-response of the two-cell system. The *RPL18B* promoter emerged as ideal choice for driving Bar1 expression as it eliminated basal activity in the two-cell amplifier-reporter system while also maintaining high levels of maximum signal (Figure S7D).

Following the creation of the α-factor-detecting reporter cells and the tuning of Bar1 levels secreted by these, we engineered the digital response in two steps using our toolkit (Figures S7F–S7H). Amplifier cells were first created by linking the output of MTNR1A sensor strain to the production of peptide pheromone, α-factor. The sensitivity of these cells was then tuned by adjusting MTNR1A receptor expression in the amplifier strain, so that the potency of the response in the two-cell system matched that of the single-cell version. When co-cultured in a 1:1 ratio, the final tuned two-cell system maintained the same potency but now provided a dose-response curve with a 2.3-fold increase in Hill slope and more than 200-fold decrease in the operational range (Figures 5F and 5G).

### Yeast GPCR Sensors for Metabolite Quantification and Pathogen Detection

The ability to rationally tune all dose-response properties for GPCR-signaling enables yeast strains to be optimized as sensors appropriate for different types of applications. To demonstrate this, we tackled two different example cases: metabolite quantification, where specificity and a wide dynamic range are desired, and point-of-care pathogen detection, where a sensitive digital-like response is needed.

**Figure 6:**
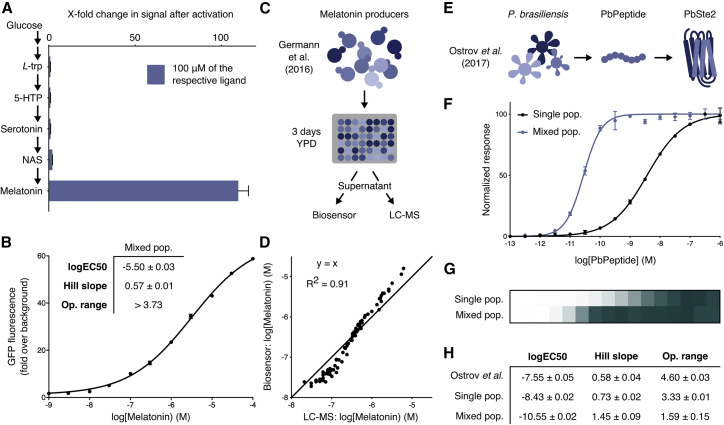
Applications of Tunable Yeast GPCR Sensor Strains (A) Selectivity of the MTNR1A sensor strain to melatonin and intermediates in the melatonin biosynthesis pathway from Germann et al. (2016). (B) A linearized MTNR1A sensor population consisting of two strains with different sensitives to linearize the dose-response of melatonin sensing in the range of concentrations appropriate for microbial production as reported by Germann et al. (C) Measuring the production of melatonin from the spent media of 88 different yeast producer strains using the MTNR1A sensor consortia and LC-MS. (D) The measured production of melatonin from the 88 producer strains, from Germann et al. (2016), as determined from measurements from the sensor consortia and LC-MS. A linear y = x curve was fitted to the dataset. (E) Detection of the *P. brasiliensis* pheromone peptide (PbPeptide) using the *P. brasiliensis* Ste2 homolog (PbSte2). (F and G) PbPeptide dose-response of the single cell PbSte2 sensor (F) compared to the two-cell amplifier-receiver consortia (G). (H) Potency (logEC_50_), Hill slope, and operational range values of the single and mixed cell populations compared to data from Ostrov et al. (2017). Experimental measurements are sfGFP levels per cell determined by flow cytometry (A–D) and GFP levels determined by a plate reader (E–H) and shown as the mean ± SD from triplicate isolates. Curves were fitted using GraphPad Prism variable slope (four parameter) nonlinear regression fit. See Table S1 for a list of GPCRs shown to functionally couple in *S. cerevisiae* that could be used for sensor applications.

Germann et al. (2016) recently reported production of melatonin from *S. cerevisiae* by constructing a biosynthetic pathway that converts *L*-tryptophan into melatonin via three non-native intermediates. We thus sought to create sensor strains optimized for measuring melatonin production from these engineered yeast, exploiting the fact that our MTNR1A sensor strains demonstrate exquisite specificity for melatonin over its precursors (Figure 6A).

To enable quantification of melatonin from our strains we engineered a two-strain consortia to widen the operational range to match the range of melatonin concentrations reported by Germann et al. (2016) from their production strains (Figure 6B). The resulting curve was linear over four orders of magnitude. We harvested supernatant from 88 different melatonin producer strains from the previous study, and in parallel, measured melatonin levels with our consortia of sensor strains and by liquid chromatography-mass spectrometry (LC-MS) (Figures 6C and 6D). The calculated concentrations of melatonin determined by the two methods agreed strongly, with the yeast sensor arguably more accurate over the large span of concentrations, due to the known limitations of the linear range in LC-MS measurement (Gika et al., 2014).

For pathogen detection, we next set out to tune a point-of-care sensor for *Paracoccidioides brasiliensis*, recently developed by Ostrov et al. (2017). This yeast GPCR-based sensor, utilizing the Ste2 homolog from *P. brasiliensis* (PbSte2), detects environmental levels of pheromone peptide released by haploid *P. brasiliensis* cells (PbPeptide) as a means of specifically detecting this human pathogen in complex samples (Figure 6E). This receptor exhibits a wide operational range with nanomolar sensitivity in *S. cerevisiae* (Ostrov et al., 2017), but a digital “yes/no” response would be more appropriate for point-of-care use, as would greater sensitivity. To optimize these two properties, we applied our amplifier-receiver two strain approach to the PbSte2 sensor (Figures 6F–6H). Increasing receptor levels with the *CCW12* promoter and incorporating signal amplification created a consortia that now detected in the picomolar range, reporting with a digital-like response due to an operational range now narrowed by 1,000-fold.

## Discussion

In this study, we used genome engineering and synthetic biology tools to refactor a minimized GPCR signaling pathway so that it could be rationally tuned both *in silico* and *in vivo*. This revealed three key design principles for tuning the signaling pathway dose-response curve: (1) sensitivity is increased by increasing GPCR numbers, (2) basal activity is reduced by finding the G protein expression optima, and (3) amplitude (pathway output) is tunable via synthetic transcription factors and engineered promoters. With these principles we were able to engineer yeast strains for desired performance as sensors for peptide inducers, and for primary and secondary metabolites.

Uncovering these principles was achieved here by the close connection between *in silico* modeling and experimental validation. This was made possible by refactoring yeast into a strain that effectively acts as an *in vivo* model, with component expression levels that can be individually varied and performance simple to measure via fluorescent output. The *in silico* model allowed us to first understand where component level tuning has the greatest impact on the signaling response, with experimental work then confirming this and identifying the promoters that will achieve these levels in subsequent engineering.

As well as confirming past observations on signaling stoichiometry, the model also revealed a complex, non-monotonic relationship between G protein subunit levels and pathway activity. An unexpected decrease in the response maximum output was predicted by the model for low Gα or high Gβγ levels, with this confirmed by experiments. By modeling different mechanisms, we were able to attribute this reduced activity to incorrect trafficking of Gβγ in the absence of free Gα. This insight told us that fine-tuned expression of G protein subunits would be essential for signal transduction that has minimal basal activity but maintains a high output when activated.

Varying signaling component stoichiometries in our *in silico* and *in vivo* work also demonstrated that the GPCR-mediated response is not merely defined by the receptor’s intrinsic properties (i.e., ligand affinity); instead, it is a function of the properties of all components in the signaling pathway and particularly their relative levels. This fact has important consequences. It explains how signal transduction behavior could be significantly altered by a change in component levels, whether due to a change in environmental conditions or due to altered expression and protein turnover, for example, in different tissues. Indeed, via this mechanism cells can have different sensitivities and activation thresholds for the same agonist while expressing identical receptors. Importantly, this fact also underlines why non-coding genetic variation, such as mutation in promoter regions, has to also be considered alongside protein polymorphisms when assessing how genetic variation links to health and to the efficacy of treatments (Ward and Kellis, 2012). Already, receptor variation in humans is recognized as a major cause of GPCR-targeting drugs being ineffective in many individuals (Hauser et al., 2018), and it is possible that non-coding mutations that alter pathway stoichiometries could further explain such cases.

We anticipate that the tuning principles uncovered here in yeast will also be relevant for GPCR signaling in all eukaryotes, however, it is worth re-stressing the large diversity in the type and structure of downstream signaling pathways paired with GPCRs in different organisms and cell types. The next steps for our approach will therefore be to use equivalent tools to refactor a canonical mammalian GPCR pathway so that its components can be tuned and assessed in isolation to the point where the dose-response to the agonist can be set as desired. This would also accelerate applications in pharmacology and healthcare that utilize GPCRs, such as in cell-based theranostics where cells are engineered to detect and act upon receiving defined cues within the human body (Heng et al., 2014).

While mammalian systems remain the go-to choice for studying GPCR activity, the genetic amenability, ease of use, and low cost make yeast an ideal organism for many sensor applications (Adeniran et al., 2015, Blount et al., 2012, Jarque et al., 2016, Ladds et al., 2005a). As demonstrated here, our model strain offers immediate applications for engineering yeast to sense its environment, whether as single strains or in engineered consortia. Already efforts in synthetic biology have used engineered yeast sensors as medical diagnostics (Adeniran et al., 2018), for pathogen detection (Ostrov et al., 2017), and as a tool for accelerating metabolic engineering (Ehrenworth et al., 2017, Mukherjee et al., 2015). In all these applications, it is desirable for the user to have control over the response to input and the magnitude of gene expression it triggers. Full ability to tune sensors, as shown here, allows engineering for desired detection windows, and could be used in further work to define thresholds for activation (e.g., for directed evolution) (Adeniran et al., 2018, Dong et al., 2010) or for matching the input-output levels when cells are engineered to detect and act or to communicate in connected systems (Billerbeck et al., 2018).

A current limitation of using yeast as sensors is that most medically relevant GPCRs do not port directly into *S. cerevisiae* without requiring optimization of expression, membrane translocation, and pathway coupling (Sarramegna et al., 2003). Co-expression of mammalian accessory proteins (Fukutani et al., 2015) and the humanization of the yeast membrane (Routledge et al., 2016) have also been shown to improve porting of receptors from mammalian species, and at the last count at least 50 different foreign GPCRs have been demonstrated to function in yeast (Table S1). These past successes provide a guide for those looking to generate yeast sensors for ligands and metabolites. However, without further experimental testing there are no guarantees that they will behave as required. In particular, the specificity of the receptor for the intended ligand is a major consideration as many receptors are promiscuous in what they bind. Another consideration is whether the ligand of interest can easily access a GPCR on the yeast cell membrane, especially given that yeast has a primitive cell wall. Fortunately for extracellular metabolite sensing, the porous structure of the cell wall is less of a concern, because it allows the free passage of molecules as large as 800 kDa (De Nobel and Barnett, 1991).

Our model strain now offers a new starting point for the many applications of GPCR-sensing enabling further work in a systematic, plug-and-play manner. Our overall strategy of simplifying and refactoring a natural pathway to first understand it and then rationally expand it should also be applicable to other systems in and beyond signal transduction. With the accelerating capabilities of genome engineering and synthetic biology in all organisms, it is likely that we will see the creation of equivalent *in vivo* model strains to rationally explore and exploit the key features and parameters of other important biological systems.

## STAR★Methods

### Key Resources Table

**Table:** 

REAGENT or RESOURCE	SOURCE	IDENTIFIER
**Bacterial and Virus Strains**		

NEB Turbo Competent *E. coli* (High Efficiency)	New England Biolabs	Cat# C2984I

**Chemicals, Peptides, and Recombinant Proteins**		

ɑ-factor (WHWLQLKPGQPMY)	GenScript	Custom synthesis (Purity > 95%)
P-factor (TYADFLRAYQSWNTFVNPDRPNL)	GenScript	Custom synthesis (Purity > 95%)
PbPeptide (WCTRPGQGC)	GenScript	Custom synthesis (Purity > 95%)
Adenosine	Sigma	Cat# A9215-5G
Melatonin	Sigma	Cat# M5250-1G
Anhydrotetracycline hydrochloride	Sigma	Cat# 37919-100MG-R
β-estradiol	Sigma	Cat# E8875-250MG

**Deposited Data**		

S288C reference genome	*Saccharomyces* Genome Database	S288C reference
Nanopore sequencing data of yWS677 genome	This paper	SRA accession: PRJNA516326

**Experimental Models: Organisms/Strains**		

BY4741 (*S. cerevisiae,* S288C derivative) MATɑ *his3Δ1 leu2Δ0 met15Δ0 ura3Δ0*	ATCC	ATCC: 4040002
yWS677 (*S. cerevisiae,* BY4741 derivative) *sst2Δ0 far1Δ0 bar1Δ0 ste2Δ0 ste12Δ0 gpa1Δ0 ste3Δ0 mf(alpha)1Δ0 mf(alpha)2Δ0 mfa1Δ0 mfa2Δ0 gpr1Δ0 gpa2Δ0*	This paper	N/A
yWS1922 (*S. cerevisiae,* yWS677 derivative) *ste4Δ0 ste18Δ0*	This paper	N/A
Quasi-WT (*S. cerevisiae,* yWS677 derivative) *STE2 GPA1 STE12*	This paper	N/A
*STE2* GFP (*S. cerevisiae,* yWS677 derivative) *pSTE2-sfGFP-tSTE2*	This paper	N/A
*GPA1* GFP (*S. cerevisiae,* yWS677 derivative) *pGPA1-sfGFP-tGPA1*	This paper	N/A
*STE12* GFP (*S. cerevisiae,* yWS677 derivative) *pSTE12-sfGFP-tSTE12*	This paper	N/A

**Oligonucleotides**		

For oligonucleotides used as primers and for creating gRNAs and Gpa1 C-terminal transplants used in this study, see Table S2.	This paper	N/A

**Recombinant DNA**		

Yeast MoClo Toolkit	Lee et al., 2015	Addgene Kit: 1000000061
Ellis Lab yeast CRISPR plasmids – pWS082	This paper	Addgene ID: 90516
Ellis Lab yeast CRISPR plasmids – pWS158	This paper	Addgene ID: 90517
Ellis Lab yeast CRISPR plasmids – pWS171	This paper	Addgene ID: 90518
Ellis Lab yeast CRISPR plasmids – pWS172	This paper	Addgene ID: 90519
For part plasmids created in this study see Table S3	This paper	Addgene ID: 123024-123065
For expression plasmids created in this study see Table S4	This paper	N/A

**Software and Algorithms**		

Prism	GraphPad	https://www.graphpad.com/scientific-software/prism/ RRID: SCR_002798
Benchling CRISPR design	Benchling	https://www.benchling.com RRID: SCR_013955
MATLAB	MathWorks	https://www.mathworks.com/products/matlab/ RRID: SCR_001622
Yeast GPCR Ternary Complex Model	This paper	BioModels ID: MODEL1901300001
Cell-To-Cell Digital Sensor Model	This paper	BioModels ID: MODEL1901300002

### Contact for Reagent and Resource Sharing

Further information and requests for resources and reagents should be directed to and will be fulfilled by the Lead Contact, Tom Ellis (t.ellis@imperial.ac.uk).

### Experimental Model and Subject Details

#### Bacterial Strains and Growth Media

NEB® Turbo Competent *E. coli* was used for all cloning experiments. Selection and growth of *E. coli* was performed in Lysogeny Broth (LB) medium at 37°C with aeration. With the exception of generating competent cells, the LB medium was supplemented with appropriate antibiotics (ampicillin 100 μg/mL, chloramphenicol 34 μg/mL, or kanamycin 50 μg/mL).

#### Yeast Strains and Growth Media

For a list of all yeast strains used in this study see Key Resources Table. Apart from BY4741, all strains used in this study are a derivative of the yWS677 model strain (*sst2Δ0 far1Δ0 bar1Δ0 ste2Δ0 ste12Δ0 gpa1Δ0 ste3Δ0 mf(alpha)1Δ0 mf(alpha)2Δ0 mfa1Δ0 mfa2Δ0 gpr1Δ0 gpa2Δ0*), which is itself a derivative of BY4741 (MATɑ *his3Δ1 leu2Δ0 met15Δ0 ura3Δ0*). The yWS677 model strain was generated using iterative markerless CRISPR/Cas9 genome engineering (see METHOD DETAILS). Further editing of yWS677 to create the additional strains was performed in a single step using markerless CRISPR/Cas9 genome engineering.

Yeast extract peptone dextrose (YPD) was used for culturing cells in preparation for transformation: 1% (w/v) Bacto Yeast Extract (Merck), 2% (w/v) Bacto Peptone (Merck), 2% glucose (VWR). Cells were cultured at 30°C shaking at 250 rpm.

Selection of yeast transformants was performed on synthetic complete (SC) dropout agar medium: 2% (w/v) glucose (VWR), 0.67% (w/v) Yeast Nitrogen Base without amino acids (Sigma), 0.14% (w/v) Yeast Synthetic Drop-out Medium Supplements without histidine, leucine, tryptophan, and uracil (Sigma) supplemented with 20 mg/L tryptophan (Sigma), and 20 g/L bacteriological agar (VWR). Depending on the required selection, SC dropout media was supplemented with 20 mg/L uracil (Sigma), 100 mg/L leucine (Sigma), and 20 mg/L histidine (Sigma). Cells were grown at 30°C static.

All liquid experiments were performed in synthetic complete (SC) medium with 2% (w/v) glucose (VWR), 0.67% (w/v) Yeast Nitrogen Base without amino acids (Sigma), 0.14% (w/v) Yeast Synthetic Drop-out Medium Supplements without histidine, leucine, tryptophan, and uracil (Sigma), 20 mg/L uracil (Sigma), 100 mg/L leucine (Sigma), 20 mg/L histidine (Sigma), and 20 mg/mL tryptophan (Sigma). Unless otherwise stated, all yeast strains were cultured in 500 μL of SC medium and grown in 2.2 mL 96 deep-well plates at 30°C in an Infors HT Multitron, shaking at 700 rpm.

### Method Details

#### Bacterial Transformations

Chemically competent cells were created following the TSS protocol for KCM transformations (Chung et al., 1989). A colony of *E. coli* was grown to saturation overnight in 10 mL of LB and then split into two 2 L baffled flasks with 500 mL of LB. The culture was grown for 2-3 h to OD600 ∼1.0, chilled on ice to stop growth, split between 50 mL conical tubes, and centrifuged at 4000 rpm at 4°C for 10 minutes. The supernatant was then discarded, and the cell pellets resuspended by aspiration in ice-cold TSS (85 mL LB, 10 g PEG-3350, 5 mL DMSO, and 2 mL 1 M MgCl_2_). 200 μL of the cell suspension was then aliquoted into PCR reaction tubes, flash frozen on dry ice, and put into a −80°C freezer for long term storage. To transform the DNA, 50 μL of 5 x KCM (500 mM KCl, 150 mM CaCl_2_, 250 mM MgCl_2_) was added to 200 μL of the competent cell prep after 10 minutes of thawing on ice. 50 μL of the competent cell-KCM cocktail was then added to DNA and transferred to a thermocycler with the following protocol: 4°C for 10 minutes, 42°C for 1 minute, 4°C for 1 minute, and then 30-60 minutes recovery at 37°C. Cells were then plated on solid LB medium supplemented with the appropriate antibiotics.

#### Yeast Transformations

Chemically competent yeast cells were created following the lithium acetate protocol (Gietz and Schiestl, 2007). Yeast colonies were grown to saturation overnight in YPD. The following morning the cells were diluted 1:100 in 15 mL of fresh YPD in a 50 mL conical tube and grown for 4-6 h to OD600 0.8-1.0. Cells were pelleted and washed once with 10 mL 0.1 M lithium acetate (LiOAc) (Sigma). Cells were then resuspended in 0.1 M LiOAc to a total volume of 100 μL/transformation. 100 μL of cell suspension was then distributed into 1.5 mL reaction tubes and pelleted. Cells were resuspended in 64 μL of DNA/salmon sperm DNA mixture (10 μL of boiled salmon sperm DNA (Invitrogen) + DNA + ddH_2_O), and then mixed with 294 μL of PEG/LiOAc mixture (260 μL 50% (w/v) PEG-3350 (Sigma) + 36 μL 1 M LiOAc). The yeast transformation mixture was then heat-shocked at 42°C for 40 mins, pelleted, resuspended in 200 μL 5 mM CaCl_2_ and plated onto the appropriate synthetic dropout medium.

#### Iterative Markerless Editing of Yeast Genome

All genomic edits were performed via CRISPR/Cas9, using a two-plasmid system, consisting of a gRNA expression plasmid (pWS082) and a CRISPR/Cas9 expression plasmid with the choice of three different selection markers (pWS158, URA3; pWS171, LEU2; pWS172, HIS3). This system supplies Cas9 and gRNAs on two different plasmids, which are first linearized and then gap repair with each other in yeast to create single plasmid which contains both components. For multiplexing edits, multiple gRNA fragments can be introduced into yeast simultaneously. Individual gRNAs were expressed using the format used in the YTK system (Lee et al., 2015), whereby a tRNA promoter drives the expression of a tRNA and HDV ribozyme fused to the 5′ of the gRNA followed by the *SNR52* terminator. Cas9 is then expressed using the *PGK1* promoter and terminator. To prepare the two plasmids for transformation, the CRISPR/Cas9 and gRNA expression plasmids were first digested with BsmBI or EcoRV, respectively, and the following size fragments were gel purified: pWS082, 1022 bp; pWS158, 10051 bp; pWS171, 10909 bp; pWS172, 10102 bp.

New gRNA targets were designed in Benchling (http://www.benchling.com) using the CRISPR tool. gRNA sequences were then created using two annealed 26 bp oligonucleotides and assembled into the gRNA expression vector using a BsmbI Golden Gate assembly using the (GACT) overhang at the 5′ and (GTTT) at the 3′. An additional (TT) was included between the 5′ overhang and gRNA sequence to complete the HDV ribozyme sequence:

5′ GACTTTNNNNNNNNNNNNNNNNNNNN 3′
3′ AANNNNNNNNNNNNNNNNNNNNCAAA 5′

Oligonucleotides were assembled into the pWS082 gRNA entry vector using the following protocol: Oligonucleotides were first resuspended at 100 μM in H_2_O. Each oligonucleotide was then treated separately with T4 polynucleotide kinase (PNK) in the following reaction: 1 μL oligonucleotide (100 μM), 1 μL 10 x T4 DNA ligase buffer (Promega), 0.5 μL T4 PNK (NEB), and 7.5 μL H_2_O. The mixture was then incubated at 37°C for 1 hour. The 10 μL reactions for both oligonucleotides in the fragment pair were then added together and brought to a total volume of 200 μL in H_2_O (10 μL oligo (sense) + 10 μL oligo (antisense) + 180 μL H_2_O). The oligonucleotides were then annealed under slowly decreasing temperatures using the following program: 96°C for 6 minutes followed by 0.1°C/s ramp down to 20°C, and then hold at 20°C. The resulting fragment was then ligated into the gRNA expression vector using Golden Gate assembly, which was prepared as follows: 0.1 μL of pWS082 (50 fmol/ μL), 1 μL of the small fragment, 1 μL T4 DNA ligase buffer (Promega), 0.5 μL T7 DNA Ligase (NEB), 0.5 μL BsmBI (NEB), and water to bring the final volume to 10 μL. Reaction mixtures were then incubated in a thermocycler using the following program: (42°C for 2 min, 16°C for 5 min) x 10 cycles, followed by a final digestion step of 60°C for 10 min, and then heat inactivation at 80°C for 10 min. The entire reaction mixture was then transformed directly into *E. coli* and plated on LB medium with ampicillin (100 μg/mL). For a list of oligonucleotides used to create the gRNA sequences in this study, see Table S2.

To edit the targeted regions, donor DNA was introduced into yeast alongside the CRISPR DNA to facilitate homology-directed repair at the double-strand break. Donor DNA was created by first cloning the sequence into the pYTK001 part entry vector. Donor DNA consisted of 500 bp arms of homology flanking a unique 24 bp sequence containing a new CRISPR/Cas9 target (landing pad), which were designed using the Benchling CRISPR tool to have an off-target score of 100 and a high on-target score. The plasmid was then sequence verified and primers were designed to amplify the donor DNA from the plasmid. The 1024 bp PCR amplicon was then gel purified to generate the donor DNA for transformation. For more information on donor DNA design see Figure S1A. For a list of landing pad sequences at the edited loci in the yWS677 and yWS1922 model strains, see below.

#### Landing Pads in the yWS677 and yWS1922 Model Strains

**Table:** 

*SST2* KO	TAACTTTGGAGGTGTTACTGTCGTACGTTCCTTCTAGGTTTTGCACGCACTATCTGAGGCGTTATAGGTTCAATT TGGTAATTAAAGATAGAGTTGTAAGAATGCAATCGTAGTCCACCT**CGG**TTAATTTCATTGAGAGTCTTACTCA TCTTCAGGTACAATTGCACAAACAGTCCTTTTTTTTTTCTTTAGTTCTCCTAACCTAATATGTCTTGATACCCATA
*FAR1* KO	TCAAAAAATTTCTATTTACTTTTATATTTCTTGACCATCCTTTACACAAAGTCTATAGATCCACTGGAAAGCTTC GTGGGCGTAAGAAGGCAATCTATTAGATCGTACTTAGAAATGAGG**CGG**TTAGTAGTTCGGGAATCGAGGCCC GTATTTCGAGGCTTTTGCTTTTCCTTTTTTTTTTTTCGTTTCTCCACGTCTATACTACGCAATGACTGAATATATAT
*BAR1* KO	CATGATGAATTCTTTAATGATCTTCGCGTGATTTAATTCTAGTGGTTCGTATCGCCTAAAATCATACCAAAATAA AAAGAGTGTCTAGAAGGGTCATATAAATGGGGTTAGCAAGTCGCA**CGG**TTAAGAAATCTGGAGTACAATTTTTT TATAGCATATAAATATCAAATATATAGTCATTTTTAATACATGGAAAGCATAATAAAAAAACAAGGGGAGTTTTA
*STE2* KO	TTCAAAGCAATACGATACCTTTTCTTTTCACCTGCTCTGGCTATAATTATAATTGGTTACTTAAAAATGCACCGTT AAGAACCATATCCAAGAATCAAAACTAGCTTTCGTGTTAGTACG**CGG**TTGATCAAAATTTACGGCTTTGAAAAAG TAATTTCGTGACCTTCGGTATAAGGTTACTACTAGATTCAGGTGCTCATCAGATGCACCACATTCTCTATAAA
*GPA1* KO	AGACCAAACTGAGTAGAAGCTATTCATACTGTAAATTGGTATTTTAGCATCACATCAATAATCCAGAGGTGTATA AATTGATATATTAAGGTAGGAAATATAGCATGGTGACACAAGCAG**CGG**TTGAAGGAACTGTATAATTAAAGTAG TGTTTAGATACGTAAATTCTGTTTCCGAAGATGCAAGAAGGAGCAGCAGCACCAGAAAAAATTACTATTTTTCTT
*STE12* KO	CTAGAGTGGATATTGATATTTCTCAAACAAGACTCGTCGAAGAAAACACACTTTTATAGCGGAACCGCTTTCTT TATTTGAATTGTCTTGTTCACCAAGGCATCGCTTCCTACTTCCGCT**CGG**TTGATATAATATAATTTTTGAATTTA TGATACAAGAATTAAAAATGCGGGCCAGAATTTAATATTAAACAATACTCAGAAGAAAACAACAAGGACAATCTG
*STE3* KO	TATTCATTTTGTGCAGTATTCACATATTCTATTTTATTGCTTTTTAACTTTAGAGGCAATTAAATTTGTGTAGGAA AGGCAAAATACTATCAAAATTTTCAATGTTTCTTGTCCAAGCGG**CGG**TTAACACAAGAGTGTCGCATTATATTT ACTGGACTAGGAGTATTTTATTTTTACAGGACTAGGATTGAAATACTGCTTTTTAGTGAATTGTGGCTCAAATA
*MF(ALPHA)1* KO	AAAATGTTACTGTTCTTACGATTCATTTACGATTCAAGAATAGTTCAAACAAGAAGATTACAAACTATCAATTT CATACACAATATAAACGATTAAAAGAACACGAGTTCCCAAAACCAG**CGG**TTAAGCCCGACTGATAACAACAGT GTAGATGTAACAAAGTCGACTTTGTTCCCACTGTACTTTTAGCTCGTACAAAATACAATATACTTTTCATTTCTCCG
*MF(ALPHA)2* KO	GTTTGAGGTGTCCTTCCTATATCTGTTTTTATATTCTATATAATGGATAATTACTACCATCACCTGCATCAAATT CCAGTAAATTCACATATTGGAGAAAGTTCCGATAGGCCAGCATAT**CGG**TTGAAAAATGACCCTAAACTACTTC TAAACCCTCTCGATTTCTTTCACGTTCATACAACACCTAGTTTTATTTATTTTCTTTTCAATCTGAGTAGTTGAGT
*MFA1* KO	TAGAGTCTTCATATATAAACCGCCAGAAATGAATTAATGAGAGGGATCTGTAACTGTTTCTCGGATAAAACCAAA ATAAGTACAAAGCCATCGAATAGAAGCAGTAACGCTCATCAGCTA**CGG**TTAGTTTCTGCGTACAAAAACGTTG TTCTCCCTCCTTTATCTTCCTTTTCCGCTACACCAATATATCATGTTTGTTCGTAATATTTCTTTTTAGACCTAAT
*MFA2* KO	TTTTATTTCCATCCACTTCTTCTGTCGTTCATCCGTTCATTGACATCACTAGAGACACCAGCGAGCTATCATCTT CATACAACAATAACTACCAACCTTACTTCTCCTGGAGATCAAGGA**CGG**TTAATTTTTGACGACAACCAAGAG GTCAAATCAATATCTACCCTTTCATTTATTACGTGTTGCTGGCAAACTAATTTATTCCAATTCTCTCATCATTAGCT
*GPR1* KO	TGTGTGTGTGTCTATAAAAAGCAGTAAGAGTCACCAAAAAAAAAAAACGACAAACAAGTGATCCGAAGTGTGACG AATAAAGCAAACTCTCCAACTCAAATCTAACCGTCGACTTTGGCG**CGG**TTAAAGTTTTTGTATCGCGATGTTTGAA AATGGAAAGTAAGGAACGTAATACAAATTGACAAGTAGCCGACATGAATGACGCTCACTTCTCTTATATATGT
*GPA2* KO	ACTACCCAAAGAGCAATCGATAGGTATAAAAGTGAGCAATTGCTATCACAGCGAGCCTTATTGTTACAGCACAAAT CACGCGTATTTTCAAGCAAATATCGCTGTTATCCTGCATCGGAA**CGG**TTGAATGCACAGCTAAAACAGAGACA AAACTGCATGCCTCTTCTCCCCTTTATTATCACCTTTAAAAAAGATAAAAAAAGAAACTGGAAAAAAGGTAAAAA
*URA3* LP	ATATATGTTAATTACCTTTTTTGCGAGGCATATTTATGGTGAAGGATAAGTTTTGACCATCAAAGAAGGTTAATGT GGCTGTGGTTTCAGGGTCCATAAAATATTATTGTACACCTACCG**CGG**TCCCGGGAATCTCGGTCGTAATGAT TTCTATAATGACGAAAAAAAAAAAATTGGAAAGAAAAAGCTTCATGGCCTTTATAAAAAGGAACTATCCAATACCT
*LEU2* LP	TATTTTATATTGACTTTCGTGTACATTGATCACATCGACTGTTCTATTGGCAAATGAACCACGGGCATTGACTAT TTTTCAGGTTACTACTATATATTATGCATCAGGTGGACTAGCATG**CGG**TCGACACGAAATTACAAAATGGAAT ATGTTCATAGGGTAGACGAAACTATATACGCAATCTACATACATTTATCAAGAAGGAGAAAAAGGAGGATGTAAAG
*HO* LP	CGCGATTCGGCCCAAATCAGTTTCTCACAGATCATTCGTAGAGTGAAAAAGCACATCGATTATTTGATACCCCT TTGGGTTAATTACTGTTGAGGTCTTTATGGACGAAATGCTTCACCA**CGG**TTTTAAATTGATGTATCTCATCGCAGG CACGGGCAGTACAGTGCCCTGAGCGTAGGGAAAAATGAAAAAAAGGATGTAACTTTTAACATAATTCCAGCACG
*STE4 KO*	ATTGGAAGGAAAGAGGGAAGAAAATACGATATTGCTAGTTCATTAAGTCAAGGAAGAAAATACTCAAAAAACT GTACAGCTCAATCAGGTACACATTACGGGACCGGCCTCGCGATGCCG**CGG**TTAGCTTCGAATTGGAAATACTGT GAGCAGTAATTATCAATGGATGCTATTATATAAATATACATACCTACACCCATCCCATATTTACATAGAATAACAAC
*STE18 KO*	GTTTGATGCAATATTTAACAAGGAGAACAGAAATGTTTTGTGACAGCACCTGTCAATTTTAGGATAGTAGCAATC GCAAACGTTCTCAATAATTCTAAGATTGCCATAGACGACTAGCCA**CGG**TTAATGATAGTAATAGAATCCAAAAA AAAAAAAAATATACATGCTTTTTCATATCCTCTCTCACCCTATCTTTTTTTTTCTTCTAATTTTGGCTCCGTTCATAT

Reagents for CRIPSR markerless genome editing were prepared as follows: 50 ng of linear CRISPR/Cas9 plasmid, 500 ng of each linearized gRNA expression plasmid, and 1000 ng of each donor DNA. The DNA was then transformed directly into yeast and plated on the appropriate selective media. Edits were validated by colony PCR followed by sanger sequencing of the amplified genomic region (for a list of primers used in this study, see Table S2). To rapidly iterate between successive edits when generating the model strain, yWS677, a marker cycling protocol was used, where two deletions or changes were performed per round of editing. Plasmid curing was skipped, and the marker for selecting the CRISPR/Cas9 plasmid was cycled between 3 markers (*URA3*, *LEU2*, and *HIS3*) for each iteration. The final edit was performed with CRISPR/Cas9 plasmid containing the *URA3* selection marker, which was counter selected using 5-FoA to cure the yeast of CRISPR machinery (Figures S1B and S1C). The absence of all CRISPR/Cas9 plasmids was validated by colony PCR and replica plating on selective media.

#### Nanopore Sequencing of the yWS677 Genome

DNA was isolated from yWS677 for Nanopore sequencing using the 100/G Genomic-Tip kit (QIAGEN), sheared to 20 kb using a g-TUBE (Covaris) and prepared for sequencing using a Ligation Sequencing Kit 1D^2^ R9.5 (Oxford Nanopore Technologies). The genomic DNA was then run on an R9.5 flow cell using a MinION Mk 1B (Oxford Nanopore Technologies). A standard 48h sequencing run was performed using the MinKnow 1.5.5 software using local basecalling. Reads were exported directly to fastq using MinKNOW. Canu (v1.5) was used to correct raw reads (http://www.canu.readthedocs.io) and smartdenovo (http://www.github.com/ruanjue/smartdenovo) was used to *de novo* assemble the reads into contiguous sequences (contigs) using default flags. Resulting contigs were compared to a WT reference genome (S288C, SGD) using lastdb/lastal (http://www.last.cbrc.jp) and viewed on integrative genome viewer (IGV) (http://www.software.broadinstitute.org) to inspect genomic changes. We then probed all discrepancies between the yWS677 genome and the S288C reference using a minimum alignment length of 100 bp.

#### Growth Curves

Single colonies were grown to saturation overnight in 3 mL YPD. The next day, the yeast cultures were back diluted to an OD_600_ of 0.175, and 200 μL was transferred to a 96-well clear, flat-bottom microplate (Corning). OD_600_ was the measured over 24 h by a Synergy HT Microplate Reader (BioTek) taking measurements every 15 minutes with shaking at 30°C in between readings. Growth rate per hour was calculated according to Equation 1, where *t* is time in hours.

(Equation 1) $$\left(ln\frac{OD600\left(t+3h\right)}{OD600\left(t\right)}\right)/3$$

#### Plasmid Construction

All plasmids within this study were created using the MoClo Yeast Toolkit (YTK) system (Lee et al., 2015). Additional sequences not included within the YTK system that were used within this study can be found in the Table S3. For a list of all plasmid constructs used in this study, see Table S4. Unless indicated, part sequences were either mutated or synthesized to remove or avoid all instances of the BsmBI, BsaI, BpiI, and NotI recognition sequences.

Construction of all plasmid constructs in Table S4 was achieved using Golden Gate assembly. All parts were set to equimolar concentrations of 50 fmol/μL (50 nM) prior to experiments. Golden Gate reactions were prepared as follows: 0.1 μL of backbone vector, 0.5 μL of each plasmid, 1 μL T4 DNA ligase buffer (Promega), 0.5 μL T7 DNA Ligase (NEB), 0.5 μL restriction enzyme (BsaI or BsmBI) (NEB), and water to bring the final volume to 10 μL. Reaction mixtures were then incubated in a thermocycler using the following program: (42°C for 2 min, 16°C for 5 min) x 25 cycles, followed by a final digestion step of 60°C for 10 min, and then heat inactivation at 80°C for 10 min. The entire reaction mixture was then transformed directly into *E. coli* and plated on LB medium with the appropriate antibiotics.

#### Creation of the Gɑ Variant Library

G protein C-terminal variants were created by substituting the GFP dropout cassette in the pWS936 Gpa1 C-terminal truncation vector for a small DNA fragment consisting of two annealed oligonucleotides. To create the small DNA fragment, oligonucleotides were first resuspended at 100 μM concentration in H_2_O. Each oligonucleotide was then treated separately with T4 polynucleotide kinase (PNK) in the following reaction: 1 μL oligonucleotide (100 μM), 1 μL 10 x T4 DNA ligase buffer (Promega), 0.5 μL T4 PNK (NEB), and 7.5 μL H_2_O. The mixture was then incubated at 37°C for 1 hour. The 10 μL reactions for both oligonucleotides in the fragment pair were then added together and brought to a total volume of 200 μL in H_2_O (10 μL oligo (sense) + 10 μL oligo (antisense) + 180 μL H_2_O). The oligonucleotides were then annealed under slowly decreasing temperatures using the following program: 96°C for 6 minutes followed by 0.1°C/s ramp down to 20°C, and then hold at 20°C. The resulting fragment was then ligated into the Gpa1 C-terminal truncation vector using Golden Gate assembly, which was prepared as follows: 0.1 μL of pWS936 (50 fmol/ μL), 1 μL of the small fragment, 1 μL T4 DNA ligase buffer (Promega), 0.5 μL T7 DNA Ligase (NEB), 0.5 μL BsmBI (NEB), and water to bring the final volume to 10 μL. Reaction mixtures were then incubated in a thermocycler using the following program: (42°C for 2 min, 16°C for 5 min) x 10 cycles, followed by a final digestion step of 60°C for 10 min, and then heat inactivation at 80°C for 10 min. The entire reaction mixture was then transformed directly into *E. coli* and plated on LB medium with chloramphenicol (34 μg/mL) For a list of oligonucleotides used to create the G protein library, see Table S2.

#### Multiplexed Yeast Plasmid Integrations

The yWS677 model strain was prepared for multiplex integration of selectable plasmids by integrating landing pads (LPs) at the *URA3*, *LEU2*, and *HO* loci, conforming to the YTK integration plasmid format. Transient expression of Cas9 and the gRNAs targeting the landing pads was achieved by individually assembling Cas9 (*pTDH3-Cas9-tTDH1*) and the gRNAs (created using the YTK050 sgRNA dropout) into cassette plasmids without a yeast marker or yeast replicon. Double and triple integration of marker plasmids were performed with 100 and 200 ng of plasmid, respectively, with 100 ng of the Cas9 expression cassette and 200 ng of each gRNA expression cassette. All plasmids were first linearized by digestion before transformation using NotI-HF (NEB). Successful plasmid integration was selected for using synthetic drop-out media missing the appropriate supplements. Cas9 and gRNA expression was transient and quickly lost due to lack of selection or flanking homology to genome. Initially validated by colony PCR and then assumed thereafter.

#### RT-qPCR

All quantitative PCR (qPCR) reactions were performed in an MasterCycler ep realplex 4 (Eppendorf) using SYBR FAST Universal qPCR Master Mix (Kapa Biosystems). For RNA purification, RNA was isolated from yeast culture grown to an OD600 of 1 ± 0.1 using a YeaStar RNA Kit (Zymo Research). RNA was quantified by nanodrop spectrophotometer (Thermo Fisher) and cDNA was generated from each RNA prep using a High Capacity cDNA Reverse Transcription Kit (Applied Biosystems). Each qPCR reaction contained 20 ng of cDNA. qPCR results were normalized to the housekeeping gene *HTB2*. All qPCR primers were designed manually using Benchling.

#### Ligand Sensing Protocol

All sensor strains were picked into 500 μL of synthetic complete media and grown in 2.2 mL 96 deep-well plates at 30°C in an Infors HT Multitron, shaking at 700 rpm overnight. The next day, saturated strains were then diluted 1:100 into fresh media. After 2 h of incubation the strains were induced with their respective ligands and incubated for a further 4 h. All ligands were dissolved in DMSO, and the final concentration of DMSO in all cultures was 1 %. For strains using the TetR-PRD or Z3E-PRD transcription factor, aTc and β-estradiol was added during the back dilution at time 0 h. To perform flow cytometry and plate reader measurements, 200 μL from each well was directly transferred to a 96-well clear, flat-bottom microplate (Corning).

For monoclonal cell experiments, cell fluorescence was measured by an Attune NxT Flow Cytometer (Thermo Scientific) with the following settings for measuring sfGFP: FSC 300 V, SSC 350 V, BL1 500 V. Fluorescence data was collected from 10,000 cells for each experiment and analyzed using FlowJo software. For polyclonal cell experiments, cell fluorescence was measured by a Synergy HT Microplate Reader (BioTek) with the following settings for measuring sfGFP: excitation 485/20, emission 528/20, gain 80. Unnormalized, raw fluorescence readings from each well were used for data analysis.

#### Detection of Microbially Produced Melatonin

Samples for mass spectrometry and the MTNR1A sensor were prepared by centrifuging yeast cultures at 4000 rpm in a large desktop centrifuge for 10 minutes at 4°C and extracting the supernatant. Supernatant samples were kept on ice before running on the LC-MS or transferred to the −20°C for later use. No further sample preparation was performed on the supernatant sample before running on the LC-MS. Melatonin standards were kept in 100% DMSO before being diluted in spent media. Spent media was prepared from BY4741 in the same manner as the measured yeast.

An LC-MS method was developed for the measurement of melatonin in media, using an Agilent 1290 UPLC and 6550 quadrupole – time-of-flight (Q-ToF) mass spectrometer with electrospray ionization (Santa Clara, CA). The UPLC column was an Agilent Zorbax Eclipse Plus C-18, 2.1 × 50mm and 1.8um particle size. The UPLC buffers were 0.1% formic acid in water and 0.1% formic acid in acetonitrile (v/v). The gradient elution method is detailed below.

#### The LC Gradient Elution Method for the Measurement of Melatonin in Media

**Table:** 

Time (minutes)	% Solvent A	% Solvent B	Flow rate (mL/min)
0	100	0	0.5
0.5	100	0	0.5
1.5	70	30	0.5
2	5	95	0.5
2.5	5	95	0.5
2.6	100	0	0.5
3.6	100	0	0.5

Quantitation was based on the LC retention time from melatonin standard solutions and the area of accurately measured diagnostic ions from the molecule, namely the protonated molecule, [M+H]^+^, along with an in-source fragment (see below). The solutions of a melatonin standard in media were used to generate calibration curves.

#### The MS Ions Used for the Measurement of Melatonin

**Table:** 

	Quantifier ion [M+H]+	Qualifier ion [M+H-C_2_H_5_NO]
Melatonin (C_13_H_16_N_2_O_2_)	233.1285	174.0913

To measure the melatonin from producer strains using the MTNR1A sensor strains, 50 μL of supernatant was added to an adjusted 450 μL volume of the sensor cells and run on the flow cytometer according to the ligand sensing protocol. Melatonin concentrations were then calculated from a standard curve.

#### Dose-Response Fitting

All presented dose-response fittings were generated in Prism 7 (GraphPad), which was used to determine the logEC_50_ and Hill slope. To determine all remaining properties of the dose-response curve, curve fitting was performed using Python (SciPy and Matplotlib) using the 4PL model (Equation 2), where x is the concentration, A is the minimum asymptote, B is the steepness, C is the inflection point and D is the maximum asymptote.

(Equation 2) $$f\left(x\right)=\frac{A-D}{1+{\left(\frac{x}{C}\right)}^{B}}+D$$

#### Computational Modeling

To model biochemical reactions systems, it is customary to use a set of ordinary differential equations (ODEs) to describe changes in concentrations of biochemical species. The systems of equations will be defined as shown in Equation 3.

$$\frac{dx}{dt}=f_{x}\left(x,\;y,\ldots ,t\right)$$

$$\frac{dy}{dt}=f_{y}\left(x,\;y,\ldots ,t\right)$$

(Equation 3) …

Where in this case, x,y,… are the concentrations of molecular species within the system, and $f_{x},\;f_{y},\ldots$ are functions describing the molecular interactions.

Numerical integration of the system of ODEs may allow us to derive the concentration of all molecular species at a certain time point. Simulations from numerical integration were performed using MATLAB R2017a offered by MathWorks.

#### GPCR Ternary Complex Model

##### Rationale and Implementation

The aim to formulate a system of ODEs of the Ste2, Gpa1, and Ste4:Ste18 promoter library required a top-down approach via reproduction of similar trends against general experimental results. In terms of the Ste2 library, an increase in receptor concentration directly increases maximum response while retaining low constitutive signaling. An increase in [Gpa1] or [G_ɑ_] in the pathway should demonstrate an optimal response, where a specific concentration of [G_ɑ_] produces the maximum response while having constitutive signaling. Increases in [Ste4:Ste18] or [G_βγ_] should also demonstrate an optimal response with some basal activity. Thus, it is important to design a detailed system of ODEs which differentiates the ligand-induced responses against the constitutive response.

Moreover, since the refactored strain observe changes in response by modifying the receptor (Ste2), G_ɑ_ subunit (Gpa1), and G_βγ_ subunit (Ste4:Ste18) concentration, a detailed model focusing on receptor/G protein interactions was used. Although there are models presenting full yeast pheromone ODE models in detail (Kofahl and Klipp, 2004, Shao et al., 2006), these models are not be ideal for demonstrating the intricate changes in multiple constitutive activities and normal responses within the receptor/G protein interactions. Thus, we have chosen as our basis of the mathematical model our previously described cubic ternary complex model (Bridge et al., 2018), which focuses upon the receptor and heterotrimeric G protein complex. In contrast to our previous work, our model output is the changes in free G_βγ_ subunits since this is how the *S. cerevisiae* pheromone-response pathway mediates downstream signaling (Whiteway et al., 1989). Upon release of the G_βγ_ dimer from G_ɑ_ downstream signaling continues through interaction with Ste5 and recruitment of a MAPK cascade (Ste11, Ste7 and Fus3 respectively). It has been reported that the MAPK-cascade does not modify the Ste2/G protein dynamics and simply acts to amplify and transduce the response from the plasma membrane to the nucleus (Yi et al., 2003). As such, given our specific interest in the dynamics of the R/G protein and the fact that our refactored yeast cells are designed to be linearly correlated we have not included the MAPK in our model simulations with the exception of Ste5, which is considered as the downstream effector.

##### Model Formulation

Here, we formulate the ODE model dynamics, as we have described previously (Bridge et al., 2018, Croft et al., 2013, Prokic et al., 2015, Smith et al., 2009) for refactored *S. cerevisiae* strain yWS1922 to recapitulate results obtained through varying expressions of Ste2 or Gpa1 through a promoter library (Figures 2D and 2E, Experimental). The basis of the mathematical model used is from a cubic ternary complex model (Bridge et al., 2018), in which a receptor may be under its inactive conformation *R* or its active conformation *R^∗^.* Furthermore, a receptor may bind to G protein *G,* and activation of receptor may cause *G* to dissociate and undergo the G protein cycle. The model describes ligand binding, receptor activation, G protein binding and the G protein cycle (Bridge et al., 2018). The scheme used for the reaction model is depicted in Methods S1A with detailed ODEs in Equation 4. The synthesis and degradation of the models were assumed to be slower compared to the signaling events and thus omitted for simplicity. Taking ligand concentration as a constant, our system consists of 8 receptor states and 8 non-receptor bound G protein states, with a total for the system of 16 ODEs. For model outputs, concentrations [*G*_*βγ*_*^∗^STE5*] will be taken as subsequent downstream pathways are designed to be linearly correlated in the system. For the individual kinetic rate constants, the lowercase k was used, along with subscripts + and – to denote forward and backward reactions.

$$\frac{d\left[R\right]}{dt}=k_{L-}\left[LR\right]-k_{L+}\left[L\right]\left[R\right]+k_{act-}\left[R^{*}\right]-k_{act+}\left[R\right]+k_{G-}\left[RG\right]-k_{G+}\left[R\right]\left[G\right]$$

$$\frac{d\left[LR\right]}{dt}=\;k_{L+}\left[L\right]\left[R\right]-k_{L-}\left[LR\right]+\zeta_{-}k_{act-}\left[LR^{*}\right]-\zeta_{+}k_{act+}\left[LR\right]+\nu_{-}k_{G-}\left[LRG\right]-\nu_{+}k_{G+}\left[LR\right]\left[G\right]$$

$$\frac{d\left[R^{*}\right]}{dt}=k_{act+}\left[R\right]-k_{act-}\left[R^{*}\right]+\zeta_{-}k_{L-}\left[LR^{*}\right]-\zeta_{+}k_{L+}\left[L\right]\left[R^{*}\right]+\mu_{-}k_{G-}\left[R^{*}G\right]-\mu_{+}k_{G+}\left[R^{*}\right]+k_{GTP+}\left[R^{*}G\right]$$

$$\frac{d\left[LR^{*}\right]}{dt}=\zeta_{+}k_{act+}\left[LR\right]-\zeta_{-}k_{act-}\left[LR^{*}\right]-\;\zeta_{-}k_{L-}\left[L\right]\left[R^{*}\right]-\zeta_{-}k_{L-}\left[LR^{*}\right]+\mu_{-}\nu_{-}k_{G-}\left[LR^{*}\right]-\mu_{+}\nu_{+}k_{G+}\left[LR^{*}\right]\left[G\right]+\nu_{-}k_{GTP+}\left[LR^{*}G\right]$$

$$\frac{d\left[RG\right]}{dt}=k_{G+}\left[R\right]\left[G\right]-k_{G-}\left[RG\right]+\nu_{-}k_{L-}\left[LRG\right]-\nu_{+}k_{L+}\left[L\right]\left[RG\right]+\mu_{-}k_{act-}\left[R^{*}G\right]-\mu_{+}k_{act+}\left[RG\right]$$

$$\frac{d\left[LRG\right]}{dt}=\nu_{+}k_{G+}\left[LR\right]\left[G\right]-\nu_{-}k_{L-}\left[LRG\right]+\nu_{+}k_{L+}\left[L\right]\left[RG\right]-\nu_{-}k_{L-}\left[LRG\right]+\mu_{-}\zeta_{-}k_{act-}\left[LR^{*}G\right]-\mu_{+}\zeta_{+}k_{act+}\left[LRG\right]$$

$$\frac{d\left[R^{*}G\right]}{dt}=\mu_{+}k_{G+}\left[R^{*}\right]\left[G\right]-\mu_{-}k_{G-}\left[R^{*}G\right]+\mu_{+}k_{act+}\left[RG\right]-\mu_{-}k_{act-}\left[R^{*}G\right]-k_{GTP+}\left[R^{*}G\right]$$

$$\frac{d\left[LR^{*}G\right]}{dt}=\mu_{+}\nu_{+}k_{G+}\left[LR^{*}\right]\left[G\right]-\mu_{-}\nu_{-}k_{G-}\left[LR^{*}\right]+\zeta_{+}\nu_{+}k_{L+}\left[L\right]\left[R^{*}G\right]-\zeta_{-}\nu_{-}k_{L-}\left[LR^{*}G\right]+\mu_{+}\zeta_{+}k_{act+}\left[LRG\right]-\mu_{-}\zeta_{-}k_{act-}\left[LR^{*}G\right]-\nu_{-}k_{GTP+}\left[LR^{*}G\right]$$

$$\frac{d\left[G\right]}{dt}=k_{G-}\left[RG\right]-k_{G+}\left[R\right]\left[G\right]+\nu_{-}k_{L-}\left[LRG\right]-\nu_{+}k_{G+}\left[LR\right]\left[G\right]+k_{GRA+}\left[\alpha_{GDP}\right]\left[\beta \gamma \right]+k_{GRA+}\left[\alpha_{GDP}\right]\left[\beta \gamma^{*}\right]-k_{GRA-}\left[G\right]+\mu_{-}k_{G-}\left[R^{*}G\right]-\mu_{+}k_{G+}\left[R^{*}\right]\left[G\right]+\mu_{-}\nu_{-}k_{G-}\left[LR^{*}\right]\;-\mu_{+}\nu_{+}k_{G+}\left[LR^{*}\right]\left[G\right]$$

$$\frac{d\left[\alpha_{GDP}\right]}{dt}=k_{hyd+}\left[\alpha_{GTP}\right]-k_{hyd-}\left[\alpha_{GDP}\right]+k_{GRA-}\left[G\right]-k_{GRA+}\left[\alpha_{GDP}\right]\left[\beta \gamma \right]-k_{GRA+}\left[\alpha_{GDP}\right]\left[\beta \gamma^{*}\right]$$

$$\frac{d\left[\alpha_{GTP}\right]}{dt}=k_{hyd-}\left[\alpha_{GDP}\right]\;-k_{hyd+}\left[\alpha_{GTP}\right]+k_{GTP+}\left[R^{*}G\right]+\nu_{-}k_{GTP+}\left[LR^{*}G\right]$$

$$\frac{d\left[\beta \gamma \right]}{dt}=-k_{GRA+}\left[\alpha_{GDP}\right]-k_{conact}\left[\beta \gamma \right]+k_{rev}\left[\beta \gamma^{*}\right]-k_{E+2}\left[STE5\right]\left[\beta \gamma^{*}\right]+k_{E=2}\left[\beta \gamma^{*}STE5\right]$$

$$\frac{d\left[\beta \gamma^{*}\right]}{dt}=k_{GRA-}\left[G\right]+k_{GTP+}\left[R^{*}G\right]+\nu_{-}k_{GTP+}\left[LR^{*}G\right]+k_{conact}\left[\beta \gamma \right]-k_{rev}\left[\beta \gamma^{*}\right]-k_{GRA+}\left[\alpha_{GDP}\right]\left[\beta \gamma^{*}\right]-k_{E+1}\left[STE5\right]\left[\beta \gamma^{*}\right]+k_{E=1}\left[\beta \gamma^{*}STE5\right]$$

$$\frac{d\left[STE5\right]}{dt}=k_{E-1}\left[\beta \gamma^{*}STE5\right]-k_{E+1}\left[STE5\right]\left[\beta \gamma^{*}\right]-k_{E+2}\left[STE5\right]\left[\beta \gamma \right]+k_{E-2}\left[\beta \gamma STE5\right]$$

$$\frac{d\left[\beta \gamma^{*}STE5\right]}{dt}=k_{E+1}\left[STE5\right]\left[\beta \gamma^{*}\right]-k_{E-2}\left[\beta \gamma STE5\right]$$

(Equation 4) $$\frac{d\left[\beta \gamma STE5\right]}{dt}=k_{E+2}\left[STE5\right]\left[\beta \gamma \right]-k_{E=2}\left[\beta \gamma STE5\right]$$

#### Simulation Results

Here we present the numerical results used to illustrate the Ste2 and Gpa1 promoter library model. Both concentration-response curves are created through MATLAB R2017a using the pre-installed ODE solver ode15s. ODEs were solved to obtain endpoint concentration of [*G*_*βγ*_*^∗^*] after 1000 time points (Figures 2D and 2E, Model). Prior to the addition of ligand, the system was run for 1e^8^ time points to enable complex intermediates to obtain equilibrium (Bridge et al., 2018, Croft et al., 2013, Prokic et al., 2015, Smith et al., 2009). The simulated results demonstrate similar trends to the ‘wet’ experimental data (Figures 2D and 2E, Experimental – Ste2), where increases in Ste2 (*R* in the model) concentration shows an increase in maximum response. Conversely, for a fixed concentration of *R* increasing concentrations of Gpa1 (*G* in the model) demonstrates a non-monotonic relationship between G protein concentration and maximal signaling response (Figures 2D and 2E, Experimental – Gpa1). For fixed concentrations of *R* and *G*_*ɑ*_ while increasing Ste4/Ste18 (*G*_*βγ*_ in the model), a non-monotonic relationship shows an optimal signaling response (Figures 2D and 2E, Experimental – Ste4:Ste18). The base parameter set, including initial species concentrations used for both simulations can be found below. Parameter values were qualitatively fit through experimental dose-response results of the Gpa1 and Ste2 promoter library, retaining source values when possible.

#### Parameter Values Used for Constructing the Computational Gpa1 and Ste2 Promoter Library

**Table:** 

Parameter	Meaning	Values	Units	Source
k_L+_	Ligand binding rate	4E+7	M^-1^s^-1^	Fitted
k_L-_	Ligand unbinding rate	3.10E-01	s^-1^	(Bridge et al., 2010)
k_act+_	Receptor activation rate to R^∗^	1.00E+00	s^-1^	“
k_act-_	Receptor deactivation rate from R^∗^	1.00E+03	s^-1^	“
k_G+_	G protein binding rate	1.00E+08	M^-1^s^-1^	“
k_G-_	G protein unbinding rate	1.00E-01	s^-1^	“
k_GRA+_	G protein re-association rate	7.00E+08	M^-1^s^-1^	Fitted
k_GRA-_	G protein dissociation rate	1.30E-03	s^-1^	(Bridge et al., 2010)
k_hyd+_	Hydrolysis rate of G_αGTP_	1.00E-04	s^-1^	“
k_hyd-_	Exchange rate of GTP to GDP at G_α_	1.00E-04	s^-1^	“
k_GTP+_	R^∗^G dissociation rate	1.00E-02	s^-1^	Fitted
ν_+_	Forward cooperativity factor for ligand binding a G bound receptor	1.00E+00		(Bridge et al., 2010)
ν_-_	Backward cooperativity factor for ligand binding	1.00E+00		“
ζ_+_	Forward cooperativity factor for ligand-bound R activation	1.00E+03		“
ζ_-_	Backward cooperativity factor for ligand bound R activation	1.00E+00		“
μ_+_	Forward cooperativity factor for G bound R activation	1.00E+00		“
μ_-_	Backward cooperativity factor for G bound R activation	1.00E+00		“
k_conact_	Constitutive activity of G_βɣ_	1.00E-10	s^-1^	Fitted
k_rev_	Reverse reaction of constitutive activity in G_βɣ_	1.00E-10	s^-1^	“
k_E+1_	STE5 binding rate to G_βɣ_^∗^	1.00E+3	M^-1^s^-1^	“
k_E+2_	STE5 binding rate to G_βɣ_	1.00E+9	M^-1^s^-1^	“
k_E-1_	STE5 dissociation rate to G_βɣ_^∗^	1.00E+0	s^-1^	“
k_E-2_	STE5 dissociation rate to G_βɣ_	1.00E+0	s^-1^	“
R_tot_	Total receptor concentration	4.15E-10	M	“
G_tot_	Total G protein concentration	4.15E-10	M	“
L_tot_	Total ligand concentration	1.00E-04	M	“

Since the model allows *G* to dissociate in the absence of Ligand *L* or *R^∗^* interaction, multiple levels of constitutive activity (elevated signaling in the absence of ligand) can be made possible. Along with the main pathway of *G* activation through *LR^∗^ G*_*ɑβγ*_, *R^∗^G*_*ɑβγ*_ may also activate the G protein cycle in the absence of ligand. *G*_*ɑβγ*_ can also dissociate and activate independently of *R* through rate constant k_GRA-_. To further reproduce constitutive features of the yeast pheromone cycle, inactive *G*_*βγ*_ may also be activated to be *G*_*βγ*_*^∗^* through k_conact_, which is a simplification of *G*_*βγ*_ interacting with downstream effectors. This detailed description of the constitutively active pathways allowed us to recapitulate the low constitutive signaling in the experimental results. To demonstrate how signaling decreases from an optimal point when increasing *G*_*βγ*_, it has been anticipated that at very high concentrations, *G*_*βγ*_ is unable to be trafficked to the plasma membrane due to the lack of *G*_*ɑ*_, but still affecting the pathway by binding to downstream effectors prematurely (Pryciak and Huntress, 1998, Song et al., 1996). This has been described simply in the model by showing how *G*_*βγ*_ and *G*_*βγ*_^∗^ compete for the same effector (Ste5). At low initial concentrations of *G*_*βγ*,_ little is available to bind to Ste5 so only the *G*_*βγ*_ that has been activated by interaction with the Ste2/Gpa1 complex (*G*_*βγ*_^∗^) will actively bind to Ste5 (due to its higher affinity). However, when the initial concentrations of *G*_*βγ*_ are increased free *G*_*βγ*_ is able to compete with the *G*_*βγ*_^∗^ and so sequesters the Ste5 species into an inactive complex so reducing the overall signaling maxima.

#### Ste2/Gpa1 Feedback Model

##### Rationale and Implementation

It has been well documented (Kofahl and Klipp, 2004, Shao et al., 2006) that the intracellular concentrations both Ste2 and Gpa1 increase upon pheromone stimulation. This arises due to positive feedback (at the transcriptional level – Ste12) thereby increasing the amount of available Ste2/Gpa1 present at the shmooing tip (Shao et al., 2006). Since expression of all components in our refactored yeast cells are driven through constitutive promoters, not pheromone-inducible ones, we wondered what impact positive feedback would have upon the response output. We therefore constructed a model derived from the Ste2/Gpa1 model described previously (see Methods S1A) but incorporating reaction rates to enable the concentration of either/both Ste2/Gpa1 to increase upon production of free G_βγ_ dimer (our model output). We hypothesized that increasing both Ste2 and Gpa1 concentrations (R and G respectively in the mode) through a positive feedback loop would enable enhanced maximal signaling (increased E_Max_) production while retaining a low basal activity through suppression by Gpa1 (Note Gpa1 acts to sequester free G_βγ_ so blocking signaling) (Whiteway et al., 1989).

##### Model Formulation

The formulation of the system of ODEs for the feedback model requires additional components to the previous system. Since the feedback loop is likely to involve multiple transcription/translation processes, our model required extension from the single ternary complex model to involve the whole refactored pheromone signaling pathway. In addition, long-term changes in the signaling components must also be considered. To retain a simplified model but that reflected dynamics at the Ste2/Gpa1 level but accounted for the MAPK cascade and gene expression we introduced two arbitrary molecular species (downstream of our readout free G_βγ_): X1 and X2. Both can either be in an inactive or active state (denoted X or X^∗^ respectively). Once X2 is in its active state it acts as a promoter, directly increasing concentrations of Ste2, Gpa1, or both. The basic scheme of the system of ODEs is denoted in Methods S1B.

The detailed system of ODEs can be found in Equation 5. In addition to the feedback loop, synthesis and degradation rates have also been considered in the parameter rates due to the longer timescale of the system. This relatively detailed system of ODEs consists of 8 receptor states, 5 non-receptor bound G protein states, and 4 downstream signaling states totaling to 17 ODEs. Model outputs would be concentrations [G_βγ_].

$$\frac{d\left[R\right]}{dt}=k_{L-}\left[LR\right]-k_{L+}\left[L\right]\left[R\right]+k_{act-}\left[R^{*}\right]-k_{act+}\left[R\right]+k_{G-}\left[RG\right]-k_{G+}\left[R\right]\left[G\right]+k_{Rsyn}-k_{Rint}\left[R\right]+k_{Gdeg}\left(\left[RG\right]\;+\;R^{*}G\right)+k_{Rfdbk}\left[X2^{*}\right]$$

$$\frac{d\left[LR\right]}{dt}=k_{L+}\left[L\right]\left[R\right]-k_{L-}\left[LR\right]+\zeta_{-}k_{act-}\left[LR^{*}\right]-\zeta_{+}k_{act+}\left[LR\right]+\nu_{-}k_{G-}\left[LRG\right]-\nu_{+}k_{G+}\left[LR\right]\left[G\right]-k_{Rint}\left[LR\right]+k_{Gdeg}\left[LRG\right]$$

$$\frac{d\left[R^{*}\right]}{dt}=k_{act+}\left[R\right]-k_{act-}\left[R^{*}\right]+\zeta_{-}k_{L-}\left[L\right]\left[R^{*}\right]-\zeta_{+}k_{L+}\left[L\right]\left[R^{*}\right]+\mu_{-}k_{G-}\left[R^{*}G\right]-\mu_{+}k_{G+}\left[R^{*}\right]+k_{GTP+}\left[R^{*}G\right]-k_{Rint}\left[R^{*}\right]$$

$$\frac{d\left[LR^{*}\right]}{dt}=\zeta_{+}k_{act+}\left[LR\right]-\zeta_{-}k_{act-}\left[LR^{*}\right]-\zeta_{-}k_{L-}\left[L\right]\left[R^{*}\right]-\zeta_{-}k_{L-}\left[LR^{*}\right]+\mu_{-}\nu_{-}k_{G-}\left[LR^{*}\right]-\mu_{+}\nu_{+}k_{G+}\left[LR^{*}\right]\left[G\right]+\nu_{-}k_{GTP+}\left[LR^{*}G\right]-k_{Rint}\left[LR^{*}\right]+k_{Gdeg}\left[LR^{*}G\right]$$

$$\frac{d\left[RG\right]}{dt}=k_{G+}\left[R\right]\left[G\right]-k_{G-}\left[RG\right]+\nu_{-}k_{L-}\left[LRG\right]-\nu_{+}k_{L+}\left[L\right]\left[RG\right]+\mu_{-}k_{act-}\left[R^{*}G\right]-\mu_{+}k_{act+}\left[RG\right]-k_{Rint}\left[RG\right]-k_{Gdeg}\left[RG\right]$$

$$\frac{d\left[LRG\right]}{dt}=\nu_{+}k_{G+}\left[LR\right]\left[G\right]-\nu_{-}k_{L-}\left[LRG\right]+\nu_{+}k_{L+}\left[L\right]\left[RG\right]-\nu_{-}k_{L-}\left[LRG\right]+\mu_{-}\zeta_{-}k_{act-}\left[LR^{*}G\right]-\mu_{+}\zeta_{+}k_{act+}\left[LRG\right]-k_{Rint}\left[LRG\right]-k_{Gdeg}\left[LRG\right]$$

$$\frac{d\left[R^{*}G\right]}{dt}=\mu_{+}k_{G+}\left[R^{*}\right]\left[G\right]-\mu_{-}k_{G-}\left[R^{*}G\right]+\mu_{+}k_{act+}\left[RG\right]-\mu_{-}k_{act-}\left[R^{*}G\right]-k_{GTP+}\left[R^{*}G\right]-\;k\_Rint\;\left[R^{*}G\right]-k\_Gdeg\;\left[R^{*}G\right]$$

$$\frac{d\left[LR^{*}G\right]}{dt}=\mu_{+}\nu_{+}k_{G+}\left[LR^{*}\right]\left[G\right]-\mu_{-}\nu_{-}k_{G-}\left[LR^{*}\right]+\zeta_{+}\nu_{+}k_{L+}\left[L\right]\left[R^{*}G\right]-\zeta_{-}\nu_{-}k_{L-}\left[LR^{*}G\right]+\mu_{+}\zeta_{+}k_{act+}\left[LRG\right]-\mu_{-}\zeta_{-}k_{act-}\left[LR^{*}G\right]-\nu_{-}k_{GTP+}\left[LR^{*}G\right]-k_{Rint}\left[LR^{*}G\right]-k_{Gdeg}\left[LR^{*}G\right]$$

$$\frac{d\left[G\right]}{dt}=k_{G-}\left[RG\right]-k_{G+}\left[R\right]\left[G\right]+\nu_{-}k_{L-}\left[LRG\right]\;-\nu_{+}k_{G+}\left[LR\right]\left[G\right]+k_{GRA+}\left[\alpha_{GDP}\right]\left[\beta \gamma \right]+k_{GRA+}\left[\alpha_{GDP}\right]\left[\beta \gamma^{*}\right]-k_{GRA-}\left[G\right]+\mu_{-}k_{G-}\left[R^{*}G\right]-\mu_{+}k_{G+}\left[R^{*}\right]\left[G\right]+\mu_{-}\nu_{-}k_{G-}\left[LR^{*}\right]\;-\mu_{+}\nu_{+}k_{G+}\left[LR^{*}\right]\left[G\right]+k_{Gsyn}-k_{Gdeg}\left[G\right]+\;k_{Rint}\left(\left[RG\right]+\left[R^{*}G\right]+\left[LRG\right]+\left[LR^{*}G\right]\right)+\;k_{Gfdbk}\left[X2^{*}\right]$$

$$\frac{d\left[\alpha_{GDP}\right]}{dt}=k_{hyd+}\left[\alpha_{GTP}\right]-k_{hyd-}\left[\alpha_{GDP}\right]+k_{GRA-}\left[G\right]-k_{GRA+}\left[\alpha_{GDP}\right]\left[\beta \gamma \right]+k_{\alpha GDPfdbk}\left[X2^{*}\right]-k_{Gdeg}\left[\alpha_{GDP}\right]$$

$$\frac{d\left[\alpha_{GTP}\right]}{dt}=k_{hyd-}\left[\alpha_{GDP}\right]\;-k_{hyd+}\left[\alpha_{GTP}\right]+k_{GTP+}\left[R^{*}G\right]+\nu_{-}k_{GTP+}\left[LR^{*}G\right]+k_{\alpha GTPfdbk}\left[X2^{*}\right]-k_{Gdeg}\left[\alpha_{GTP}\right]$$

$$\frac{d\left[\beta \gamma \right]}{dt}=k_{GRA-}\left[G\right]+k_{GTP+}\left[R^{*}G\right]+\nu_{-}k_{GTP+}\left[LR^{*}G\right]-k_{GRA+}\left[\alpha_{GDP}\right]\left[\beta \gamma \right]+k_{\beta \gamma fdbk}\left[X2^{*}\right]-k_{Gdeg}\left[\beta \gamma \right]$$

$$\frac{d\left[X1\right]}{dt}=k_{x1-}\left[X1^{*}\right]-k_{X1+}\left[\beta \gamma \right]\left[X1\right]$$

$$\frac{d\left[X2\right]}{dt}=k_{x2-}\left[X2^{*}\right]-k_{X2+}\left[X1^{*}\right]\left[X2\right]$$

$$\frac{d\left[X1^{*}\right]}{dt}=k_{X1+}\left[\beta \gamma \right]\left[X1\right]-k_{x1-}\left[X1^{*}\right]$$

(Equation 5) $$\frac{d\left[X2^{*}\right]}{dt}=k_{X2+}\left[\beta \gamma \right]\left[X2\right]-\;k_{x2-}\left[X2^{*}\right]$$

#### Simulation Results

Here we present the numerical results to illustrate the Ste2 and Gpa1 feedback model. Final parameter values can be found below. ODE results shown are endpoint concentration of [G_βγ_] after 1000 time points, with a 1e^8^ time point equilibration (Bridge et al., 2018, Croft et al., 2013, Smith et al., 2009). The simulated results seem to show similar trends against the experimental data, with an increase in maximum while retaining a low basal activity to the system (Figures S4F and S4G).

#### Parameter Values Used to Demonstrate the Yeast Feedback

**Table:** 

Parameter	Meaning	Values	Units
k_L+_	Ligand binding rate	9.40E+04	M^-1^s^-1^
k_L-_	Ligand unbinding rate	3.10E-01	s^-1^
k_act+_	Receptor activation rate to R^∗^	1.00E+00	s^-1^
k_act-_	Receptor deactivation rate from R^∗^	1.00E+03	s^-1^
k_G+_	G protein binding rate	1.00E+08	M^-1^s^-1^
k_G-_	G protein unbinding rate	1.00E-01	s^-1^
k_GRA+_	G protein re-association rate	7.00E+08	M^-1^s^-1^
k_GRA-_	G protein dissociation rate	1.30E-03	s^-1^
k_hyd+_	Hydrolysis rate of G_αGTP_	1.00E-01	s^-1^
k_hyd-_	Exchange rate of GTP to GDP at G_α_	1.00E-04	s^-1^
k_GTP+_	R^∗^G dissociation rate	1.00E+00	s^-1^
k_RGS_	RGS activity rate to hydrolyze G_ɑGTP_	4.00E+05	s^-1^
k_X1+_	X1 activation rate to X1^∗^	2.00E-03	s^-1^
k_X1-_	X1^∗^ inactivation rate to X1	2.00E-04	s^-1^
k_X2+_	X2 activation rate to X2^∗^	2.00E-03	s^-1^
k_X2-_	X2^∗^ inactivation rate to X2	2.00E-04	s^-1^
k_Rsyn_	Receptor synthesis rate	1.20E-14	s^-1^
k_Rint_	Receptor internalization rate	2.90E-04	s^-1^
k_RGSsyn_	RGS synthesis rate	3.30E-11	s^-1^
k_RGSdeg_	RGS degradation rate	3.00E-04	s^-1^
k_Gsyn_	G protein synthesis rate	9.55E-14	s^-1^
k_Gdeg_	G protein degradation rate	2.30E-04	s^-1^
ν_+_	Forward cooperativity factor for ligand binding a G bound receptor	1.00E+00	
ν_-_	Backward cooperativity factor for ligand binding	1.00E+00	
ζ_+_	Forward cooperativity factor for ligand-bound R activation	1.00E+03	
ζ_-_	Backward cooperativity factor for ligand bound R activation	1.00E+00	
μ_+_	Forward cooperativity factor for G-bound R activation	1.00E+00	
μ_-_	Backward cooperativity factor for G bound R activation	1.00E+00	
R_tot_	Total receptor concentration	4.15E-10	M
L_tot_	Total ligand concentration	1.00E-04	M
X1_init_	Total X1 concentration	1.00E-07	M
X2_init_	Total X2 concentration	1.00E-07	M

#### Digital Sensor Model

##### Rationale and Implementation

An application demonstrating the flexibility in the operational range of the yeast GPCR-based sensors was to use mixed populations of communicating strains to narrow the range, creating concentration-response curves close to a digital, ‘all or none’ response. The aim of this digital sensor model was to describe, in detail, the interactions between yeast cells in which their properties are modified so the product of one cell (ɑ-factor; Amplifier cell) becomes the agonist of another (Reporter cell). The Reporter cell expresses, under the constitutive *RPL18B* promoter, Bar1, a protease which degrades ɑ-factor (Sprague and Herskowitz, 1981). It has been suggested that the presence of Bar1 reduces ‘non-productive mating’ events in *S. cerevisiae* by rapidly degrading low levels of ɑ-factor (Segota and Franck, 2017). In essence, Bar1 works as a barrier between the ɑ-factor producing cell and the reporter cell, degrading the low levels of constitutively released ɑ-factor from the Amplifier cell. As concentrations of stimulating agonist (melatonin) increase, the proteolytic activity of Bar1 is saturated with increased ɑ-factor, allowing the activation of the reporter strain.

##### Model Formulation

Our previous modeling efforts have concentrated upon the dynamics of the R/G_ɑβγ_ complex. However, to enable qualitative modeling of the digital sensor (effectively two entire pheromone signaling cascades) we decided to use a heavily reduced model for the R/G_ɑβγ_ complex. We felt this important since our focus is on the interactions/behaviors of the two cells rather than solely the G protein cycle within a cell. Thus, we have chosen a reduced model which accurately describes overall *in vivo* responses for time- and dose-dependent effects in the system.

To deviate from processes within the cell and focus on the interactions between cells, a reduced yeast pheromone pathway based on a model developed by Smith et al. (2009) was used. The structure of the reduced model consisting of 9 ODEs for each cell, modified to fit the experimental system through the addition of Bar1 interaction with ɑ-factor, along with a modification to set a maximum amount of possible product each cell can produce.

The overall scheme used for the model is shown in Methods S1C. In this system, receptor states are simplified to two (*R* and *R^∗^*) and G protein states are simplified to three species (*G*_*off*_, *G*_*on*_, and *G*_*on*_*Effector*). The activated G protein will interact with an Effector protein (Ste5) which consequently activates a series of delay species to simulate the MAPK-like signaling cascade. *prez*_*3*_ is a term responsible for creating the final product of each cell by being directly converted to its corresponding product. It will act as a ‘cap’ by limiting maximum production within the concentration of *prez*_*3*_. Constitutive activity of each of the cell is achieved by directly adding *GFP* (the product generated from the reporter cell) to the system.

The detailed system of ODEs can be found in Equation 6. Synthesis and degradation of the system are omitted for simplicity, apart from *R^∗^* internalization. Our system includes the full pheromone response of two yeast cells, interactions between the two cells, and the constitutive protease activity of Bar1 consisting of 23 ODEs. Model outputs are either the product of the ɑ-factor producing cell (*ɑfactor*) or the product of the reporter cell (*GFP*), depending on the setup of the assay.

$$\frac{d\left[R_{1}\right]}{dt}=\left[R_{1}^{*}\right]k_{71}-\left[L\right]\left[R_{1}\right]k_{11}-\left[R_{1}\right]k_{con1}$$

$$\frac{d\left[R_{1}^{*}\right]}{dt}=\left[L\right]\left[R_{1}\right]k_{11}-\left[R_{1}^{*}\right]k_{71}-\left[R_{1}^{*}\right]k_{41}+\left[R_{1}\right]k_{con1}$$

$$\frac{d\left[G_{off1}\right]}{dt}=\left[G_{on1}\right]k_{31}-\left[R_{1}^{*}\right]\left[G_{off1}\right]k_{21}$$

$$\frac{d\left[G_{on1}\right]}{dt}=\left[R_{1}^{*}\right]\left[G_{off1}\right]k_{21}+\left[G_{on1}Effector_{1}\right]k_{61}-\left[G_{on1}\right]k_{31}-\left[G_{on1}\right]\left[Effector_{1}\right]k_{51}$$

$$\frac{d\left[Effector_{1}\right]}{dt}=\left[G_{on1}Effector_{1}\right]k_{61}-\left[G_{on1}\right]\left[Effector_{1}\right]k_{51}$$

$$\frac{d\left[G_{on1}Effector_{1}\right]}{dt}=\left[G_{on1}\right]\left[Effector_{1}\right]k_{51}-\left[G_{on1}Effector_{1}\right]k_{61}$$

$$\frac{d\left[z_{11}\right]}{dt}=\left[G_{on1}Effector_{1}\right]\alpha_{1}-\left[z_{11}\right]\beta_{1}$$

$$\frac{d\left[z_{21}\right]}{dt}=\left[z_{11}\right]\left[\alpha_{11}\right]-\left[z_{21}\right]\beta_{11}$$

$$\frac{d\left[pre\alpha factor\right]}{dt}=\left[\alpha factor\right]\beta_{21}-\left[z_{21}\right]\left[pre\alpha factor\right]\alpha_{21}$$

$$\frac{d\left[\alpha factor\right]}{dt}=\left[z_{21}\right]\left[pre\alpha factor\right]\alpha_{21}-\left[\alpha factor\right]\beta_{21}-\left[\alpha factor\right]\left[R_{2}\right]k_{12}+\left[R_{2}^{*}\right]k_{72}-\left[Bar1\right]\left[\alpha factor\right]k_{8}$$

$$\frac{d\left[R_{2}\right]}{dt}=\left[R_{2}^{*}\right]k_{72}-\left[\alpha factor\right]\left[R_{2}\right]k_{12}-\left[R_{2}\right]k_{con2}$$

$$\frac{d\left[R_{2}^{*}\right]}{dt}=\left[\alpha factor\right]\left[R_{2}\right]k_{12}-\left[R_{2}^{*}\right]k_{72}-\left[R_{2}^{*}\right]k_{42}+\left[R_{2}\right]k_{con2}$$

$$\frac{d\left[G_{off2}\right]}{dt}=\left[G_{on2}\right]k_{32}-\left[R_{2}^{*}\right]\left[G_{off2}\right]k_{22}$$

$$\frac{d\left[G_{on2}\right]}{dt}=\left[R_{2}^{*}\right]\left[G_{off2}\right]k_{22}-\left[G_{on2}Effector_{2}\right]k_{62}-\left[G_{on2}\right]k_{32}-\left[G_{on2}\right]\left[Effector_{2}\right]k_{52}$$

$$\frac{d\left[Effector_{2}\right]}{dt}=\left[G_{on2}Effector_{1}\right]k_{62}-\left[G_{on2}\right]\left[Effector_{2}\right]k_{52}$$

$$\frac{d\left[G_{on2}Effector_{2}\right]}{dt}=\left[G_{on2}\right]\left[Effector_{2}\right]k_{52}-\left[G_{on2}Effector_{2}\right]$$

$$\frac{d\left[z_{12}\right]}{dt}=\left[G_{on2}Effector_{2}\right]\alpha_{2}-\left[z_{12}\right]\beta_{2}$$

$$\frac{d\left[z_{22}\right]}{dt}=\left[z_{12}\right]\left[\alpha_{12}\right]-\left[z_{22}\right]\beta_{12}$$

$$\frac{d\left[preGFP\right]}{dt}=\left[GFP\right]\beta_{22}-\left[z_{22}\right]\left[preGFP\right]\alpha_{22}$$

$$\frac{d\left[GFP\right]}{dt}=\left[z_{22}\right]\left[preGFP\right]\alpha_{22}-\left[GFP\right]\beta_{22}$$

$$\frac{d\left[L\right]}{dt}=\left[R_{1}^{*}\right]k_{71}-\left[L\right]\left[R_{1}\right]k_{11}$$

$$\frac{d\left[Bar1\right]}{dt}=\left[InactiveBar1\right]k_{9}+\left[InactiveBar1\right]k_{10}-\left[Bar1\right]\left[\alpha factor\right]k_{8}$$

(Equation 6) $$\frac{d\left[InactiveBar1\right]}{dt}=\left[Bar1\right]\left[\alpha factor\right]k_{8}-\left[InactiveBar1\right]k_{9}-\left[InactiveBar1\right]k_{10}$$

#### Parameter Estimation

260-minute endpoint readings of the refactored melatonin-sensitive yeast cell (MTNR1A sensor), Digital Feedback including the reporter cell without constitutive Bar1 activity, and Digital Feedback with Bar1 activity data were fitted simultaneously. Since most of the yeast pheromone system except the core MAPK cascade were refactored within the system, parameter values related to the receptor and its subsequent G protein activation rates were fitted using COPASI 4.16 (Hoops et al., 2006). Moreover, parameters and species introduced in the system for the first time were fitted against experimental data mentioned above. The set of finalized parameter values were obtained through parameter estimation algorithms provided in COPASI. To simulate and accurately fit incubation times in COPASI, constitutive fluorescence has been set as the initial concentrations of the corresponding products (3.1 nM for MTNR1A sensor, 10.4 nM for digital feedback without Bar1, and 4.4 nM for digital feedback with Bar1). Two optimization algorithms were tested: the Hooke and Jeeves algorithm and the evolutionary programming algorithm. Both methods reliably converged to the same minimum within the parameter space, which were thus considered as a global minimum.

#### Simulation Results

Here we present the parameter values and fitted graphs of the model, which can be found below. Ligand and Receptor specific parameters were refitted to the experimental time course data, as well as Bar1 interaction and initial concentration. Initial concentrations of Receptor, G_off_, and Effector were set consistently at 16 μM for both cells.

#### Parameter Values Used for the Double Cell System

**Table:** 

Parameter	Meaning	Values	Units	Source
k_11_	Ligand binding rate	2.75E8	M^-1^s^-1^	Fitted
k_12_		1.40E5	M^-1^s^-1^	“
k_21_	Receptor activation rate to R^∗^	2726.24	s^-1^	(Smith et al., 2009)
k_22_		1.21E4	s^-1^	“
k_31_	G protein unbinding rate	4.00E-3	s^-1^	“
k_32_		4.00E-3	s^-1^	“
k_41_	R^∗^ internalization rate	1.06E-7	s^-1^	Fitted
k_42_		3.08E-8	s^-1^	“
k_51_	Effector binding rate	1.04E6	M^-1^s^-1^	(Smith et al., 2009)
k_52_		1.04E6	M^-1^s^-1^	“
k_61_	Effector unbinding rate	0.0942	s^-1^	“
k_62_		0.0942	s^-1^	“
k_71_	Ligand unbinding rate	86.98	s^-1^	Fitted
k_72_		2.14E-7	s^-1^	“
α_1_	Transcriptional delay	17.75		(Smith et al., 2009)
α_2_		17.75		“
β_1_		1859.57		“
β_2_		1859.57		“
α_11_		42.41		“
α_12_		42.41		“
β_11_		0.93		“
β_12_		0.93		“
α_21_	prez_3_ activation rate	2.80E6	s^-1^	Fitted
α_22_		6.63E9	s^-1^	“
β_21_	prez_3_ inactivation rate	6.32E-7	s^-1^	“
β_22_		4.90E-8	s^-1^	“
k_8_	ɑ-factor inactivation rate	4.19E8	M^-1^s^-1^	“
k_9_	Bar1 recycle rate	4.35E-6	s^-1^	“
k_10_	Bar1/ɑ-factor degradation rate	3.38E4	s^-1^	“
preɑ-factor_tot_	Total preɑ-factor concentration	4.66E-8	M	“
preGFP_tot_	Total preGFP concentration	1.52E-8	M	“
Bar1_tot_	Total Bar1 concentration	1.68E-10	M	“

Final figures and fitting results created through the model are shown in Methods S1D. Fitting results demonstrate a lower E_Max_, higher Hill slope and increased potency upon introduction of Bar1, which was also observed in the ‘wet’ experimental data (Methods S1D–S1H). To provide an estimate of quality of the fitting of the model to the biological data we used residual analysis (Methods S1E), by which each residual is taken from the deviation of an observed value against the predicted value (Motulsky and Christopoulos, 2003). Residual values are defined in Equation 7.

(Equation 7) $$e_{i}\;=\;y_{i}\;-\;f_{i}$$

Where a dataset has *n* values marked *y*_*1*_*,…,y*_*n*_ (collectively defined as y_i_), the predicted values marked *f*_*1*_*,…f*_*n*_ (collectively *f*_*i*_). All residual plots suggest an unbiased distribution with an equal deviation from 0. The residual plot digital feedback without Bar1 suggest a good fitting result with an unbiased distribution and a consistent variance throughout. Although heteroscedasticity may be observed in MTNR1A sensor and the digital feedback with Bar1, differences in variation within each ligand concentration suggests the natural tendency for variation to occur under the system with higher ligand concentration. Nevertheless, the residual plots suggest an unbiased distribution of experimental results. R^2^ values also suggest and give some information about the goodness of fit, defined in Equation 8.

(Equation 8) $$R^{2}=1-\frac{\sum_{i}e_{i}^{2}}{\sum_{i}{\left(y_{i}-\bar{y}\right)}^{2}}$$

With $\bar{y}$ as the mean of the dataset. Along with a R^2^ value of 97.93%, 99.90%, and 99.68% for the MTNR1A sensor, the digital feedback without Bar1, and digital feedback with Bar1 respectively, the non-linear regression suggests a good fit with the experimental data.

Using the model, we simulate the effects of increasing concentrations of Bar1 (Figure S7C). At low concentrations of Bar1 constitutive signaling is decreased while obtaining similar or slightly lower concentrations close to the E_Max_. These biological responses suggest that the reporter cell has a ‘capped’ response where from a defined concentration, the reporter cell will not exceed its maximum capacity to produce *GFP*. This interpretation enables the reporter cell to experience *ɑfactor* saturation and even with a relatively small concentration of *Bar1* it will not be able to degrade *ɑfactor* sufficiently at higher [L] (Figure S7D). With the introduction of Bar1, basal activity can be reduced while retaining the maximum possible response of the system.

#### Generation of Ultrasensitivity

Ultrasensitivity, or the ability to create a digitalized response from a graduated input within a steady state response, is a well-studied field in systems biology (Zhang et al., 2013) and is said to be possible through various motifs such as multistep, feed-forward cooperativity, zero-order, and inhibitor ultrasensitivity. Within this ODE model, it has been designed to have two different mechanisms of ultrasensivity: through the amplification of the reporter cell and inhibition through Bar1. The MAPK cascade is a well known pathway where ultrasensitivity can arise from multistep-phosphorylation, positive feedback, and the cooperativity obtained through scaffolding protein, which is referred to as Ste5 in yeast (Ferrell, 1996, Shao et al., 2006, Zhang et al., 2013). Bar1 interaction is also considered as a source of ultrasensitivity, where a competitive inhibitor will essentially compete against the Ste2 receptor.

### Quantification and Statistical Analysis

Statistical tests of all experiments was performed using GraphPad Prism version 7 or MathWorks MATLAB version R2017a and are detailed within the legend of each figure. For flow cytometry experiments, fluorescence data was collected from 10,000 cells for each datapoint and analyzed using FlowJo software, representing the average fluorescence of the population as the geometric mean. In all figures, the data points represent the mean ± SD. Curves fitted to all dose-response data was fitted in Prism 7 using the Nonlinear Regression: Variable slope (four parameter) curve fitting. One-way analysis of variance (ANOVA) was used to determine signifiance between the mean growth rates of the wild-type, model, and sensor yeast strains. Residual analysis was used to provide an estimate of quality of the fitting of the Cell-Cell Digital model to the biological data, by which each residual is taken from the deviation of an observed value against the predicted value (Motulsky and Christopoulos, 2003).

### Data and Software Availability

All part plasmids described in this paper are available at Addgene (Addgene ID: 123024-123065). Corrected nanopore sequencing reads for the yWS677 model strain are available at Sequence Read Archive (SRA) (SRA accession: PRJNA516326). MATLAB code for the mathematical models in this paper are available at BioModels Database (BioModels ID: Cubic Ternary Complex Model, MODEL1901300001; Cell-To-Cell Digital Sensor Model, MODEL1901300002).

## Supporting Citations

The following references appear in the Supplemental Information: Baranski et al. (1999); Brown et al., 2000, Brown et al., 2003, Brown et al., 2011; Campbell et al. (1999); Chambers et al. (2000); Erickson et al. (1998); Erlenbach et al., 2001a, Erlenbach et al., 2001b; Evans et al. (2009); Fowler et al. (1999); Gaibelet et al. (1999); Huang et al. (2015); Kajkowski et al. (1997); King et al. (1990); Klein et al. (1998); Larsen et al. (2012); Leplatois et al. (2001); Liu et al. (2016); Miret et al. (2002); Olesnicky et al. (1999); Overton et al. (2006); Price et al. (1995); Sander et al. (1994); Scott et al. (2019); and Weston et al., 2014, Weston et al., 2015.

## Supplemental Information

Table S1. Functional GPCRs in *S. cerevisiae*, Related to Figure 6

Table S2. Oligonucleotides Used in This Study, Related to STAR Methods

Table S3. Part Sequences, Related to STAR Methods Part sequences in YTK format (Lee et al., 2015). Grey highlight – BsaI recognition site. Pink highlight – BsmBI recognition site. Bold – BsaI/BsmBI generated overhang. Blue highlight – GFP dropout. Green highlight – start codon. Red highlight – stop codon. Underlined – open reading frame. Gpa1-Gɑ chimeras were generated by assembling annealed phosphorylated oligonucleotides into the Gpa1 C-terminal Truncation Vector (pWS936) using BsmBI Golden Gate assembly. See Table S2 for a list of oligonucleotides used to generate the Gɑ library.

Table S4. Plasmids Used in This Study, Related to STAR Methods Plasmids listed here are organized by figure number and then by their name within the figure. Each row within the description corresponds to a particular cassette or multigene cassette, indicating the selection marker. All plasmids are integration cassettes integrating at either the *URA3* (*URA3* marker), *LEU2* (*LEU2* marker) or *HO* (*HIS3* marker) locus.

Methods S1. Model Schematics, Related to STAR Methods (A) The detailed reaction scheme of the cubic ternary complex model modified for the refactored *S. cerevisiae* pathway. Reactions can be divided into modules of which involves Receptor activation (Top) and G protein cycle (Bottom). Black letters each represent a species within a system, and all arrows are marked in red letters with rate constants k_●_ and corresponding cooperativity factors μ, ν, and ζ, which produce a unique rate constant for each reaction. (B) The detailed reaction scheme of the single ternary complex model modified for the refactored *S. cerevisiae* pathway. Reactions can be divided into modules of which involves Receptor activation (Top), G protein cycle (Middle), and the downstream cascade (Bottom). Black letters each represent a species within a system, and all arrows are marked in red letters with rate constants k_●_ and corresponding cooperativity factors μ, ν, and ζ, which produce a unique rate constant for each reaction. (C) Schematic diagram of the double cell system. (Left) Signaling system within a cell. Each cell contains the corresponding set of kinetic rates and species. (Right) Simplified version of the full system, including the interaction of Bar1 and α-factor and its degradation. (D) Overall fitting results against time course data as concentration-response curves taken through endpoint readings. Solid shapes represent experimental results reading fluorescence 260 minutes after the addition of ligand. Solid lines represent ODE results derived from time courses of 260 minutes. Blue represents the system with the α-factor producing cell only and red represents the system with two cells with Bar1. (E) Residual plots of the experimental concentration-response curves against the computational fitting results. (Left) Residual plots of the MTNR1A sensor. (Middle) Residual plots of the experimental results of digital feedback without Bar1. (Right) Residual plots of the experimental results of digital feedback with Bar1. (F) Abbreviations used in the model schematics.
