# Supplementary material for: Engineering a Model Cell for Rational Tuning of GPCR Signaling
Source: Cell. 2019 Apr 18;177(3):782–796.e27. doi: 10.1016/j.cell.2019.02.023 (PMC6476273; doi:10.1016/j.cell.2019.02.023)
Supplement: Table S1. Functional GPCRs in S. cerevisiae, Related to Figure 6 [file mmc1.pdf]

Cell, Volume *177*

Supplemental Information

Engineering a Model Cell

for Rational Tuning of GPCR Signaling

William M. Shaw, Hitoshi Yamauchi, Jack Mead, Glen-Oliver F. Gowers, David J. Bell, David Öling, Niklas Larsson, Mark Wigglesworth, Graham Ladds, and Tom Ellis

Sub-class	GPCR name	Species	Ligand	Reference
5-Hydroxytryptamine receptors	5-HT4 receptor	Human	Serotonin	(Ehrenworth et al., 2017)
Acetylcholine receptors (muscarinic)	M1 receptor	Human	Acetylcholine	(Erlenbach et al., 2001a)
	M3 receptor	Rat	Acetylcholine	(Erlenbach et al., 2001a)
	M5 receptor	Human	Acetylcholine	(Erlenbach et al., 2001a)
Adenosine receptors	A1 receptor	Human	Adenosine	(Campbell et al., 1999)
	A2A receptor	Human	Adenosine	(Campbell et al., 1999)
	A2B receptor	Human	Adenosine	(Brown et al., 2000)
Adrenoceptors	β2-adrenoceptor	Human	Epinephrine	(King et al., 1990)
Adrenomedullin	AM1 receptor	Human	Adrenomedullin	(Miret et al., 2002)
	AM2 receptor	Human	Adrenomedullin	(Miret et al., 2002)
Cannabinoid receptors	CB1 receptor	Human	Cannabinoids	(Brown et al., 2011)
	CB2 receptor	Human	Cannabinoids	(Brown et al., 2011)
CGRP	CGRP receptor	Human	CGRP	(Miret et al., 2002)
Chemokine receptors	CXCR4	Human	SDF1	(Evans et al., 2009)
Class A Orphans	GPR119	Human	Oleoylethanolamide	(Overton et al., 2006)
	GPR35	Human	Cannabinoids	(Brown et al., 2011)
	GPR55	Human	Cannabinoids	(Brown et al., 2011)
Complement peptide receptors	C5a1 receptor	Human		(Baranski et al., 1999)
Dopamine receptor	D2S	Human	Dopamine	(Sander et al., 1994)
Formylpeptide receptors	FPR1	Human	Formylpeptide	(Klein et al., 1998)
Free fatty acid receptors	FFA2 Receptor	Human	Short-chain fatty acids	(Brown et al., 2003)
	FFA3 Receptor	Human	Short-chain fatty acids	(Brown et al., 2003)
	GPR40	Human	Medium-chain fatty acids	(Mukherjee et al., 2015)
Glucagon receptor family	GHRH receptor	Human	GHRH	(Kajkowski et al., 1997)
	GCGR receptor	Human	Glucagon	(Weston et al., 2015)
	GLP-1 receptor	Human	GLP-1	(Weston et al., 2014)
Hydroxycarboxylic acid receptors	HCA2 receptor	Human	Niacin	(Liu et al., 2016)
	HCA3 receptor	Human	Niacin	(Liu et al., 2016)
Lysophosphatidic acid (LPA) receptors	LPA receptor 1	Human	Lysophosphatidic acid	(Erickson et al., 1998)
Melatonin receptors	MT1 receptor	Human	Melatonin	(Kokkola et al., 1998)
	MT2 receptor	Human	Melatonin	(Brown et al., 2000)
Neurotensin	NTS1 receptor	Human	Neurotensin	(Leplatois et al., 2001)
Olfactory receptor	OR1G1	Human	Medium chain fatty acids	(Mukherjee et al., 2015)
Opioid receptor	MOR receptor	Human	Opioids	(Gaibelet et al., 1999)
Orphan receptors	GPR68	Human	Benzodiazepine	(Huang et al., 2015)
P2Y receptors	P2Y1 receptor	Human	Nucleotides	(Brown et al., 2000)
	P2Y1 receptor	Human	Nucleotides	(Brown et al., 2000)
	P2Y14 receptor	Human	UDP-glucose	(Chambers et al., 2000)
Peptide pheromone receptors	RCb2	*C. cinerus*	Peptide pheromone	(Olesnicky et al., 1999)
	RCb3	*C. cinerus*	Peptide pheromone	(Olesnicky et al., 1999)
	Bbr1, 2	*S. commune*	Peptide pheromone	(Fowler et al., 1999)
	FLP2	*C. elegans*	Peptide pheromone	(Larsen et al., 2013)
	Ce.Ste2	*C. glabrata*	Peptide pheromone	(Ostrov et al., 2017)
	Ca.Ste2	*C. albicans*	Peptide pheromone	(Ostrov et al., 2017)
	Le.Ste2	*L. elongisporus*	Peptide pheromone	(Ostrov et al., 2017)
	Pb.Ste2	*P. brasiliensis*	Peptide pheromone	(Ostrov et al., 2017)
	Bc.Ste2	*B. cinerea*	Peptide pheromone	(Ostrov et al., 2017)
	Fg.Ste2	*F. graminearum*	Peptide pheromone	(Ostrov et al., 2017)
	Mo.Ste2	*M. oryzae*	Peptide pheromone	(Ostrov et al., 2017)
	Zb.Ste2	*Z. bailii*	Peptide pheromone	(Ostrov et al., 2017)
	Zr.Ste2	*Z. rouxii*	Peptide pheromone	(Ostrov et al., 2017)
Rhodopsin	RHO receptor	Human	Light	(Scott et al., 2018)
Somatostatin receptors	SST2 receptor	Human	Somatostatin	(Brown et al., 2000)
	SST2 receptor	Rat	Somatostatin	(Price et al., 1995)
	SST5 receptor	Human	Somatostatin	(Brown et al., 2000)
Vasopressin	V2 receptor	Human	Vasopressin	(Erlenbach et al., 2001b)
