# Supplementary material for: Engineering a Model Cell for Rational Tuning of GPCR Signaling
Source: Cell. 2019 Apr 18;177(3):782–796.e27. doi: 10.1016/j.cell.2019.02.023 (PMC6476273; doi:10.1016/j.cell.2019.02.023)
Supplement: Table S3. Part Sequences, Related to STAR Methods — Part sequences in YTK format (Lee et al., 2015). Grey highlight – BsaI recognition site. Pink highlight – BsmBI recognition site. Bold – BsaI/BsmBI generated overhang. Blue highlight – GFP dropout. Green highlight – start codon. Red highlight – stop codon. Underlined – open reading frame. Gpa1-Gɑ chimeras were generated by assembling annealed phosphorylated oligonucleotides into the Gpa1 C-terminal Truncation Vector (pWS936) using BsmBI Golden Gate assembly. See Table S2 for a list of oligonucleotides used to generate the Gɑ library. [file mmc3.pdf]

**pWS060 - pFUS1 [2]**	GGTCTCA**AACG**CCATATTTACCATGTGGACCCTTTCAAAACAGAGTTGTATCTCTGCAGGATGCCCTTTTTGACGTATTGAATGGCATAATT<br>GCACTGTCACTTTTCGCGCTGTCTCATTTTGGTGCGATGATGAAACAAACATGAAACGTCTGTAATTTGAAACAAATAACGTAATTCTCGGG<br>ATTGGTTTTATTTAAATGACAATGTAAGAGTGGCTTTGTAAGGTATGTGTTGCTCTTAAAATATTTGGATACGACATCCTTTATCTTTTTTCCT<br>TTAAGAGCAGGATATAAGCCATCAAGTTTCTGAAAATCAAAGATC**TATG**TGAGACC
**pWS053 – pFIG1 [2]**	GGTCTCA**AACG**GAACTGGTTGATATTATTACTGGTGCTTCCTCTTTGGGATGATAAAAAAATCACCCTGCATTGCCTCTTTATTTGACGTTG<br>TTTTTGTAGAACATGAAACGAATTTTGACTTGATGACACGAAGTATATATCCAAAGAATACCTTAAATAGAAAAGGAAAGATAATAAATACTA<br>AACACTACTATATATTCAGGTAAAATACAAAAATTATAACATTTTTAAAACTTTTTTTTTTTGAAAGTCCTTCTCGCTTTAGGATTTTTTCCCAT<br>TAAGATTATGATGGTTTCATGTATGTGTCAGTTAAAAAAAATATGGCTAAGTAGCAATGAAACGAACCAAGAAAATGAAAACAATATATAGTG<br>CTGTTGAAATAACAAAGACATTGGTATATATTTGTAAAATGTCTGTTAAATGTTTTTTATCTCAGGTTCTTGCTTGTCTTTGGTAGAAGAAATT<br>ATAGTAAACAAACAAACAAACAAACAAAAAAAAAAAAAAAAAAAAAGATC**TATG**TGAGACC
**pWS2247 - pTetA [2]**	GGTCTCA**AACG**GGTAGTCCATCGTTGTAGGATACTCCCTATCAGTGATAGAGAAATCTATGCGGCATCCCTATCAGTGATAGAGATCGGTA<br>ACGGAGTCCCTATCAGTGATAGAGAAGTGGTGTTCAGTTCCCTATCAGTGATAGAGAAGTTTGACACTATCCCTATCAGTGATAGAGAGAA<br>CAGCAATGACTCCCTATCAGTGATAGAGATCAAATGGCGTATCCCTATCAGTGATAGAGATAATTAACTTGTAATATTCTAATCAATTGACAA<br>TATTATTTAAGGACCTATTGTTTTTTCCAATAGGTGGTTAGCAATCGTCTTACTTTCTAACTTTTCTTACCTTTTACATTTCAGCAATATATATA<br>TATATATTTCAAGGATATACCATTCTAAGATC**TATG**TGAGACC
**pWS1078 - pZ3 [2]**	GGTCTCA**AACG**CCCCATTATCTTAGCCTAAAAAAACCTTCTCTTTGGAACTTTCAGTAATACGCTTAACTGCTCATTGCTATATTGAAGTACG<br>GCCGCGTGGGCGTGCGTGGGCGGGCGTGGGCGTGCGTGGGCGGGCGTGGGCGTGCGTGGGCGTCTAGACCGTGCGTCCTCGTCATC<br>ACCGGTCGCGTTCCTGAAACGCAGATGTGCCTCGCGCCGCACTGCTCCGAACAATAAAGATTCTACAATACTAGCTTTTATGGTTATGAAG<br>AGGAAAAAATTGGCAGTAACCTGGCCCCACAAACCTTCAAATTAACGAATCAAATTAACAACCATAGGATGATAATGCGATTAGTTTTTTAGC<br>CTTATTTCTGGGGTAATTAATCAGCGAAGCGATGATTTTTGATCTATTAACAGATATATAAATGGAAAAGCTGCATAACCACTTTAACTAATA<br>CTTTCAACATTTTCAGTTTGTATTACTTCTTATTCAAATGTCATAAAAGTATCAACAAAAAATTGTTAATATACCTCTATACTTTAACGTCAAGG<br>AGAAAAAACTATAAGATC**TATG**TGAGACC
**pWS480 – LexO (8x) [2a]**	GGTCTCA**AACG**GGTAGTCCATCGTTGTAGGATACTGTATATACATACAGTAGTCACGTCGTTTACTGTATATACTCACAGTACGCTATTGCA<br>ACTACTGTATATACACCCAGTAGAGTAGGTGACTACTGTATGAGCATACAGTAGTGACAACCACTTACTGTATATAAATACAGTAGTGGTCA<br>TCGATACTGTATATAAAACCAGTAGGCAATCGTTTGTACTGTATGTACATACAGTATACCTCGCAACTACTGTATATAAACACAGTAATTAAC<br>TTGTAATATTCTAATCAA**TTGA**TGAGACC
**pWS481 – LexO (6x) [2a]**	GGTCTCA**AACG**GGTAGTCCATCGTTGTAGGATACTGTATATACACCCAGTAGAGTAGGTGACTACTGTATGAGCATACAGTAGTGACAACC<br>ACTTACTGTATATAAATACAGTAGTGGTCATCGATACTGTATATAAAACCAGTAGGCAATCGTTTGTACTGTATGTACATACAGTATACCTCG<br>CAACTACTGTATATAAACACAGTAATTAACTTGTAATATTCTAATCAA**TTGA**TGAGACC
**pWS482 – LexO (4x) [2a]**	GGTCTCA**AACG**GGTAGTCCATCGTTGTAGGATACTGTATATAAATACAGTAGTGGTCATCGATACTGTATATAAAACCAGTAGGCAATCGTT<br>TGTACTGTATGTACATACAGTATACCTCGCAACTACTGTATATAAACACAGTAATTAACTTGTAATATTCTAATCAA**TTGA**TGAGACC
**pWS483 – LexO (3x) [2a]**	GGTCTCA**AACG**GGTAGTCCATCGTTGTAGGATACTGTATATAAAACCAGTAGGCAATCGTTTGTACTGTATGTACATACAGTATACCTCGCA<br>ACTACTGTATATAAACACAGTAATTAACTTGTAATATTCTAATCAA**TTGA**TGAGACC
**pWS484 – LexO (2x) [2a]**	GGTCTCA**AACG**GGTAGTCCATCGTTGTAGGATACTGTATGTACATACAGTATACCTCGCAACTACTGTATATAAACACAGTAATTAACTTGT<br>AATATTCTAATCAA**TTGA**TGAGACC
**pWS485 – LexO (1x) [2a]**	GGTCTCA**AACG**GGTAGTCCATCGTTGTAGGATACTGTATATAAACACAGTAATTAACTTGTAATATTCTAATCAA**TTGA**TGAGACC
**pWS494 – pRNR1m [2b]**	GGTCTCA**TTGA**TATATAAAGGAGCTAATATTTCATTGTTGGAAAATTACTCTACCATAATTGAAGCATATCTCATCCTTTTCATCCTTTTCAAC<br>GCAAGAGAGACACCAACGAACAACACTTTATTTGTTGATATATTAACATCAGATC**TATG**TGAGACC
**pWS491 – pTEF2m [2b]**	GGTCTCA**TTGA**TATATAAATTCTCTTGCATTTTCTATTTTTCTCTCTATCTATTCTACTTGTTTATTCCCTTCAAGGTTTTTTTTTAAGGAGTAC<br>TTGTTTTTAGAATATACGGTCAACGAACTATAATTAACTAAACAGATC**TATG**TGAGACC
**pWS487 – pTDH3m [2b]**	GGTCTCA**TTGA**TATATAAAGACGGTAGGTATTGATTGTAATTCTGTAAATCTATTTCTTAAACTTCTTAAATTCTACTTTTATAGTTAGTCTTTT<br>TTTTAGTTTTAAAACACCAAGAACTTAGTTTCGAATAAACACACATAAACAAACAAAAGATC**TATG**TGAGACC
**pWS493 – pALD6m [2b]**	GGTCTCA**TTGA**TATATAAATGTAATAAGAAGTTTGGTAATATTCAATTCGAAGTGTTCAGTCTTTTACTTCTCTTGTTTTATAGAAGAAAAAAC<br>ATCAAGAAACATCTTTAACATACACAAACACATACTATCAGAATACAAGATC**TATG**TGAGACC
**pRC246 – pLEU2m [2b]**	GGTCTCA**TTGA**CAATATTATTTAAGGACCTATTGTTTTTTCCAATAGGTGGTTAGCAATCGTCTTACTTTCTAACTTTTCTTACCTTTTACATTT<br>CAGCAATATATATATATATATATTTCAAGGATATACCATTCTAAGATC**TATG**TGAGACC
**pWS486 – pGAL1m [2b]**	GGTCTCA**TTGA**TATATAAATGGAAAAGCTGCATAACCACTTTAACTAATACTTTCAACATTTTCAGTTTGTATTACTTCTTATTCAAATGTCAT<br>AAAAGTATCAACAAAAAATTGTTAATATACCTCTATACTTTAACGTCAAGGAGAAAAAACTATAAGATC**TATG**TGAGACC
**pWS498 – pPHO5m [2b]**	GGTCTCA**TTGA**TATATAAGCGCTGATGTTTTGCTAAGTCGAGGTTAGTATGGCTTCATCTCTCATGAGAATAAGAACAACAACAAATAGAGC<br>AAGCAAATTCGAGATTACCAAGATC**TATG**TGAGACC
**pWS496 – pCYC1m [2b]**	GGTCTCA**TTGA**TATATAAAACTCTTGTTTTCTTCTTTTCTCTAAATATTCTTTCCTTATACATTAGGACCTTTGCAGCATAAATTACTATACTTC<br>TATAGACACACAAACACAAATACACACACTAAATTAATAAGATC**TATG**TGAGACC
**pWS497 – pCUP1m [2b]**	GGTCTCA**TTGA**TATAAAAGAGAAGCAAATAACTCCTTGTCTTGTATCAATTGCATTATAATATCTTCTTGTTAGTGCAATATCATATAGAAGTC<br>ATCGAAATAGATATTAAGAAAAACAAACTGTACAATCAATCAATCAATCATCACATAAAAGATC**TATG**TGAGACC

**pWS495 – pRNR2m [2b]**	GGTCTCA**TTGA**TATATATAGCGGTAGTGTTTGCGCGTTACCATCATCTTCTGGATCTATCTATTGTTCTTTTCCTCATCACTTTCCCCTTTTTC<br>GCTCTTCTTCTTGTCTTTTATTTCTTTCTTTTTTTTAATTGTTCCCTCGATTGGCTATCTACCAAAGAATCCAAACTTAATACACGTATTTATTT<br>GTCCAATTACCAGATC**TATG**TGAGACC
**pWS067 – Ste2 [3]**	GGTCTCA**TATG**TCTGATGCGGCTCCTTCATTGAGCAATCTATTTTATGATCCAACGTATAATCCTGGTCAAAGCACCATTAACTACACTTCC<br>ATATATGGGAATGGATCTACCATCACTTTCGATGAGTTGCAAGGTTTAGTTAACAGTACTGTTACTCAGGCCATTATGTTTGGTGTCAGATG<br>TGGTGCAGCTGCTTTGACTTTGATTGTCATGTGGATGACATCGAGAAGCAGAAAAACGCCGATTTTCATTATCAACCAAGTTTCATTGTTTT<br>TAATCATTTTGCATTCTGCACTCTATTTTAAATATTTACTGTCTAATTACTCTTCAGTGACTTACGCTCTCACCGGATTTCCTCAGTTCATCAG<br>TAGAGGTGACGTTCATGTTTATGGTGCTACAAATATAATTCAAGTCCTTCTTGTGGCTTCTATTGAGACTTCACTGGTGTTTCAGATAAAAGT<br>TATTTTCACAGGCGACAACTTCAAAAGGATAGGTTTGATGCTGACGTCGATATCTTTCACTTTAGGGATTGCTACAGTTACCATGTATTTTGT<br>AAGCGCTGTTAAAGGTATGATTGTGACTTATAATGATGTTAGTGCCACCCAAGATAAATACTTCAATGCATCCACAATTTTACTTGCATCCTC<br>AATAAACTTTATGTCATTTGTCCTGGTAGTTAAATTGATTTTAGCTATTAGATCAAGAAGATTCCTTGGTCTTAAGCAGTTCGATAGTTTCCAT<br>ATTTTACTCATAATGTCATGTCAATCTTTGTTGGTTCCATCGATAATATTCATCCTCGCATACAGTTTGAAACCAAACCAGGGAACAGATGTC<br>TTGACTACTGTTGCAACATTACTTGCTGTATTGTCTTTACCATTATCATCAATGTGGGCCACGGCTGCTAATAATGCATCCAAAACAAACACA<br>ATTACTTCAGACTTTACAACATCCACAGATAGGTTTTATCCAGGCACGCTGTCTAGCTTTCAAACTGATAGTATCAACAACGATGCTAAAAG<br>CAGTCTCAGAAGTAGATTATATGACCTATATCCTAGAAGGAAGGAAACAACATCGGATAAACATTCGGAAAGAACTTTTGTTTCTGAGACTG<br>CAGATGATATAGAGAAAAATCAGTTTTATCAGTTGCCCACACCTACGAGTTCAAAAAATACTAGGATAGGACCGTTTGCTGATGCAAGTTAC<br>AAAGAGGGAGAAGTTGAACCCGTCGACATGTACACTCCCGATACGGCAGCTGATGAGGAAGCCAGAAAGTTCTGGACTGAAGATAATAAT<br>AATTTAGG**ATCC**TGAGACC
**pWS626 – Mam2 [3]**	GGTCTCA**TATG**AGGCAACCTTGGTGGAAAGACTTTACTATTCCCGATGCAAGCGCAATTATTCACCAAAATATTACCATTGTGTCTATTGTC<br>GGAGAGATTGAAGTGCCTGTTTCAACAATTGATGCATATGAAAGGGATAGGTTATTAACTGGAATGACTTTATCTGCCCAATTAGCTTTAGG<br>AGTGTTAACCATTTTAATGGTTTGTCTGTTATCATCAAGCGAAAAACGTAAACACCCTGTTTTTGTTTTTAATTCGGCAAGTATTGTTGCAAT<br>GTGTTTACGCGCCATTTTAAATATCGTGACCATCTGCTCGAATTCGTACAGTATCCTCGTTAATTACGGGTTTATCTTAAACATGGTTCATAT<br>GTATGTGCATGTGTTTAATATTTTAATTTTATTATTAGCACCGGTGATCATTTTTACTGCTGAGATGTCGATGATGATTCAAGTTAGGATCATT<br>TGTGCACATGATAGGAAAACACAACGTATCATGACTGTTATTAGTGCCTGCTTAACTGTTTTAGTTCTCGCATTTTGGATTACTAACATGTGT<br>CAACAGATTCAGTATCTCTTATGGTTAACTCCTTTATCGTCGAAAACCATTGTTGGATACTCTTGGCCCTACTTTATTGCTAAAATCTTATTTG<br>CTTTTTCGATTATTTTTCACAGTGGTGTTTTTTCATACAAACTCTTTAGGGCCATCTTAATCCGCAAAAAAATTGGGCAATTTCCTTTTGGTCC<br>GATGCAGTGTATTTTAGTTATTTCGTGCCAATGTTTAATTGTTCCTGCTACCTTTACTATCATCGATAGTTTTATCCATACGTATGATGGCTTT<br>TCGTCTATGACTCAATGTCTCCTGATCATTTCTTTACCATTATCGAGTTTATGGGCGTCTAGTACAGCTCTCAAATTACAATCGATGAAAACT<br>TCATCTGCGCAAGGAGAAACCACCGAGGTTTCGATTAGGGTTGATAGGACGTTTGATATCAAACATACTCCCAGTGACGATTATTCGATTT<br>CTGATGAATCTGAAACTAAAAAGTGGACGGG**ATCC**TGAGACC
**pWS625 – A2BR [3]**	GGTCTCA**TATG**TTGTTGGAAACACAAGATGCGTTGTATGTTGCGTTGGAATTGGTAATTGCTGCGCTATCGGTTGCGGGAAATGTTTTGGT<br>TTGTGCTGCGGTTGGAACGGCGAATACCTTGCAAACGCCTACTAATTATTTTTTGGTTTCATTGGCAGCGGCTGATGTTGCTGTTGGACTAT<br>TTGCTATTCCTTTCGCTATTACTATTTCTTTGGGATTTTGTACCGATTTTTATGGATGTCTATTTCTAGCTTGTTTTGTTTTGGTTCTAACGCAA<br>TCTTCAATTTTTTCTCTATTGGCTGTTGCCGTAGATAGGTATTTGGCTATTTGCGTACCGCTAAGGTACAAGTCCCTTGTAACGGGAACTCG<br>TGCCAGGGGAGTAATAGCAGTACTATGGGTACTAGCTTTCGGAATTGGACTTACCCCTTTTTTGGGATGGAATTCCAAGGATTCCGCTACT<br>AATAATTGTACAGAGCCTTGGGACGGAACTACGAACGAGTCTTGTTGTCTAGTTAAATGCCTATTCGAAAACGTTGTACCTATGTCTTATAT<br>GGTGTACTTTAACTTTTTCGGATGCGTGTTGCCTCCTTTGCTAATCATGTTGGTTATTTATATAAAAATTTTTTTGGTTGCTTGTAGGCAACTA<br>CAACGTACCGAATTGATGGATCATTCGAGGACTACTCTACAAAGGGAAATTCACGCCGCTAAATCCTTGGCTATGATAGTTGGAATATTCG<br>CTTTGTGTTGGCTCCCTGTTCACGCAGTGAATTGCGTAACCCTATTTCAACCTGCACAAGGCAAGAACAAACCTAAATGGGCCATGAACAT<br>GGCTATACTATTGTCCCACGCTAACTCCGTGGTAAACCCTATAGTATACGCATATAGGAATCGTGATTTTCGTTATACCTTCCATAAGATAAT<br>TTCAAGGTACCTACTATGTCAGGCCGACGTAAAATCCGGAAACGGCCAAGCAGGAGTGCAACCTGCACTAGGCGTTGGACTCGG**ATCC**TG<br>AGACC
**pWS1806 – MTNR1A [3]**	GGTCTCA**TATG**CAAGGTAATGGTTCTGCTTTGCCAAATGCTTCTCAACCAGTTTTGAGAGGTGATGGTGCTAGACCTTCTTGGTTGGCTTCT<br>GCTTTAGCTTGTGTTTTGATTTTCACCATCGTTGTCGATATCTTGGGTAACTTGTTGGTTATCTTGTCCGTCTACCGTAACAAGAAATTGAGA<br>AACGCTGGTAACATCTTCGTTGTTTCTTTGGCTGTTGCTGATTTGGTTGTTGCTATCTATCCATATCCACTGGTCTTGATGTCCATTTTTAAC<br>AACGGTTGGAACTTGGGTTACTTGCATTGTCAAGTTTCTGGTTTCTTGATGGGTTTGTCCGTTATTGGTTCCATTTTCAACATTACCGGTATC<br>GCCATTAACAGGTACTGTTACATTTGCCACTCACTGAAGTACGACAAGTTGTACTCTTCTAAGAACTCCTTGTGCTACGTTTTGTTGATCTG<br>GTTGTTAACTTTGGCTGCTGTTTTGCCTAATTTGAGAGCTGGTACATTGCAATACGATCCAAGAATCTACTCTTGTACCTTCGCTCAATCTGT<br>TTCTTCTGCTTACACTATTGCCGTTGTCGTTTTCCATTTTTTGGTCCCAATGATTATCGTCATCTTCTGCTACTTGAGAATCTGGATTTTGGTC<br>TTGCAAGTCAGACAAAGAGTTAAGCCAGATAGAAAGCCAAAATTGAAGCCACAAGACTTCAGAAACTTCGTTACCATGTTTGTGGTTTTCGT<br>TTTGTTCGCTATTTGTTGGGCTCCATTGAACTTTATTGGTTTGGCAGTTGCTTCTGATCCAGCTTCTATGGTTCCAAGAATTCCAGAATGGTT<br>GTTCGTTGCTTCTTACTACATGGCTTACTTCAACTCTTGTTTGAACGCCATTATCTACGGCTTGTTGAATCAGAACTTTAGGAAAGAGTACAG<br>GCGTATCATCGTTTCTTTGTGTACTGCTAGAGTTTTCTTCGTCGATTCCTCTAATGATGTTGCCGATAGAGTTAAGTGGAAACCATCTCCATT<br>GATGACCAACAACAATGTTGTCAAGGTTGACTCCGTTGG**ATCC**TGAGACC
**pWS2119 – PbSte2 [3]**	GGTCTCA**TATG**GCACCCTCATTCGACCCCTTCAACCAAAGCGTGGTGTTCCACAAGGCCGACGGAACTCCATTCAACGTGTCAATCCATG<br>AACTAGACGACTTCGTGCAGTACAACACCAAAGTCTGCATCAACTACTCTTCCCAGCTCGGAGCATCTGTCATTGCAGGACTCATGCTTGC<br>CATGCTGACACACTCAGAAAAGCGTCGTCTGCCAGTTTTCTTCCTAAACACATTCGCACTGGCCATGAACTTTGCCCGCCTGCTCTGCATG<br>ACCATCTACTTCACCACGGGCTTCAACAAGTCCTATGCCTACTTTGGTCAGGATTACTCCCAGGTGCCTGGGAGCGCCTACGCAGCCTCT<br>GTCTTGGGCGTTGTGTTCACCACTCTCCTGGTAATCAGCATGGAAATGTCCCTCCTGATCCAAACAAGGGTTGTCTGCACGACCCTTCCGG<br>ATATCCAACGTTATCTACTCATGGCAGTTTCCTCCGCGATTTCCCTGATGGCCATCGGGTTCCGCCTTGGCTTAATGGTTGAGAACTGCAT<br>TGCCATTGTGCAGGCGTCGAATTTCGCCCCTTTTATCTGGCTTCAAAGCGCCTCGAACATCACCATTACGATCAGCACATGTTTCTTCAGT<br>GCCGTCTTTGTTACGAAATTGGCATATGCACTCGTCACTCGTATACGACTAGGCTTGACGAGGTTTGGTGCTATGCAGGTTATGTTCATCAT<br>GTCCTGCCAGACTATGGTGATTCCAGCCATCTTCTCAATTCTCCAATACCCACTCCCCAAGTACGAAATGAACTCCAACCTCTTTACGCTGG<br>TGGCCATTTTCCTCCCTCTTTCCTCGCTATGGGCTTCAGTTGCTACGAGATCCAGTTTCGAGACATCTTCTTCCGGCCGCCATCAGTATCTT<br>TGGCCAAGCGAACAGAGCAATAACGTCACCAATTCGGAAATTAAGTATCAGGTCAGCTTCTCTCAGAACCACACTACGTTGCGGTCTGGAG<br>GGTCTGTGGCCACGACACTCTCCCCGGACCGGCTCGACCCGGTTTATTGTGAAGTTGAAGCTGGCACAAAGGCCGG**ATCC**TGAGACC
**pWS1037 - Gpa1 [3-4a]**	GGTCTCA**TATG**GGGTGTACAGTGAGTACGCAAACAATAGGTGACGAAAGTGATCCTTTTCTACAGAACAAAAGAGCCAATGATGTCATCGA<br>GCAATCGTTGCAGCTGGAGAAACAACGTGACAAGAATGAAATAAAACTGTTACTATTAGGTGCCGGTGAGTCAGGTAAATCAACGGTTTTA<br>AAACAATTAAAATTATTACATCAAGGCGGTTTCTCCCATCAAGAAAGGTTACAGTATGCTCAAGTGATATGGGCAGATGCCATACAATCAAT<br>GAAAATTTTGATTATTCAGGCCAGAAAACTAGGTATTCAACTTGACTGTGATGATCCGATCAACAATAAAGATTTGTTTGCATGCAAGAGAAT<br>ACTGCTAAAGGCTAAAGCTTTAGATTATATCAACGCCAGTGTTGCCGGTGGTTCTGATTTTCTAAATGATTATGTACTGAAGTACTCAGAAA<br>GGTATGAAACTAGGAGGCGTGTTCAGAGTACCGGACGAGCAAAAGCTGCTTTCGATGAGGACGGAAATATTTCTAATGTCAAAAGTGACA<br>CTGACAGAGATGCTGAAACGGTGACGCAAAATGAGGATGCTGATAGAAACAACAGTAGTAGAATTAACCTACAGGATATTTGCAAGGACTT<br>GAACCAAGAAGGCGATGACCAGATGTTTGTTAGAAAAACATCAAGGGAAATTCAAGGACAAAATAGACGAAATCTTATTCACGAGGACATT<br>GCTAAGGCAATAAAGCAACTTTGGAATAACGACAAAGGTATAAAGCAGTGTTTTGCACGTTCTAATGAGTTTCAATTGGAGGGCTCAGCTG<br>CATACTACTTTGATAACATTGAGAAATTTGCTAGTCCGAATTATGTCTGTACGGATGAGGACATTTTGAAGGGCCGTATAAAGACTACAGGC<br>ATTACAGAAACCGAATTTAACATCGGCTCGTCCAAATTCAAGGTTCTCGACGCTGGTGGGCAGCGTTCTGAACGTAAGAAGTGGATTCATT<br>GTTTCGAAGGAATTACAGCAGTTTTATTTGTTTTAGCAATGAGTGAATACGACCAGATGTTGTTTGAGGATGAAAGAGTGAACAGAATGCAT<br>GAATCAATAATGCTATTTGACACGTTATTGAACTCTAAGTGGTTCAAAGATACACCGTTTATTTTGTTTTTAAATAAAATTGATTTGTTCGAGG<br>AAAAGGTAAAAAGCATGCCCATAAGAAAGTACTTTCCTGATTACCAGGGACGTGTCGGCGATGCAGAAGCGGGTCTAAAATATTTTGAGAA<br>GATATTTTTGAGCTTGAATAAGACAAACAAACCAATCTACGTGAAACGAACCTGCGCTACCGATACCCAAACTATGAAGTTCGTATTGAGTG<br>CAGTCACCGATCTAATCATCCAGCAAAACCTGAAAAAAATTGGTATTATTTAACTCGAG**TGGC**TGAGACC
**pWS936 – Gpa1 C-**	GGTCTCA**TATG**GGGTGTACAGTGAGTACGCAAACAATAGGTGACGAAAGTGATCCTTTTCTACAGAACAAAAGAGCCAATGATGTCATCGA<br>GCAATCGTTGCAGCTGGAGAAACAACGTGACAAGAATGAAATAAAACTGTTACTATTAGGTGCCGGTGAGTCAGGTAAATCAACGGTTTTA

**terminal Truncation Vector [3-4a]**	AAACAATTAAAATTATTACATCAAGGCGGTTTCTCCCATCAAGAAAGGTTACAGTATGCTCAAGTGATATGGGCAGATGCCATACAATCAAT<br>GAAAATTTTGATTATTCAGGCCAGAAAACTAGGTATTCAACTTGACTGTGATGATCCGATCAACAATAAAGATTTGTTTGCATGCAAGAGAAT<br>ACTGCTAAAGGCTAAAGCTTTAGATTATATCAACGCCAGTGTTGCCGGTGGTTCTGATTTTCTAAATGATTATGTACTGAAGTACTCAGAAA<br>GGTATGAAACTAGGAGGCGTGTTCAGAGTACCGGACGAGCAAAAGCTGCTTTCGATGAGGACGGAAATATTTCTAATGTCAAAAGTGACA<br>CTGACAGAGATGCTGAAACGGTGACGCAAAATGAGGATGCTGATAGAAACAACAGTAGTAGAATTAACCTACAGGATATTTGCAAGGACTT<br>GAACCAAGAAGGCGATGACCAGATGTTTGTTAGAAAAACATCAAGGGAAATTCAAGGACAAAATAGACGAAATCTTATTCACGAGGACATT<br>GCTAAGGCAATAAAGCAACTTTGGAATAACGACAAAGGTATAAAGCAGTGTTTTGCACGTTCTAATGAGTTTCAATTGGAGGGCTCAGCTG<br>CATACTACTTTGATAACATTGAGAAATTTGCTAGTCCGAATTATGTCTGTACGGATGAGGACATTTTGAAGGGCCGTATAAAGACTACAGGC<br>ATTACAGAAACCGAATTTAACATCGGCTCGTCCAAATTCAAGGTTCTCGACGCTGGTGGGCAGCGTTCTGAACGTAAGAAGTGGATTCATT<br>GTTTCGAAGGAATTACAGCAGTTTTATTTGTTTTAGCAATGAGTGAATACGACCAGATGTTGTTTGAGGATGAAAGAGTGAACAGAATGCAT<br>GAATCAATAATGCTATTTGACACGTTATTGAACTCTAAGTGGTTCAAAGATACACCGTTTATTTTGTTTTTAAATAAAATTGATTTGTTCGAGG<br>AAAAGGTAAAAAGCATGCCCATAAGAAAGTACTTTCCTGATTACCAGGGACGTGTCGGCGATGCAGAAGCGGGTCTAAAATATTTTGAGAA<br>GATATTTTTGAGCTTGAATAAGACAAACAAACCAATCTACGTGAAACGAACCTGCGCTACCGATACCCAAACTATGAAGTTCGTATTGAGTG<br>CAGTCACCGATCTAATCATCCAGCAAAACCT**GAAA**CGAGACGGAAAGTGAAACGTGATTTCATGCGTCATTTTGAACATTTTGTAAATCTTA<br>TTTAATAATGTGTGCGGCAATTCACATTTAATTTATGAATGTTTTCTTAACATCGCGGCAACTCAAGAAACGGCAGGTTCGGATCTTAGCTAC<br>TAGAGAAAGAGGAGAAATACTAGATGCGTAAAGGCGAAGAGCTGTTCACTGGTGTCGTCCCTATTCTGGTGGAACTGGATGGTGATGTCA<br>ACGGTCATAAGTTTTCCGTGCGTGGCGAGGGTGAAGGTGACGCAACTAATGGTAAACTGACGCTGAAGTTCATCTGTACTACTGGTAAACT<br>GCCGGTTCCTTGGCCGACTCTGGTAACGACGCTGACTTATGGTGTTCAGTGCTTTGCTCGTTATCCGGACCATATGAAGCAGCATGACTTC<br>TTCAAGTCCGCCATGCCGGAAGGCTATGTGCAGGAACGCACGATTTCCTTTAAGGATGACGGCACGTACAAAACGCGTGCGGAAGTGAAA<br>TTTGAAGGCGATACCCTGGTAAACCGCATTGAGCTGAAAGGCATTGACTTTAAAGAGGACGGCAATATCCTGGGCCATAAGCTGGAATACA<br>ATTTTAACAGCCACAATGTTTACATCACCGCCGATAAACAAAAAAATGGCATTAAAGCGAATTTTAAAATTCGCCACAACGTGGAGGATGGC<br>AGCGTGCAGCTGGCTGATCACTACCAGCAAAACACTCCAATCGGTGATGGTCCTGTTCTGCTGCCAGACAATCACTATCTGAGCACGCAA<br>AGCGTTCTGTCTAAAGATCCGAACGAGAAACGCGATCATATGGTTCTGCTGGAGTTCGTAACCGCAGCGGGCATCACGCATGGTATGGAT<br>GAACTGTACAAATGACCAGGCATCAAATAAAACGAAAGGCTCAGTCGAAAGACTGGGCCTTTCGTTTTATCTGTTGTTTGTCGGTGAACGC<br>TCTCTACTAGAGTCACACTGGCTCACCTTCGGGTGGGCCTTTCTGCGTTTATACGTCTCT**TGGC**TGAGACC
**pWS939 – Ste12 [3]**	GGTCTCA**TATG**AAAGTCCAAATAACCAATAGTAGAACAGAGGAAATCTTAAAAGTTCAAGCTAATAATGAAAACGATGAAGTCAGTAAAGCT<br>ACTCCGGGCGAAGTTGAAGAATCGCTAAGGTTAATCGGCGATCTAAAATTCTTTTTAGCCACAGCGCCGGTAAATTGGCAAGAAAACCAAA<br>TTATAAGGCGATACTATCTGAATAGTGGACAAGGCTTTGTCTCTTGTGTATTTTGGAACAATCTATACTATATTACAGGTACTGATATTGTCA<br>AATGTTGTCTTTACAGAATGCAAAAGTTTGGGAGAGAAGTAGTTCAAAAGAAAAAATTTGAAGAGGGTATTTTTTCAGATTTAAGAAATCTCA<br>AATGTGGTATAGATGCAACTTTAGAACAACCAAAGTCCGAATTTTTGTCGTTTCTATTCAGAAATATGTGTCTGAAAACCCAAAAAAAGCAGA<br>AAGTATTTTTTTGGTTCAGTGTAGCACATGATAAGTTGTTTGCGGATGCGTTGGAAAGAGATTTAAAAAGAGAAAGTTTGAATCAGCCTTCA<br>ACGACTAAGCCCGTTAATGAGCCCGCCTTATCTTTTTCATATGATTCCTCATCTGATAAGCCTCTCTACGATCAGTTACTTCAACATTTAGAT<br>TCTAGAAGGCCATCTAGTACAACAAAATCAGATAATTCGCCTCCAAAATTAGAAAGCGAGAATTTTAAGGATAATGAGTTGGTAACAGTAAC<br>TAATCAGCCGCTTTTAGGCGTTGGCCTCATGGATGACGATGCGCCAGAATCCCCCTCTCAAATTAATGATTTTATTCCTCAGAAATTGATTA<br>TAGAACCCAATACTCTCGAATTGAATGGTCTAACAGAAGAAACGCCTCATGACTTACCCAAGAATACCGCTAAGGGCAGAGATGAAGAAGA<br>TTTTCCTCTCGACTATTTTCCTGTATCTGTTGAATACCCTACGGAGGAAAATGCGTTTGATCCGTTCCCTCCACAGGCTTTTACGCCAGCTG<br>CCCCTTCCATGCCTATTTCCTATGATAACGTGAATGAAAGGGATTCTATGCCCGTTAATTCTCTTCTTAATAGATACCCCTATCAGTTATCAG<br>TGGCACCCACTTTCCCAGTGCCACCATCATCATCGAGGCAACATTTTATGACAAATCGGGATTTTTATTCATCTAACAATAACAAGGAAAAA<br>TTGGTATCTCCTAGCGACCCTACCAGCTACATGAAGTATGACGAACCAGTTATGGATTTTGATGAATCTCGGCCAAATGAAAACTGTACAAA<br>TGCAAAATCTCACAACTCTGGCCAGCAAACTAAACAACACCAATTATATTCTAACAACTTCCAGCAATCTTACCCAAACGGAATGGTTCCAG<br>GATACTACCCAAAAATGCCGTATAATCCCATGGGGGGGGATCCTCTACTCGATCAAGCCTTTTATGGCGCGGACGATTTTTTCTTTCCACC<br>AGAAGGATGTGATAACAATATGCTGTATCCACAAACTGCAACTTCATGGAATGTTTTGCCCCCTCAAGCTATGCAACCAGCTCCAACCTATG<br>TTGGGAGGCCATACACACCGAATTATAGATCGACACCAGGTTCCGCGATGTTCCCATACATGCAAAGTTCAAATTCCATGCAGTGGAACAC<br>TGCTGTTTCACCTTATAGTTCGAGAGCACCATCTACAACTGCTAAAAACTATCCTCCTAGCACATTTTATTCTCAAAATATAAATCAATACCC<br>ACGGCGAAGAACTGTGGGAATGAAGTCATCACAAGGAAATGTTCCAACAGGTAATAAACAATCTGTGGGCAAGTCTGCAAAAATTTCAAAG<br>CCTCTACATATTAAGACAAGTGCTTATCAGAAGCAATACAAAATCAACTTGGAAACGAAAGCCAGGCCAAGTGCTGGTGACGAAGATTCTG<br>CTCATCCTGATAAGAACAAAGAAATTTCGATGCCTACTCCGGATTCCAATACTTTGGTGGTCCAGTCAGAAGAAGGTGGAGCTCATTCACTT<br>GAGGTAGATACCAATCGAAGGTCCGATAAAAACCTTCCAGATGCAACCGG**ATCC**TGAGACC
**pWS926 – LexA-NLS [3a]**	GGTCTCA**TATG**AAGGCTTTAACTGCAAGACAACAGGAAGTTTTTGATTTGATAAGAGATCATATATCTCAAACTGGAATGCCACCAACAAGA<br>GCTGAAATAGCTCAAAGATTAGGATTTAGATCTCCAAACGCTGCAGAAGAACATTTGAAAGCTTTGGCAAGGAAAGGTGTCATCGAAATTG<br>TCTCTGGTGCTTCAAGAGGTATTAGACTATTACAAGAAGAGGAAGAAGGATTGCCATTGGTTGGTAGAGTTGCTGCTGGTGAACCATTATT<br>AGCACAGCAACATATTGAGGGTCATTATCAAGTAGATCCATCTTTATTCAAACCAAATGCTGATTTTTTGTTGAGAGTAAGTGGTATGTCTAT<br>GAAAGATATTGGTATCATGGATGGTGACTTATTGGCTGTTCATAAAACTCAAGATGTTAGAAACGGTCAAGTTGTCGTTGCCAGAATTGATG<br>ATGAAGTCACTGTTAAAAGATTGAAGAAACAAGGTAATAAAGTGGAATTGTTACCAGAAAATTCTGAATTTAAGCCAATTGTTGTTGATTTAA<br>GGCAACAATCTTTTACTATTGAAGGTTTAGCTGTCGGTGTTATCAGAAATGGTGATTGGTTAGGTTCAGGTTCTCCTCCAAAAAAAAAGAGA<br>AAAGTTGGATCTGG**TTCT**TGAGACC
**pWS931 – TetR-NLS [3a]**	GGTCTCA**TATG**TCTAGATTAGATAAAAGTAAAGTGATTAACAGCGCATTAGAGCTGCTTAATGAGGTCGGAATCGAAGGTTTAACAACCCGT<br>AAACTCGCCCAGAAGCTAGGTGTAGAGCAGCCTACATTGTATTGGCATGTAAAAAATAAGCGGGCTTTGCTCGACGCCTTAGCCATTGAGA<br>TGTTAGATAGGCACCATACTCACTTTTGCCCTTTAGAAGGGGAAAGCTGGCAAGATTTTTTACGTAATAACGCTAAAAGTTTTAGATGTGCT<br>TTACTAAGTCATCGCGATGGAGCAAAAGTACATTTAGGTACACGGCCTACAGAAAAACAGTATGAAACTCTCGAAAATCAATTAGCCTTTTT<br>ATGCCAACAAGGTTTTTCACTAGAGAATGCATTATATGCACTCAGCGCTGTGGGGCATTTTACTTTAGGTTGCGTATTGGAAGATCAAGAGC<br>ATCAAGTCGCTAAAGAAGAAAGGGAAACACCTACTACTGATAGTATGCCGCCATTATTACGACAAGCTATCGAATTATTTGATCACCAAGGT<br>GCAGAGCCAGCCTTCTTATTCGGCCTTGAATTGATCATATGCGGATTAGAAAAACAACTTAAATGTGAAAGTGGATCAGGTTCTCCTCCAAA<br>AAAAAAGAGAAAAGTTGGATCTGG**TTCT**TGAGACC
**pWS930 – Z3E [3a]**	GGTCTCA**TATG**AGGCCCTACGCTTGCCCTGTAGAAAGTTGTGATAGGAGATTCAGCAGATCTGACGAGCTGACTAGGCATATTCGTATTCA<br>CACCGGCCAAAAGCCTTTCCAATGTAGAATCTGTATGAGAAACTTCAGCAGAAGTGACCATTTGACTACGCACATTAGAACCCACACCGGC<br>GAGAAGCCGTTCGCGTGTGACATTTGTGGAAGAAAGTTCGCCAGATCTGATGAAAGAAAGAGACATACAAAGATCCATACAGGAGGTGGG<br>GGCACTCCGGCAGCAGCCTCAACCCTAGAAGATCCAAGCGCTGGAGACATGAGGGCAGCCAACTTGTGGCCAAGCCCATTAATGATCAA<br>AAGATCTAAGAAGAATTCTCTTGCACTGTCTTTGACCGCTGATCAAATGGTTTCCGCACTACTTGATGCTGAACCACCTATATTGTACAGTG<br>AATACGACCCAACTCGTCCTTTTTCCGAGGCTTCCATGATGGGTCTTTTGACAAATCTAGCAGACAGGGAATTAGTTCATATGATTAATTGG<br>GCCAAAAGAGTTCCCGGCTTTGTTGACTTGACATTACATGACCAGGTACATCTTCTTGAATGCGCGTGGTTAGAAATATTGATGATCGGATT<br>AGTGTGGAGAAGTATGGAGCACCCTGTAAAATTATTATTTGCACCTAATTTATTATTGGACAGGAACCAAGGTAAATGCGTAGAAGGTATGG<br>TTGAGATTTTTGATATGCTTCTTGCCACTAGCTCCAGGTTCAGAATGATGAATTTGCAAGGAGAAGAATTCGTTTGCCTGAAATCAATTATTT<br>TGTTAAATAGCGGTGTCTATACATTTTTAAGCTCAACTCTGAAAAGCCTGGAAGAGAAAGACCATATACATAGAGTGTTGGACAAGATCACT<br>GATACATTGATTCACCTTATGGCGAAAGCAGGATTGACTCTGCAGCAACAGCATCAGAGGCTAGCTCAGTTACTACTAATTTTGTCACACAT<br>TAGACATATGTCTAACAAGGGTATGGAGCACCTGTATTCTATGAAGTGTAAAAACGTCGTTCCTCTGTATGATTTATTACTAGAAATGCTGG<br>ACGCTCACAGGTTGCATGCACCAACTTCTAGAGGCGGAGCCTCCGTTGAAGAAACCGACCAATCCCACCTAGCGACAGCAGGGTCTACAT<br>CTAGCGG**TTCT**TGAGACC
**pWS932 – PRD [3b]**	GGTCTCA**TTCT**AGACCATCTAGTACAACAAAATCAGATAATTCGCCTCCAAAATTAGAAAGCGAGAATTTTAAGGATAATGAGTTGGTAACA<br>GTAACTAATCAGCCGCTTTTAGGCGTTGGCCTCATGGATGACGATGCGCCAGAATCCCCCTCTCAAATTAATGATTTTATTCCTCAGAAATT<br>GATTATAGAACCCAATACTCTCGAATTGAATGGTCTAACAGAAGAAACGCCTCATGACTTACCCAAGAATACCGCTAAGGGCAGAGATGAA<br>GAAGATTTTCCTCTCGACTATTTTCCTGTATCTGTTGAATACCCTACGGAGGAAAATGCGTTTGATCCGTTCCCTCCACAGGCTTTTACGCC<br>AGCTGCCCCTTCCATGCCTATTTCCTATGATAACGTGAATGAAAGGGATTCTATGCCCGTTAATTCTCTTCTTAATAGATACCCCTATCAGTT<br>ATCAGTGGCACCCACTTTCCCAGTGCCACCATCATCATCGAGGCAACATTTTATGACAAATCGGGATTTTTATTCATCTAACAATAACAAGG<br>AAAAATTGGTATCTCCTAGCGACCCTACCAGCTACATGAAGTATGACGAACCAGTTATGGATTTTGATGAATCTCGGCCAAATGAAAACTGT<br>ACAAATGCAAAATCTCACAACTCTGGCCAGCAAACTAAACAACACCAATTATATTCTAACAACTTCCAGCAATCTTACCCAAACGGAATGGT<br>TCCAGGATACTACCCAAAAATGCCGTATAATCCCATGGGGGGGGATCCTCTACTCGATCAAGCCTTTTATGGCGCGGACGATTTTTTCTTT

	CCACCAGAAGGATGTGATAACAATATGCTGTATCCACAAACTGCAACTTCATGGAATGTTTTGCCCCCTCAAGCTATGCAACCAGCTCCAA<br>CCTATGTTGGGAGGCCATACACACCGAATTATAGATCGACACCAGGTTCCGCGATGTTCCCATACATGCAAAGTTCAAATTCCATGCAGTG<br>GAACACTGCTGTTTCACCTTATAGTTCGAGAGCACCATCTACAACTGCTAAAAACTATCCTCCTAGCACATTTTATTCTCAAAATATAAATCA<br>ATACCCACGGCGAAGAACTGTGGGAATGAAGTCATCACAAGGAAATGTTCCAACAGGTAATAAACAATCTGTGGGCAAGTCTGCAAAAATT<br>TCAAAGCCTCTACATATTAAGACAAGTGCTTATCAGAAGCAATACAAAATCAACTTGGAAACGAAAGCCAGGCCAAGTGCTGGTGACGAAG<br>ATTCTGCTCATCCTGATAAGAACAAAGAAATTTCGATGCCTACTCCGGATTCCAATACTTTGGTGGTCCAGTCAGAAGAAGGTGGAGCTCA<br>TTCACTTGAGGTAGATACCAATCGAAGGTCCGATAAAAACCTTCCAGATGCAACCGG**ATCC**TGAGACC
**pWS033 – sfGFP [3]**	GGTCTCA**TATG**TCCAAGGGTGAAGAGCTATTTACTGGGGTTGTACCCATTTTGGTAGAACTGGACGGAGATGTAAACGGACATAAATTCTC<br>TGTTAGAGGTGAGGGCGAAGGCGATGCCACCAATGGTAAATTGACTCTGAAGTTTATATGCACTACGGGTAAATTACCTGTTCCTTGGCCA<br>ACCCTAGTAACAACTTTGACATATGGTGTTCAATGTTTCTCAAGATACCCAGACCATATGAAAAGGCATGATTTCTTTAAAAGTGCTATGCCA<br>GAAGGCTACGTGCAAGAGAGAACTATCTCCTTTAAGGATGACGGTACGTATAAAACACGAGCAGAAGTGAAATTCGAAGGGGATACACTA<br>GTTAATCGCATCGAATTAAAGGGTATAGACTTTAAGGAAGATGGTAATATTCTCGGCCATAAACTTGAGTATAATTTCAACTCGCATAATGTG<br>TACATTACAGCTGACAAACAAAAGAACGGAATTAAAGCGAATTTTAAAATCAGGCACAACGTCGAAGATGGGTCTGTTCAACTTGCCGATCA<br>TTATCAGCAAAACACCCCTATTGGTGATGGTCCAGTCTTGTTACCCGATAATCACTACTTAAGCACACAGTCTAGATTGTCAAAAGATCCGA<br>ATGAAAAGCGTGATCACATGGTTTTATTGGAATTTGTCACCGCTGCAGGAATAACTCACGGAATGGACGAGCTTTATAAGGG**ATCC**TGAGA<br>CC
**pRC063 – mTagBFP2 [3]**	GGTCTCA**TATG**AGTGAGTTGATTAAGGAAAACATGCACATGAAGTTGTACATGGAAGGAACAGTAGATAATCATCATTTCAAATGTACGTCA<br>GAAGGTGAGGGCAAACCTTATGAAGGAACTCAAACTATGAGAATTAAAGTCGTTGAGGGTGGCCCACTACCATTTGCATTCGATATCCTCG<br>CAACATCTTTTCTGTATGGTTCTAAAACCTTTATCAACCATACACAAGGCATACCAGATTTCTTTAAGCAATCATTTCCTGAAGGTTTCACAT<br>GGGAAAGAGTAACTACCTATGAAGATGGTGGTGTCCTTACAGCTACTCAAGACACGTCTTTGCAGGATGGTTGTTTAATCTACAATGTTAAG<br>ATAAGAGGAGTGAACTTTACATCCAATGGACCAGTCATGCAAAAGAAAACTCTAGGTTGGGAGGCATTCACAGAAACTTTGTACCCAGCCG<br>ATGGGGGCTTAGAAGGGAGAAATGACATGGCTTTAAAACTGGTTGGCGGTAGCCATTTGATTGCGAACGCTAAAACAACCTACAGAAGCA<br>AAAAGCCAGCTAAAAATCTTAAGATGCCTGTATACTACGTGGATTACAGATTAGAAAGGAAGGAAGCTAATAATGAAACTTACGTTGAACAG<br>CACGAGGTGGCCGTTGCCCGTTATTGCGACCTTCCTTCAAAATTGGGACACAAACTAAACGG**ATCC**TGAGACC
**pWS1837 – α-factor [3-4a]**	GGTCTCA**TATG**AGATTTCCTTCAATTTTTACTGCAGTTTTATTCGCAGCATCCTCCGCATTAGCTGCTCCAGTCAACACTACAACAGAAGAT<br>GAAACGGCACAAATTCCGGCTGAAGCTGTCATCGGTTACTTAGATTTAGAAGGGGATTTCGATGTTGCTGTTTTGCCATTTTCCAACAGCA<br>CAAATAACGGGTTATTGTTTATAAATACTACTATTGCCAGCATTGCTGCTAAAGAAGAAGGGGTATCTTTGGATAAAAGAGAGGCTGAAGCT<br>TGGCATTGGTTGCAACTAAAACCTGGCCAACCAATGTACAAGAGAGAAGCCGAAGCTGAAGCTTGGCATTGGCTGCAACTAAAGCCTGGC<br>CAACCAATGTACAAAAGAGAAGCCGACGCTGAAGCTTGGCATTGGCTGCAACTAAAGCCTGGCCAACCAATGTACAAAAGAGAAGCCGAC<br>GCTGAAGCTTGGCATTGGTTGCAGTTAAAACCCGGCCAACCAATGTACTAACTCGAG**TGGC**TGAGACC
**pWS788 – Bar1 [3]**	GGTCTCA**TATG**TCTGCAATTAATCATCTTTGTTTGAAACTTATTTTGGCGAGTTTCGCGATTATTAACACCATTACTGCTTTAACAAACGATG<br>GCACTGGTCACTTAGAATTCCTTTTACAACACGAAGAGGAGATGTATTACGCAACAACCTTAGATATAGGTACACCGTCCCAAAGTCTGACA<br>GTGTTGTTTGATACCGGATCTGCCGATTTTTGGGTTATGGATTCTAGCAATCCCTTCTGCTTACCAAATTCAAATACGTCATCCTATTCAAAC<br>GCAACTTATAATGGCGAAGAAGTTAAGCCTTCAATTGATTGCAGGTCTATGAGTACTTATAATGAGCATAGATCTTCCACCTACCAATATCT<br>GGAAAATGGTAGGTTTTACATCACATATGCTGACGGAACATTTGCTGACGGTAGTTGGGGGACGGAAACTGTATCAATTAATGGAATTGAC<br>ATCCCCAATATCCAGTTCGGAGTTGCCAAGTATGCTACGACACCCGTTAGTGGTGTTCTTGGAATTGGGTTTCCTAGAAGAGAGTCCGTTA<br>AGGGCTATGAAGGTGCTCCTAATGAATATTATCCTAATTTTCCTCAGATTTTAAAAAGTGAAAAAATAATCGATGTGGTCGCGTATTCGCTGT<br>TCTTAAACTCACCTGATTCAGGTACTGGTTCGATTGTTTTTGGTGCCATTGATGAATCAAAGTTTTCTGGTGATTTGTTCACTTTCCCTATGG<br>TAAATGAATATCCCACAATAGTCGACGCTCCTGCAACTTTAGCAATGACTATACAAGGATTAGGTGCCCAAAACAAAAGTAGTTGTGAACAT<br>GAAACGTTTACGACGACCAAGTATCCAGTTTTGTTGGACTCAGGAACCTCGCTATTGAATGCGCCCAAGGTCATAGCAGATAAAATGGCTT<br>CTTTTGTAAATGCGTCCTATAGTGAAGAGGAAGGTATATATATATTAGACTGTCCAGTATCTGTAGGTGACGTGGAATACAATTTTGATTTCG<br>GCGATTTGCAAATAAGTGTTCCACTGTCTAGTTTGATTTTAAGTCCCGAGACAGAAGGCAGCTATTGTGGGTTTGCGGTCCAGCCAACAAA<br>CGATTCGATGGTTCTGGGTGATGTGTTCCTGTCCTCTGCATACGTCGTATTCGATCTCGATAATTATAAGATATCTTTAGCACAGGCAAATT<br>GGAACGCAAGCGAAGTTTCGAAAAAGCTAGTAAATATTCAAACAGATGGGTCTATTTCAGGTGCCAAAATTGCTACAGCTGAACCCTGGTC<br>CACCAATGAACCATTTACAGTCACCTCTGACATTTATTCATCTACAGGCTGCAAGAGTAGGCCTTTTCTTCAATCATCGACAGCCTCTTCGC<br>TTATTGCAGAAACCAACGTACAAAGTCGCAACTGCTCTACGAAGATGCCAGGCACTAGATCAACTACTGTCTTAAGTAAGCCTACTCAAAAT<br>AGTGCTATGCATCAAAGTACAGGCGCTGTCACACAAACCTCAAATGAAACTAAATTAGAATTATCCTCGACTATGGCAAATTCGGGCAGTGT<br>CTCGCTTCCCACTTCGAATTCAATAGACAAAGAGTTCGAACATTCGAAATCTCAAACTACCAGCGATCCAAGTGTAGCAGAGCATTCTACGT<br>TTAACCAAACGTTTGTACATGAAACTAAATATCGGCCTACTCATAAGACAGTCATAACAGAAACTGTCACGAAGTATTCTACAGTCTTAATAA<br>ATGTCTGTAAACCAACATATGG**ATCC**TGAGACC
**pWS081 – Sst2 [3]**	GGTCTCA**TATG**GTTGACAAGAACAGGACATTGCATGAATTGTCATCTAAGAATTTCAGTAGGACGCCAAATGGTCTTATCTTTACAAATGAT<br>CTAAAAACCGTATATTCAATATTTTTGATTTGCCTTGACCTAAAGGAAAAGAAACACAGTTCAGATACTAAATCCTTTCTACTAACAGCTTTCA<br>CCAAACATTTCCATTTTACATTCACGTATCAGGAGGCAATCAAGGCCATGGGTCAACTGGAACTAAAAGTGGATATGAATACTACGTGCATC<br>AATGTGAGTTATAATATTAAGCCGAGCTTAGCAAGACATCTACTAACCCTATTCATGAGTTCAAAGTTATTACATACGCCTCAAGATCGTACA<br>AGAGGTGAGCCCAAGGAGAAGGTATTATTCCAACCTACGCCCAAAGGTGTCGCGGTTCTGCAGAAATACGTCAGAGACATTGGTCTAAAG<br>ACCATGCCGGACATATTGCTGTCTAGTTTCAACTCTATGAAGCTGTTTACATTCGAGCGTTCTTCCGTTACTGACAGCATTATTCATTCAGA<br>CTATCTGATACATATCCTATTCATTAAAATGATGGGTGCTAAGCCCAACGTTTGGAGCCCAACCAATGCCGACGACCCTCTGCCATGCCTG<br>AGTAGTTTATTAGAGTATACGAATAACGACGACACATTTACCTTCGAAAAGTCTAAACCGGAGCAGGGATGGCAAGCTCAAATCGGAAACA<br>TAGACATCAATGATCTAGAGAGAGTCTCCCCGCTTGCCCACAGGTTTTTTACGAACCCTGATAGTGAATCCCACACTCAATATTATGTAAGT<br>AATGCTGGTATAAGACTTTTCGAAAATAAGACGTTCGGCACGTCAAAGAAGATAGTGATTAAATACACGTTTACGACAAAAGCAATTTGGCA<br>ATGGATCATGGACTGCACAGATATAATGCATGTAAAAGAGGCAGTGTCCCTAGCCGCACTTTTCTTGAAAACAGGATTGATCGTACCCGTA<br>CTATTGCAACCAAGCCGTACGGATAAGAAAAAATTTCAAATCAGTAGGTCTAGCTTCTTCACCCTTAGCAAACGTGGCTGGGATCTAGTATC<br>CTGGACCGGGTGCAAGTCTAACAATATTCGTGCACCAAACGGCTCCACGATAGACCTGGACTTTACCCTGAGAGGCCATATGACAGTTAG<br>GGACGAGAAAAAGACTCTAGATGATTCTGAAGGCTTTAGCCAAGATATGTTGATCTCAAGCAGCAATCTTAACAAATTAGACTACGTGCTAA<br>CTGATCCTGGCATGCGTTACTTGTTCCGTAGACATCTGGAGAAAGAGCTTTGCGTGGAGAATTTAGATGTTTTCATTGAGATAAAACGTTTC<br>CTAAAGAAGATGACCATTTTAAAAAAACTGATCGATTCAAAGCACTGCGATAAAAAGTCCAATACGAGCACAAGTAAGAATAACATCGTGAA<br>AACCATCGATAGTGCCTTAATGAAGCAGGCCAACGAGTGTTTGGAAATGGCCTATCACATCTATTCCTCTTATATAATGATTGGAAGTCCAT<br>ATCAATTGAACATTCATCACAACTTGAGACAGAACATTTCTGACATTATGTTACACCCACATAGTCCTTTGTCAGAACACTTTCCAACGAATC<br>TGTACGATCCATCCCCCGCCAGCGCCGAGTCCGCTGCATCTTCAATTTCTAGTACAGAGGCGGACACCCTTGGCGAGCCACCAGAAGTGA<br>GCCTGAAACCAAGTAAGAACCTGAGCAATGAAAATTGCTCATTCAAGAAGCAGGGTTTCAAACATCAGTTGAAAGAATATAAGCCTGCCCC<br>CTTAACGCTGGCGGAGACTCATTCCCCTAACGCTAGTGTGGAAAACAGCCATACGATCGTGAGATATGGTATGGACAATACGCAGAACGA<br>TACGAAGTCAGTGGAATCATTTCCTGCTACCCTTAAAGTATTAAGGAAGCTTTACCCCTTATTTGAAATAGTGTCAAATGAGATGTATAGGTT<br>GATGAACAACGACTCCTTCCAAAAATTTACCCAGAGTGATGTTTACAAGGACGCTTCTGCCTTGATTGAAATACAGGAAAAATGCGGATCCT<br>GAGACC
**pWS509 – Msg5 [3]**	GGTCTCC**TATG**CAATTTCACTCAGATAAGCAGCATTTGGACAGTAAAACCGACATCGATTTCAAGCCAAACTCACCGCGTTCCTTACAAAAT<br>AGGAATACCAAAAATTTATCTTTAGATATAGCAGCACTCCATCCATTAATGGAATTCTCATCGCCAAGCCAAGATGTGCCAGGTTCAGTAAA<br>ATTCCCATCGCCGACACCTTTGAATCTATTTATGAAGCCCAAACCTATTGTGTTGGAGAAATGTCCACCAAAAGTAAGTCCAAGGCCAACG<br>CCACCATCGCTGTCGATGAGGCGAAGCGAGGCCTCTATATACACACTACCAACATCTTTGAAGAACCGAACTGTTTCTCCAAGCGTGTATA<br>CAAAATCATCCACAGTATCGTCTATCAGTAAGCTGTCATCATCATCACCGTTATCGTCATTTTCAGAAAAACCTCATCTGAATAGAGTCCATT<br>CATTATCCGTGAAAACTAAAGACTTGAAGTTGAAGGGAATTAGAGGACGTTCTCAAACGATCTCAGGGTTAGAGACATCCACGCCAATTTC<br>TAGTACTCGTGAAGGTACTTTAGATAGTACGGATGTCAACAGATTTTCTAACCAAAAGAATATGCAAACAACATTGATTTTCCCGGAGGAGG<br>ACTCGGATCTGAATATTGATATGGTGCATGCAGAGATTTATCAACGAACGGTTTATTTAGATGGACCATTGCTGGTACTGCCGCCTAATTTG<br>TATCTATATTCAGAACCCAAACTAGAAGATATATTATCGTTTGATTTAGTCATTAATGTTGCCAAAGAAATACCGAACCTGGAGTTTTAATAC<br>CGCCGGAAATGGCACATAAAATAAAATATTACCATATTGAATGGACACACACATCCAAGATCGTCAAGGACTTATCCCGATTGACACGCATT<br>ATACATACCGCTCATTCGCAAGGCAAGAAAATACTCGTACACTGTCAGTGTGGAGTATCAAGATCGGCGTCATTGATTGTGGCGTATATCA<br>TGCGATATTATGGCTTGAGTTTAAATGATGCATACAATAAGCTGAAAGGTGTTGCTAAGGATATAAGTCCAAACATGGGTCTGATCTTCCAA

	CTTATGGAATGGGGAACCATGTTGTCCAAGAACTCACCGGGCGAAGAAGGAGAGACTGTTCACATGCCTGAGGAAGATGACATCGGAAAC<br>AACGAAGTTTCCTCGACTACGAAGTCCTACTCTTCTGCGTCCTTTAGAAGTTTTCCCATGGTAACGAATCTATCATCGTCGCCGAATGACAG<br>TTCTGTCAATTCTTCGGAAGTAACGCCAAGAACTCCTGCTACGTTGACTGGAGCAAGGACCGCACTGGCCACAGAACGCGGGGAAGATGA<br>TGAGCACTGTAAAAGTTTGTCTCAACCCGCAGATTCACTGGAAGCTTCTGTGGACAACGAATCAATATCTACTGCCCCGGAACAGATGATG<br>TTTCTTCCTGG**ATCC**TGAGACC
**pWS510 – Dig1 [3] (Internal BpiI sites)**	GGTCTCCT**ATG**GCCGTATCAGCCCGTTTGAGAACGACTGCCGAGGATACATCCATTGCTAAATCAACACAAGATCCAATTGGTGATACAGA<br>AATCAGTGTAGCAAATGCTAAGGGCAGCAGCGATAGCAACATTAAGAATTCGCCAGGCGGAAACAGCGTTGGTCAGGAGTCGGAGCTAGA<br>GCATGTCCCTGAGGAGGATGACTCTGGTGACAAGGAAGCAGATCATGAGGATTCTGAGACAGCCACTGCGAAGAAGAGGAAAGCCCAAC<br>CATTGAAGAATCCGAAGAAATCATTGAAGAGGGGCAGAGTCCCGGCGCCTTTGAATTTATCGGATAGCAACACTAATACACACGGTGGTAA<br>TATTAAGGACGGCAACTTGGCTTCGTCTAACTCTGCACATTTTCCTCCTGTTGCTAATCAAAACGTGAAAAGCGCGCCCGCACAAGTTACT<br>CAGCATTCCAAGTTCCAGCCCCGTGTCCAATACTTGGGAAAGGCCAGTTCTAGACAATCTATACAAGTGAATAATAGCAGCAATAGTTATG<br>GGAAACCACACATGCCCTCGGCGGGCATCATGAGCGCCATGAACCCTTACATGCCCATGAATCGCTACATAATGTCACCATATTATAATCC<br>GTACGGTATCCCTCCACCTCACATGCTGAACAAGCCCATAATGACACCCTACGTGTCGTATCCATATCCAATGGGACCGCGGACCTCCATT<br>CCTTATGCTATGCAAGGTGGCAACGCTAGGCCCTACGAAGAAAATGAGTATAGTGCTAGCAATTACAGAAACAAGAGGGTTAACGACTCAT<br>ATGATTCGCCTTTGAGTGGCACCGCTTCCACTGGGAAAACCAGACGATCCGAGGAAGGCTCAAGAAATTCTAGCGTAGGATCAAGTGCGA<br>ATGCCGGTCCTACGCAACAGCGCGCGGATCTACGCCCAGCAGATATGATACCTGCTGAAGAATACCACTTTGAACGGGATGCATTACTCT<br>CGGCCAACACGAAAGCCAGAAGCGCAAGCACAAGCACAAGTACAAGCACAAGCACAAACCGGGACAGGAGTTCATGGCATGAGGCAGAA<br>CCCAATAAAGACGAAGAAGAGGGTACGGACTTGGCCATCGAGGACGGAGCGGTCCCCACTCCCACTTTTACTACGTTCCAGCGGACCTC<br>GCAGCCGCAGCAGCAATCACCTAGTCTTCTTCAAGGCGAAATCCGACTCTCATCGCATATTTTTGCCTTCGAGTTCCCCCTGAGCTCTAGC<br>AATGTAGACAAGAAAATGTTTATGAGCATATGTAATAAAGTATGGAATGAATCAAAGGAGCTGACAAAAAAATCATCATCACATCACAGAAC<br>CGGAAAAGG**ATCC**TGAGACC
**pWS511 – Dig2 [3] (Internal BpiI sites)**	GGTCTCCT**ATG**AATAAAGAAGAGCAAGAAGACCCACAGCAAGAGCAAATATCAACTGTTCAGGAAAATGATCCCAGGAATTTGCAGCAACT<br>GGGAATGCTGTTAGTATCTCCAGGGCTTGATGAAGACAGATTGAGTGAGAAAATGATTTCGAAGATCAAAAAATCCAGGGATATCGAAAAA<br>AATCAAAAATTGCTCATATCTAGGCTGTCGCAAAAAGAGGAAGATCATAGTGGTAAGCCTCCTACCATTACGACTTCTCCAGCAGAGAAAA<br>CCGTACCCTTTAAGTCGCTGAATCATTCTTTAAAGAGGAAAAGGGTACCTCCAGCGCTAAATTTTTCCGATATACAAGCATCTTCTCATTTG<br>CATGGATCTAAAAGTGCTCCACCAAACATAACAAGATTTCCGCAGCACAAAAATAGCCTTAGGGTCAGATATATGGGTAGGATGGCTCCTA<br>CGAATCAAGATTATCATCCTTCAGTGGCCAATTCATATATGACAGCAACCTACCCTTATCCATATACTGGACTGCCACCAGTACCATGCTAC<br>CCATATTCTTCAACTCCAACACAAACGCACGCATACGAAGGCTATTATTCCCCGATGTATCCCGGCCCCTTGTATAATAATGGTATAATACC<br>AGCCGACTACCATGCAAAGAGGAAGAAGTTGGCTGGTAGATCACCACACTTGGAAGATTTGACATCGAGAAAAAGAACCTTTGTCTCCAAA<br>CACCACAACGGAGATCCAATCATAAGTAAGACTGATGAAGACATTGAATGCTCTGTCACGAAAAATTCATTAAGTGAGGGCGCTTCACTTAA<br>CGACGATGCCGATGATGACAACGACAAAGAAAGGATCATTATTGGAGAAATCTCTCTGTATGATGATGTTTTCAAATTTGAAGTTCGCGACG<br>ACAAAAATGACTATATGAAAGCATGTGAAACAATCTGGACTGAATGGCATAACTTGAAGAAAGG**ATCC**TGAGACC
**pWS627 – Ste4-2A-Ste18 [3-4a] (Internal BpiI sites)**	GGTCTCAT**ATG**GCAGCACATCAGATGGACTCGATAACGTATTCTAATAATGTCACCCAACAGTATATACAACCACAAAGTCTACAGGATATC<br>TCTGCAGTGGAGGATGAAATTCAAAATAAAATAGAGGCCGCCAGACAAGAGAGTAAACAGCTTCATGCTCAAATAAATAAAGCAAAACACA<br>AGATACAAGATGCAAGCTTATTCCAGATGGCCAACAAAGTTACTTCGTTGACCAAAAATAAGATCAACTTAAAGCCAAATATCGTGTTGAAA<br>GGCCATAATAATAAAATCTCAGATTTTCGGTGGAGTCGAGATTCAAAACGTATTTTGAGTGCAAGTCAAGATGGCTTTATGCTTATATGGGA<br>CAGTGCTTCAGGTTTAAAACAGAACGCTATTCCATTAGATTCTCAATGGGTTCTTTCCTGCGCTATTTCGCCATCGAGTACTTTGGTAGCAA<br>GCGCAGGATTAAACAATAACTGTACCATTTATAGAGTTTCGAAAGAAAACAGAGTAGCGCAAAACGTTGCGTCAATTTTCAAAGGACATACT<br>TGCTATATTTCTGACATTGAATTTACAGATAACGCACATATATTGACAGCAAGTGGGGATATGACATGTGCCTTGTGGGATATACCGAAAGC<br>AAAGAGGGTGAGAGAATATTCTGACCATTTAGGTGATGTTTTGGCATTAGCTATTCCTGAAGAGCCAAACTCAGAAAATTCTTCGAACACAT<br>TCGCTAGCTGTGGATCAGACGGGTATACTTACATATGGGATAGCAGATCTCCGTCCGCTGTACAAAGCTTTTACGTTAACGATAGTGATATT<br>AATGCACTTCGTTTTTTCAAAGACGGGATGTCGATTGTTGCAGGAAGTGACAATGGTGCGATAAATATGTATGATTTAAGGTCGGACTGTTC<br>TATTGCTACTTTTTCTCTTTTTCGAGGTTATGAAGAACGTACCCCTACCCCTACTTATATGGCAGCTAACATGGAGTACAATACCGCGCAAT<br>CGCCACAAACTTTAAAATCAACAAGCTCAAGCTATCTAGACAACCAAGGCGTTGTTTCTTTAGATTTTAGTGCATCTGGAAGATTGATGTAC<br>TCATGCTATACAGACATTGGTTGTGTTGTGTGGGATGTATTAAAAGGAGAGATTGTTGGAAAATTAGAAGGTCATGGTGGCAGAGTCACTG<br>GTGTGCGCTCGAGTCCAGATGGGTTAGCTGTATGTACAGGTTCATGGGACTCAACCATGAAAATATGGTCACCAGGTTATCAAGAAGCTAG<br>ACATAAACAAAAGATTGTTGCTCCAGTTAAACAAACTTTGAACTTTGATTTGTTGAAATTGGCTGGTGATGTTGAATCTAATCCAGGGCCCA<br>CATCAGTTCAAAACTCTCCACGCTTACAACAACCTCAGGAACAGCAACAGCAACAGCAACAGCTTTCCTTAAAGATAAAACAATTGAAGTTA<br>AAAAGAATCAACGAACTTAACAATAAACTGAGGAAAGAACTCAGCCGTGAAAGAATTACTGCTTCAAATGCATGTCTTACAATAATAAACTAT<br>ACCTCGAATACAAAAGATTATACATTACCAGAACTATGGGGCTACCCCGTAGCAGGATCAAATCATTTTATAGAGGGTTTGAAAAATGCTCA<br>AAAAAATAGCCAAATGTCAAACTCAAATAGTGTTTGTTGTACGCTTATGTAACTCGAG**TGGC**TGAGACC
**pWS1859 – Ste50 [3] (Internal BpiI sites)**	GGTCTCAT**ATG**GAGGACGGTAAACAGGCCATCAATGAGGGATCAAACGATGCTTCGCCGGATCTGGACGTGAATGGCACAATATTGATGA<br>ATAATGAAGACTTTTCCCAGTGGTCGGTTGATGATGTGATAACTTGGTGTATATCCACGCTGGAGGTGGAAGAAACCGATCCATTATGTCA<br>GAGACTGCGAGAAAATGATATTGTAGGAGATCTTTTGCCGGAATTGTGCTTGCAAGATTGCCAGGACTTGTGTGACGGTGATTTGAATAAG<br>GCCATAAAATTCAAGATACTGATCAATAAGATGAGAGACAGCAAGTTGGAGTGGAAGGACGACAAGACTCAAGAGGACATGATAACGGTA<br>CTGAAAAACTTGTACACTACTACATCTGCGAAATTGCAAGAATTTCAATCGCAGTACACAAGGCTGAGGATGGATGTCTTGGACGTAATGAA<br>GACCAGCTCAAGCTCTTCTCCGATTAACACACATGGAGTGTCCACTACGGTACCTTCTTCAAACAACACAATTATACCCAGTAGTGACGGT<br>GTGTCTCTTTCACAAACAGACTATTTCGACACAGTTCATAACCGACAATCACCGTCAAGGAGAGAATCCCCGGTAACGGTATTTAGGCAAC<br>CCAGTCTTTCCCACTCAAAATCTTTGCACAAGGATAGCAAAAACAAAGTACCCCAAATATCTACAAACCAATCTCACCCATCTGCCGTTTCA<br>ACAGCGAACACACCGGGGCCATCACCTAACGAGGCGTTAAAACAGTTGCGTGCATCTAAAGAAGACTCCTGCGAACGGATCTTGAAAAAC<br>GCAATGAAAAGACATAACTTAGCAGATCAGGATTGGAGACAATATGTCTTGGTCATTTGCTATGGGGATCAAGAGAGGCTGTTAGAATTGA<br>ACGAAAAGCCTGTGATCATATTCAAGAACTTAAAGCAACAGGGTTTGCACCCCCGCCATTATGTTAAGAAGAAGAGGTGATTTCGAAGAAGT<br>AGCAATGATGAACGGAAGTGACAATGTCACCCCCGGTGGAAGATTGGG**ATCC**TGAGACC
