# Supplementary material for: Engineering a Model Cell for Rational Tuning of GPCR Signaling
Source: Cell. 2019 Apr 18;177(3):782–796.e27. doi: 10.1016/j.cell.2019.02.023 (PMC6476273; doi:10.1016/j.cell.2019.02.023)
Supplement: Methods S1. Model Schematics, Related to STAR Methods — (A) The detailed reaction scheme of the cubic ternary complex model modified for the refactored S. cerevisiae pathway. Reactions can be divided into modules of which involves Receptor activation (Top) and G protein cycle (Bottom). Black letters each represent a species within a system, and all arrows are marked in red letters with rate constants k● and corresponding cooperativity factors μ, ν, and ζ, which produce a unique rate constant for each reaction. (B) The detailed reaction scheme of the single ternary complex model modified for the refactored S. cerevisiae pathway. Reactions can be divided into modules of which involves Receptor activation (Top), G protein cycle (Middle), and the downstream cascade (Bottom). Black letters each represent a species within a system, and all arrows are marked in red letters with rate constants k● and corresponding cooperativity factors μ, ν, and ζ, which produce a unique rate constant for each reaction. (C) Schematic diagram of the double cell system. (Left) Signaling system within a cell. Each cell contains the corresponding set of kinetic rates and species. (Right) Simplified version of the full system, including the interaction of Bar1 and α-factor and its degradation. (D) Overall fitting results against time course data as concentration-response curves taken through endpoint readings. Solid shapes represent experimental results reading fluorescence 260 minutes after the addition of ligand. Solid lines represent ODE results derived from time courses of 260 minutes. Blue represents the system with the α-factor producing cell only and red represents the system with two cells with Bar1. (E) Residual plots of the experimental concentration-response curves against the computational fitting results. (Left) Residual plots of the MTNR1A sensor. (Middle) Residual plots of the experimental results of digital feedback without Bar1. (Right) Residual plots of the experimental results of digital feedback with Bar1. (F) Abbreviations used i [file mmc5.pdf]

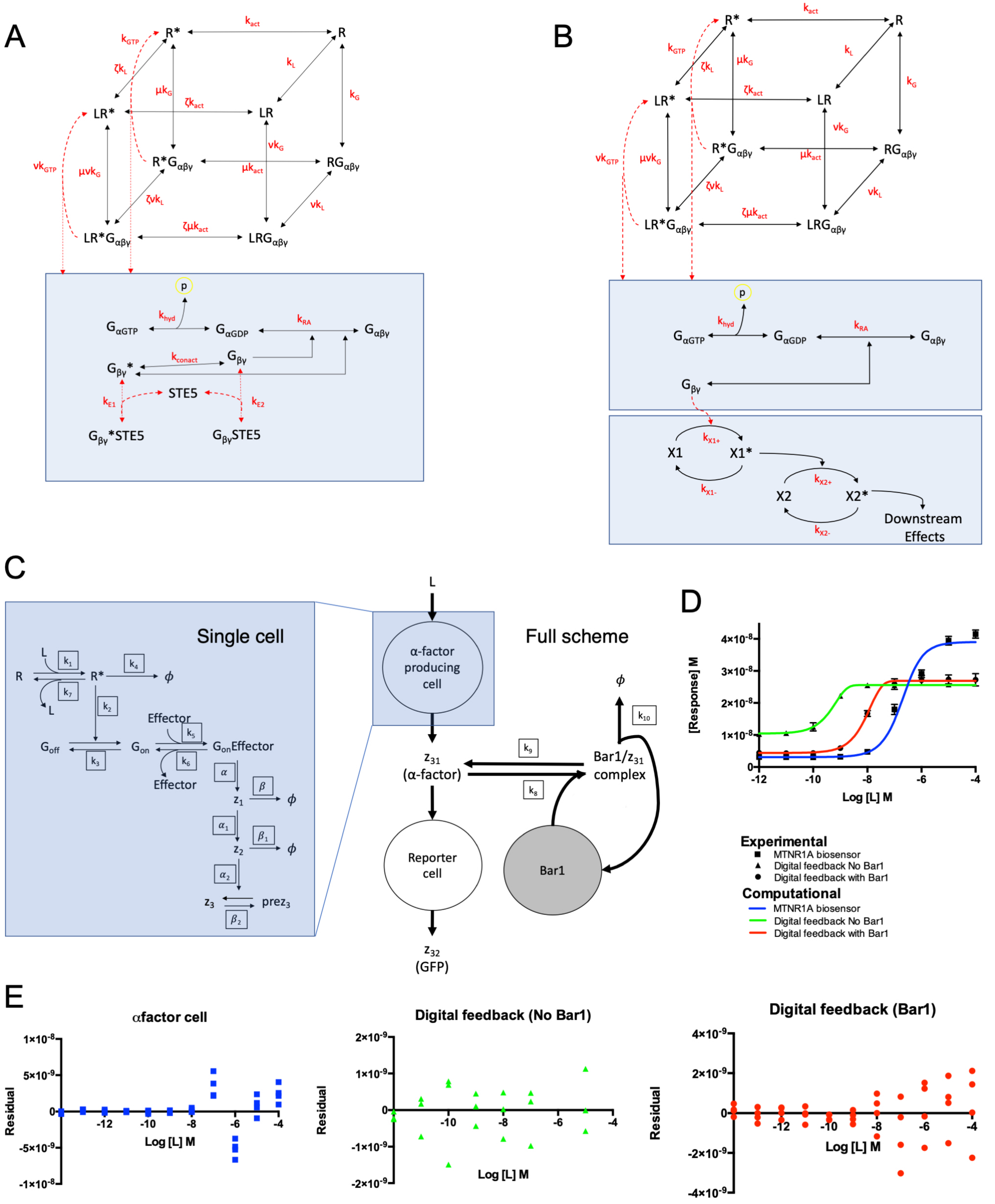

F

Abbreviations used

L	Ligand	$G_{\alpha GDP}$	Inactive α subunit
R	Unbound Receptor	$G_{\alpha GTP}$	Active α subunit
R^*	Activated Receptor	X1, X2	Inactive species for transcriptional delay
$G_{\alpha\beta\gamma}$	Heterotrimeric G protein	X1*, X2*	Active species for transcriptional delay
$G_{\beta\gamma}$	βɣ subunit	G_{off}	Inactive G protein
$G_{\beta\gamma}^*$	βɣ subunit with downstream effector	G_{on}	Active G protein
		Z	Arbitrary species to simulate transcriptional decay
